# Cross-reactive antibodies against human coronaviruses and the animal coronavirome suggest diagnostics for future zoonotic spillovers

**DOI:** 10.1126/sciimmunol.abe9950

**Published:** 2021-07-29

**Authors:** Shelley Klompus, Sigal Leviatan, Thomas Vogl, Roei D. Mazor, Iris N. Kalka, Liat Stoler-Barak, Nachum Nathan, Ayelet Peres, Lihee Moss, Anastasia Godneva, Sharon Kagan Ben Tikva, Eilat Shinar, Hadas Cohen-Dvashi, Ronen Gabizon, Nir London, Ron Diskin, Gur Yaari, Adina Weinberger, Ziv Shulman, Eran Segal

**Affiliations:** 1 Department of Computer Science and Applied Mathematics, Weizmann Institute of Science, Rehovot 76100, Israel.; 2 Department of Molecular Cell Biology, Weizmann Institute of Science, Rehovot 76100, Israel.; 3 Department of Immunology, Weizmann Institute of Science, Rehovot 76100, Israel.; 4 Faculty of Engineering, Bar Ilan University, Ramat Gan 52900, Israel.; 5 Magen David Adom Blood Services, Ramat Gan 52900, Israel.; 6 Department of Chemical and Structural Biology, Weizmann Institute of Science, Rehovot 7610001, Israel.

## Abstract

The spillover of animal coronaviruses (aCoVs) to humans has caused SARS, MERS, and COVID-19. Although antibody responses displaying cross-reactivity between SARS-CoV-2 and seasonal/common cold human coronaviruses (hCoVs) have been reported, potential cross-reactivity with aCoVs and the diagnostic implications are incompletely understood. Here, we probed for antibody binding against all 7 hCoVs and 49 aCoVs represented as 12,924 peptides within a phage-displayed antigen library. Antibody repertoires of 269 recovered patients with COVID-19 showed distinct changes compared with 260 unexposed prepandemic controls, not limited to binding of SARS-CoV-2 antigens but including binding to antigens from hCoVs and aCoVs with shared motifs to SARS-CoV-2. We isolated broadly reactive monoclonal antibodies from recovered patients with COVID-19 who bind a shared motif of SARS-CoV-2, hCoV-OC43, hCoV-HKU1, and several aCoVs, demonstrating that interspecies cross-reactivity can be mediated by a single immunoglobulin. Using antibody binding data against the entire CoV antigen library allowed accurate discrimination of recovered patients with COVID-19 from unexposed individuals by machine learning. Leaving out SARS-CoV-2 antigens and relying solely on antibody binding to other hCoVs and aCoVs achieved equally accurate detection of SARS-CoV-2 infection. The ability to detect SARS-CoV-2 infection without knowledge of its unique antigens solely from cross-reactive antibody responses against other hCoVs and aCoVs suggests a potential diagnostic strategy for the early stage of future pandemics. Creating regularly updated antigen libraries representing the animal coronavirome can provide the basis for a serological assay already poised to identify infected individuals after a future zoonotic transmission event.

## Teaser

Cross-reactive antibodies present after SARS-CoV-2 infection can distinguish patients with COVID-19 from uninfected individuals.

## INTRODUCTION

COVID-19 (coronavirus disease 2019), caused by SARS-CoV-2 (severe acute respiratory syndrome coronavirus 2), represents a pandemic with millions of cases worldwide. The related b coronaviruses (CoVs) SARS-CoV and Middle East respiratory syndrome (MERS)–CoV were the cause of the SARS outbreak in 2003 and MERS in 2012 (*1*). These three highly pathogenic CoVs are believed to represent spillovers of animal CoVs (aCoVs) to humans, with bats as the initial source (*2*, *3*). Additional intermediate animal hosts have possibly contributed to the transmission to humans, including palm civets and racoon dogs for SARS-CoV and camels for MERS-CoV. The intermediate host of SARS-CoV-2 is unclear, with a potential involvement of pangolins (*4*–*6*).

Given the large reservoir of aCoVs in the wild (i.e., the animal coronavirome) (*2*) and the possibility of recombination events leading to variants with an altered host spectrum (*6*), it has been speculated that more zoonotic transmissions of aCoVs to humans could happen in the future (*2*). To this end, broadly neutralizing vaccines targeting conserved regions of CoVs (*7*, *8*) and diagnostics for assessing their spread in humans could represent critical tools to counteract potential future pandemics. Serological assays based on antibody responses against pathogens are invaluable to inform about the population-wide exposure to a pandemic (*9*, *10*). While testing on the basis of the detection of viral nucleic acids informs about acute infections, antibody tests allow assessment of past exposure and can thereby reveal the contribution of asymptomatic cases possibly undetected by nucleic acid–based testing. The rapid availability of accurate serological tests (as well as access to prepandemic controls representing baseline antibody repertoires) could be key to increase the preparedness for future pandemics caused by zoonotic spillovers to humans (*11*).

However, the accuracy of serological tests can be perturbed by antibody cross-reactivity with similar antigens. Multiple CoV strains infect humans (hCoVs). In addition to SARS-CoV-2, SARS-CoV, and MERS-CoV, the seasonal endemic hCoVs (OC43, HKU1, NL63, and 229E) are widely circulating in the population (*2*). Previous exposures to seasonal hCoVs could affect the accuracy of serological tests and potentially eliciting immunological memory that could affect the course of SARS-CoV-2 infections. Although increasing amounts of data are accumulating on antibody cross-reactivity between hCoVs (*12*, *13*), cross-reactivity with the animal coronavirome and its diagnostic potential for detecting future spillovers of aCoVs to humans is incompletely understood. In addition, the mechanism of cross-reactivity between hCoVs has not been characterized in detail. It remains unclear whether multiple antibodies in patients’ sera target different CoV-derived peptides or single monoclonal antibodies (mAbs) can mediate cross-reactivity between CoVs.

**Fig. 1.**
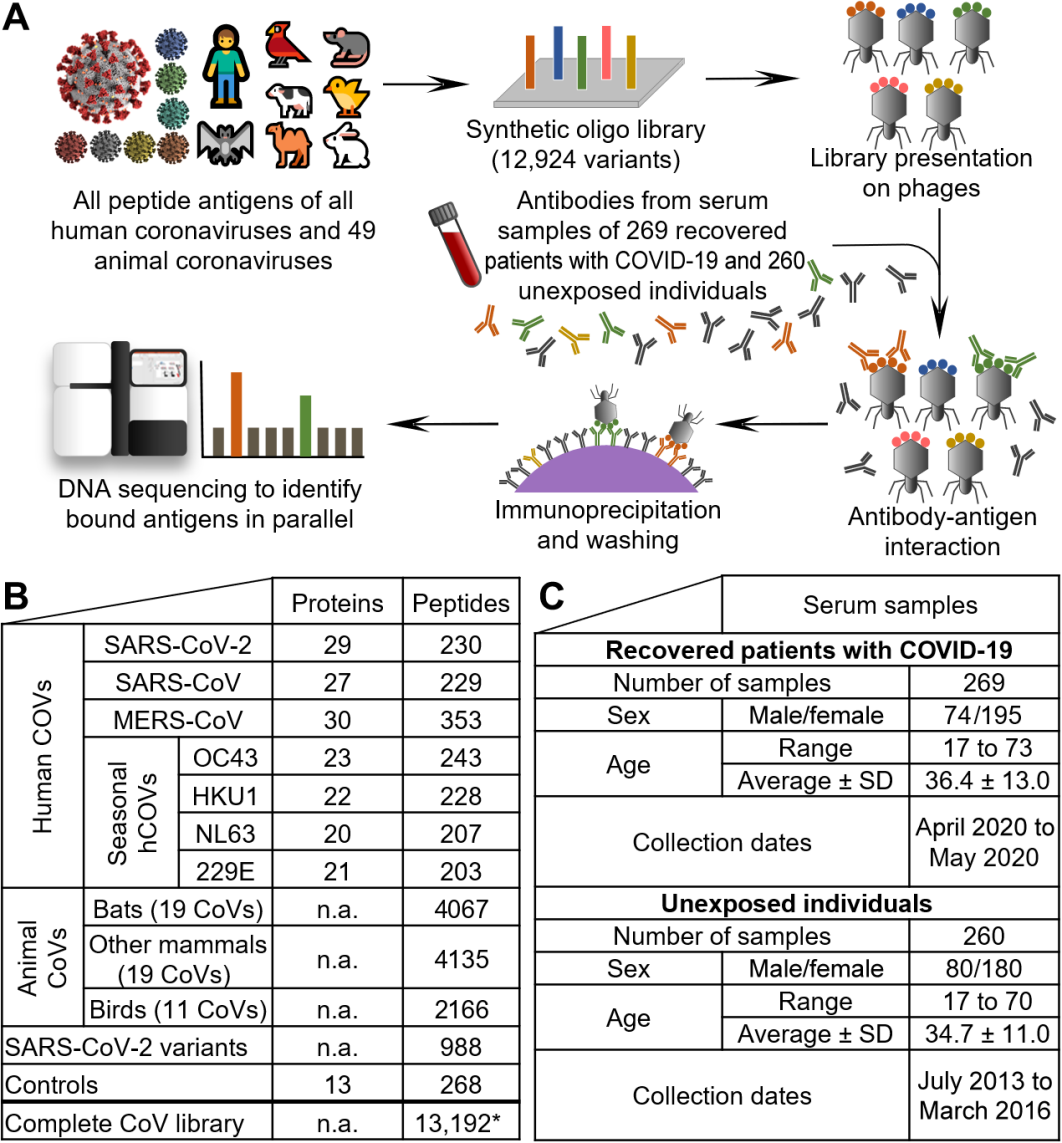
PhIP-Seq of CoVs directed antibody repertoires. A phage-displayed antigen library (**A**) of 12,924 hCoV and aCoV peptide antigens (**B**) was applied to serum samples of 260 unexposed individuals and 269 recovered patients with COVID-19 (**C**). The numbers of hCoV proteins per strain in (B) includes polyproteins being split into separate proteins. The listed MERS-CoV peptides also include the variant b CoV England 1. See data file S1 for a detailed list of all strains including accession numbers. The SARS-CoV-2 variants listed also include the reference SARS-CoV-2 peptides. The illustration of the SARS-CoV-2 virion is reproduced from CDC PHIL #23312 released as public domain (CDC/Alissa Eckert, MSMI; Dan Higgins, MAMS). Asterisk (*) indicates the number of unique peptides (a few are shared between groups). n.a., not applicable.

Assessing cross-reactivity against the animal coronavirome is challenging because of the large number of aCoV strains, necessitating immunological methods to probe for thousands of antigens in parallel. Antibody binding of antigens of SARS-CoV-2 is typically assessed by enzyme-linked immunosorbent assays (ELISAs) against full-length proteins/domains (*14*, *15*), by resolving crystal structures (*16*, *17*), or by peptide arrays (*18*, *19*). Pinpointing protein segments recognized by cross-reactive antibodies of all hCoVs and aCoVs requires high-resolution and high-throughput methods. Phage immunoprecipitation (IP) sequencing (PhIP-Seq) relies on the display of synthetic oligonucleotide libraries on T7 phages (*20*, *21*). Thereby, the displayed antigens can be rationally selected allowing hundreds of thousands of antigens to be probed in parallel. After mixing of the phage library with serum antibodies, unbound phages are washed away after IP, and enriched phages are detected by next-generation sequencing (Fig. 1A). PhIP-Seq has been adapted for assaying antibody binding against viruses [termed VirScan (*21*, *22*)] including SARS-CoV-2 and primarily other human hCoVs (*13*, *23*) as well as three bat CoVs (*13*). These studies have demonstrated suitability for diagnostic applications (*13*, *23*), as well as providing insights into cross-reactivity of hCoVs and COVID-19 severity (*13*). Limitations of PhIP-Seq (*20*) include length constraints of presented peptides (with short peptides inadequately representing conformational epitopes) and lack of eukaryotic posttranslational modifications (PTMs), which also affect the detectability of CoV antigens (discussed in detail in Discussion).

Here, we have generated a PhIP-Seq/VirScan library covering all 7 hCoVs and 49 aCoVs originating from diverse hosts including bats, rodents, domestic animals, and birds, represented as 12,924 peptides. We demonstrate that human serum antibody cross-reactivity extends beyond hCoVs to aCoVs and can be mediated by single mAbs. This pronounced cross-reactivity allows accurate detection of SARS-CoV-2 exposure without using any SARS-CoV-2 peptides and hence suggests diagnostic applications for the early stage of future pandemics caused by zoonotic spillovers of viruses to humans.

## RESULTS

### A library of 12,924 hCoV and aCoV peptides

We designed a PhIP-Seq library (experimental outline is shown in Fig. 1A) covering all open reading frames (ORFs) of hCoVs and aCoVs as 64–amino acid sections with 20–amino acid overlaps between adjacent peptides (Fig. 1B and data file S1). The sequences of 48 aCoVs were obtained from the National Center for Biotechnology Information (NCBI) reference genome (RefSeq, April 2020) database, and the sequence of another bat CoV related to SARS-CoV-2 (*24*) was included in addition. These strains broadly covered all groups of α, β, γ, and δ CoVs with their phylogeny illustrated in fig. S1. The CoV antigen library consisted in total of 12,924 peptides, with hCoVs representing ~20% of peptides and aCoVs representing ~80% of CoV antigens (Fig. 1B and data file S2). The 49 aCoVs contained 11 strains infecting birds, 19 strains infecting bats, and 19 more CoVs infecting other mammals such as rabbits, rodents, bovines, etc. (see data file S1 for a full list of aCoVs and hosts). For SARS-CoV-2, in addition to the reference genome, variants deposited in the NCBI database as of mid-April of 2020 were included. The antigen library also includes positive controls that confirmed detection of antibody responses against viruses previously reported to elicit population-wide immunity (*21*) and negative controls that did not show substantial binding (fig. S2).

**Fig. 2.**
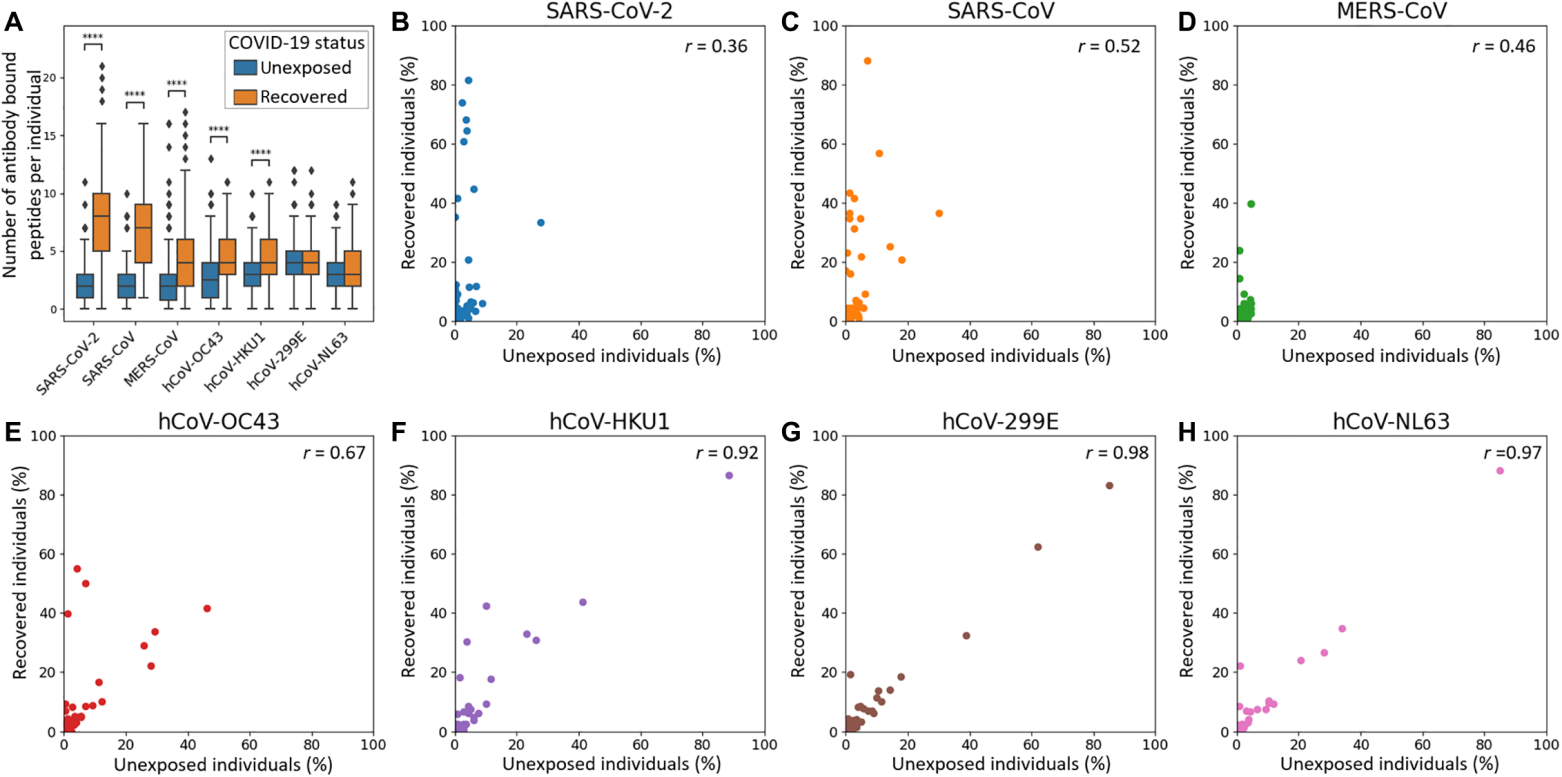
Detection of a high serum prevalence of seasonal hCoVs, interindividual variability of antibody repertoires against SARS-CoV-2, and cross-reactive antibody responses against seasonal hCoVs upon SARS-CoV-2 infection. (**A**) The numbers of antibody bound peptide antigens of hCoVs per individual are compared between unexposed individuals (*n* = 260) and recovered patients with COVID-19 (*n* = 269). For SARS-CoV-2, only peptides of the reference genome are included, whereas variants are not shown (listed in data file S3). The MERS-CoV peptides shown include also the variant b CoV England 1. The center line shows the median; box limits indicate the 25th and 75th percentiles as determined by Seaborn software; whiskers extend 1.5 times the interquartile range from the 25th and 75th percentiles, and outliers are represented by dots. Significance between the groups was calculated with the Mann-Whitney test (****P < 10^−4^; see fig. S5 for additional *P* value criteria). (**B** to **H**) Antibody responses in unexposed individuals and recovered patients with COVID-19 shown for each hCoV strain separately. Each dot represents a peptide with its abundance in the respective cohort plotted on the *x*/*y* axes. The correlation coefficient (Pearson *r*) between the groups of unexposed individuals and recovered individuals is displayed in the top right corner of each panel.

**Fig. 3.**
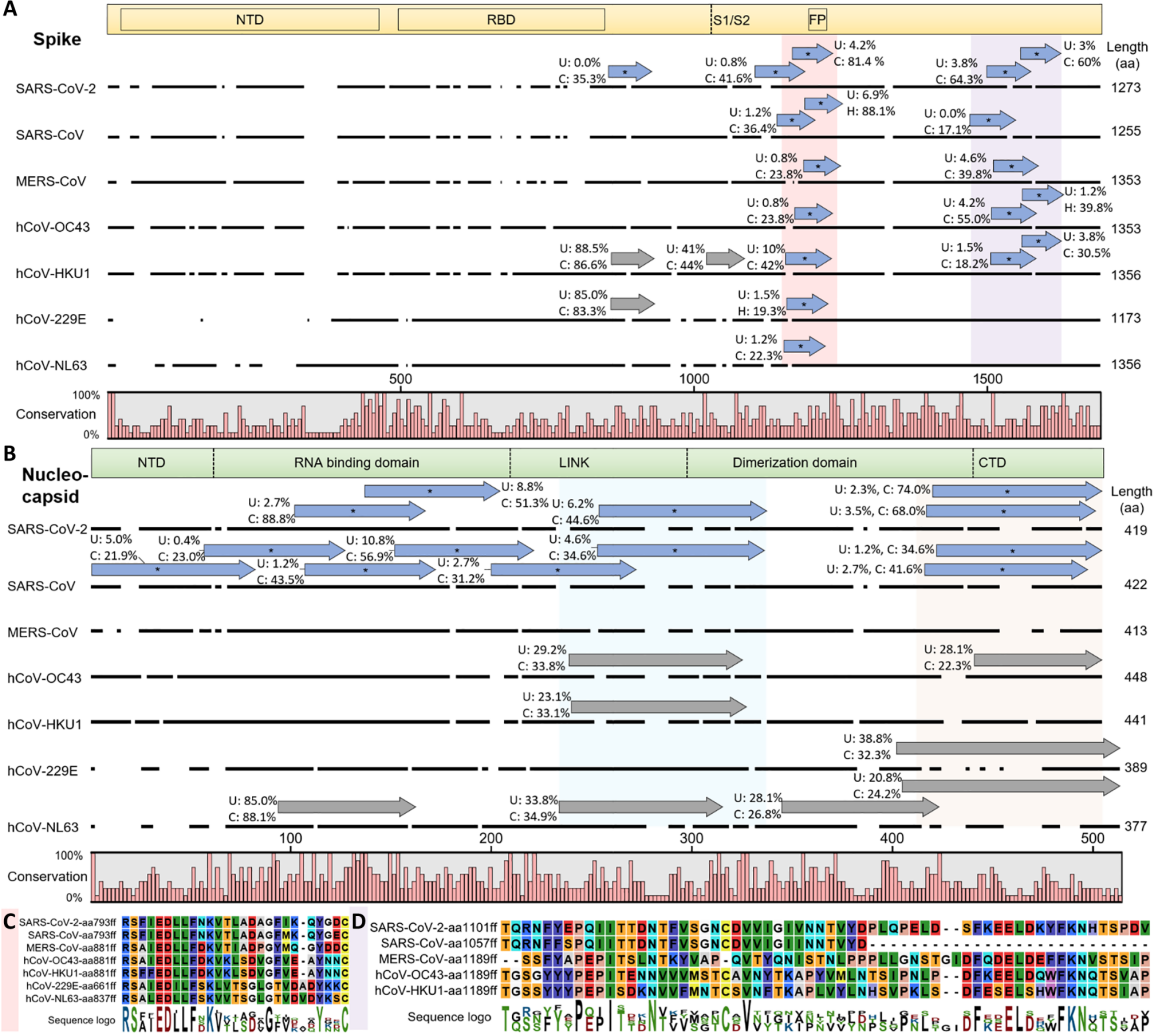
hCoV cross-reactive antibody responses. Cross-reactive and selective antibody binding of SARS-CoV-2 peptides and other hCoVs clusters in similar regions of the spike (**A**) and nucleocapsid (**B**) protein, with shared motifs of bound spike peptides highlighted (**C** and **D**). (A and B) Alignments of S and N proteins of all hCoVs. The dark line next to the strain identifier represents the protein sequence indicating gaps in the consensus alignment. Peptides bound at significantly different percentages (chi-squared/Kolmogorov-Smirnov tests and passing FDR correction; see data file S3) between unexposed (“U”) individuals or recovered patients with COVID-19 (“C”) are shown as blue arrows above the corresponding protein sequence and marked with an asterisk. The abundance of binding in U and C is indicated as percentages written next to the peptides. Gray arrows indicate similar recognition in >20% of unexposed individuals and patients with COVID-19. For SARS-CoV-2 only, peptides of the reference genome are included (variants not shown but listed in data file S3). The domain structure on top of each panel is based on SARS-CoV-2 S protein (*68*)/N protein (*69*), positions in other hCoVs shift along the alignment. Because of the different lengths of S and N proteins, the two panels are not drawn at the same scale. (C and D) Motifs from alignments of bound spike peptides in the regions marked in light red (C) and light purple (D) in (A). See fig. S3 (A and B) for full alignments of the peptides and details. Alignments of the nucleocapsid regions marked in light blue and light orange are due to space constraints shown in fig. S3 (C and D). aa, amino acid.

**Fig. 4.**
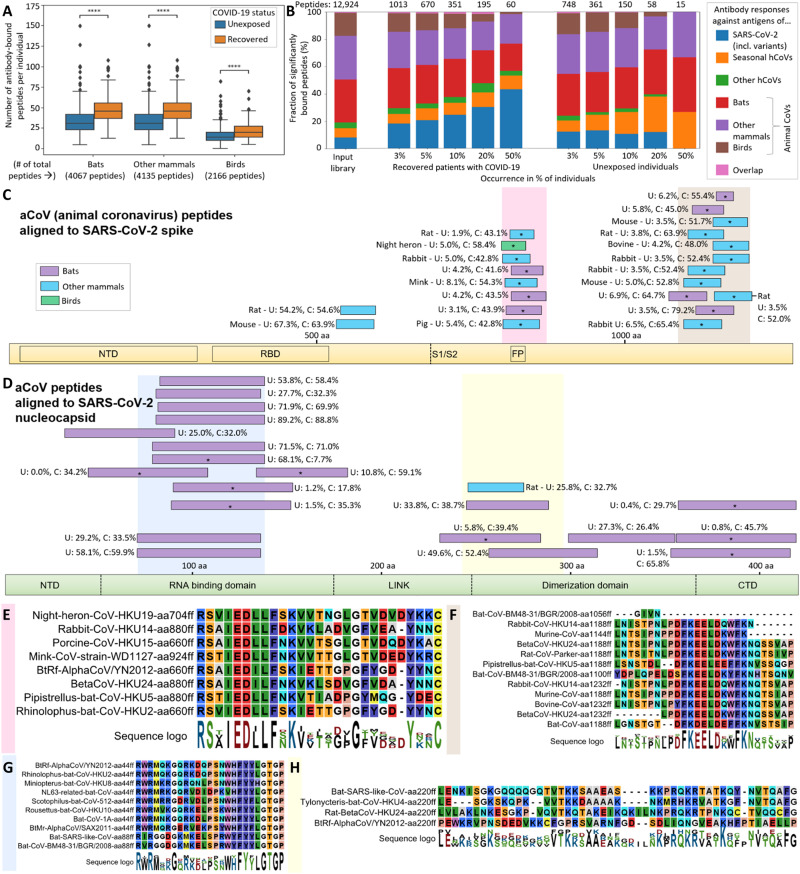
aCoV cross-reactive antibody responses. Cross-reactivity of human serum antibodies extends toward aCoVs in both SARS-CoV-2–recovered patients and unexposed individuals (**A** and **B**). Alignments of bound aCoV peptides to SARS-CoV-2 cluster in similar regions of the spike (**C**) and nucleocapsid (**D**) protein, with shared motifs of bound spike (**E** and **F**) and nucleocapsid (**G** and **H**) peptides highlighted. (A) Antibody responses against antigens of 49 aCoVs are summarized for the groups indicated (separate data for each strain are shown in fig. S5). In all panels of this figure, antibody binding data of the full set of 269 recovered patients with COVID-19 and 260 unexposed individuals are shown. “# of total peptides” refers to number peptides included for each strain within the library (Fig. 1B), whereas the number of antibody-bound peptides per individual is plotted for each group on *y* axis. The center line shows the median, box limits indicate the 25th and 75th percentiles as determined by Seaborn software, whiskers extend 1.5 times the interquartile range from the 25th and 75th percentiles, and outliers are represented by dots. Significance between the groups was calculated with the Mann-Whitney test (****P < 10^−4^; see fig. S5 for additional *P* value criteria). (B) Ratios of hCoVs and aCoVs antigens bound at different frequencies in recovered patients with COVID-19 and unexposed individuals. The input library is shown as control representing the library content before testing for antibody binding. The numbers on top of the panel indicate the absolute number of peptides per group (as each bar shows a relative distribution between the groups). “Other hCoVs” includes SARS-CoV and MERS-CoV. “Overlap” refers to a few identical peptides of multiple strains that cannot be assigned to a single group. (C and D) Alignments of S and N peptides of aCoVs to SARS-CoV-2 S protein (C) and N protein (D). The peptides were aligned using the BLAST algorithm in standard parameters, and only aligned regions of the peptides are shown. Peptides bound at significantly different percentages (chi-squared/Kolmogorov-Smirnov test and passing FDR correction; see data file S3) between unexposed (U) individuals or recovered patients with COVID-19 (C) shown are marked with an asterisk. The abundance of binding in U and C is indicated as percentages written next to the peptides. Nonsignificantly scored peptides are only shown if being bound in >20% of unexposed individuals and patients with COVID-19. Because of the large number of significantly bound aCoV S peptides, only peptides being bound in >40% of recovered individuals are shown, whereas a full list is provided in data file S3 (including BLAST alignments). The S/N protein domain structure at the bottom of each panel as outlined in Fig. 3. Because of the different lengths of S and N proteins, the two panels are not drawn at the same scale. (E to H) Motifs from alignments of bound aCoV S peptides in the regions marked in light red (E) and light brown (F) in (C) and bound aCoV N peptides in the regions marked in light blue (G) and light yellow (H) in (D). See fig. S7 for full alignments of the peptides and details.

We tested immunoglobulin G (IgG) antibody binding against this CoV library with 260 prepandemic serum samples of individuals unexposed to SARS-CoV-2 that had been collected in 2013 to 2016 (Fig. 1C) (*25*, *26*). These antibody repertoires were compared with 269 samples of recovered patients with COVID-19 obtained in April and May 2020. The serum samples were mixed individually with the phage library displaying the CoV antigens (Fig. 1A). Phages bound by antibodies were immunoprecipitated, and unbound phages were washed away. The bound phages were polymerase chain reaction (PCR)–amplified and sequenced. Thereby, we obtained for each library variant in each sample a read count after IP. These read counts were compared with the “input” read counts of the phage material before mixing with serum samples. We used a generalized Poisson distribution approach previously reported (*27*) to calculate *P* values for significance of the enrichment of each library variant in each sample. These *P* values were filtered in each sample by strict Bonferroni correction (<0.05) to counteract the problem of multiple hypothesis testing (see Materials and Methods for details). In total, we have assayed for about 5 million antibody-peptide interactions (12,924 hCoV peptides in each of the 529 individuals). On average of 114 CoV peptides were significantly bound per unexposed individual and 189 CoV peptides per recovered patient with COVID-19. Most analyses were based on antibody responses against 579 CoV peptides from different CoV strains and proteins, shared by more than 5% of either group (data file S3). Of these, 190 peptides showed significantly different abundances between the groups (data file S3). Bound antigens included peptides originating from hCoVs (Figs. 2 and 3) and aCoVs (Fig. 4), which are discussed sequentially in the following sections.

### Antibody repertoires against hCoVs and cross-reactivities

As expected, sera of recovered patients with COVID-19 bound significantly more peptides of SARS-CoV-2 than sera of unexposed individuals (Fig. 2A). In addition, many peptides of SARS-CoV, MERS-CoV, and the seasonal hCoVs OC43 and HKU1 were significantly more frequently bound in recovered patients than unexposed individuals, in line with cross-reactivity previously reported (*13*).

Unexposed individuals showed abundant antibody responses against all seasonal hCoVs (Fig. 1, E to H): Peptides of hCoV-NL63 were significantly bound in up to 88% of unexposed individuals, peptides of hCoV-HKU1 in up to 87%, peptides of hCoV-229E in up to 83%, and peptides of hCoV-OC43 in up to 46% (Fig. 1, E to H, and data file S2). The same peptides were bound at similar frequencies in recovered patients with COVID-19 and originated mostly from spike (S) or nucleocapsid (N) proteins (Fig. 3 and data file S3) with these epitope-resolved results being in agreement with previous studies on the seroprevalence of seasonal hCoVs using ELISAs (*28*).

Sera of recovered patients with COVID-19 showed an overrepresentation of several peptides of SARS-CoV-2 that showed no binding or binding at very low percentages in unexposed individuals (Fig. 3B). Twelve peptides passed FDR [Benjamini-Hochberg false discovery rate (*29*), a method to correct for multiple hypothesis testing] correction for being significantly different between the two groups of individuals (for the SARS-CoV-2 reference genome, additional peptides of SARS-CoV-2 variants passed significance thresholds and are listed in data file S3). Although nearly all patients with COVID-19 showed binding against at least one peptide in S or N proteins and some peptides being bound in up to 81.4% of recovered patients (Fig. 1B), no convergence of antibody responses against the same peptide were detected in all individuals (which is limited to the interactions detectable with this PhIP-Seq library; see Discussion). This finding differs from near universal recognition of some viral epitopes previously observed by PhIP-Seq/VirScan for other human viruses (*12*) and replicated with controls in this study (fig. S2), suggesting that the antibody response against SARS-CoV-2 can exhibit substantial interindividual variability.

COVID-19 serum samples also showed common binding against SARS-CoV (Fig. 2C), to which it is unlikely that these individuals had been exposed, indicating detection of cross-reactivity of antibodies targeting SARS-CoV-2 (*14*, *15*). Three SARS-CoV spike peptides had significantly enriched binding in up to 88.1% of recovered individuals with COVID-19 compared with up to 6.9% of unexposed individuals (Fig. 3C and data file S3). A nonstructural protein (NSP2) of SARS-CoV was even bound in 30% of unexposed individuals and 36% of recovered individuals, possibly owing to higher conservation of such NSPs underlying less selective pressure than S and N proteins mostly responsible for infectivity and targeted by neutralizing immune responses. In addition, three peptides from the MERS-CoV (Fig. 3D) S protein were differentially enriched in the COVID-19 recovered cohort, passing FDR correction for significance of this difference (Fig. 3A and data file S3).

Cross-reactive responses from SARS-CoV-2 also extended to the spike proteins of all four seasonal hCoVs (Fig. 3A) related to specific motifs (Fig. 3, C and D). Three peptides from each hCoV-OC43 and hCoV-HKU1 were significantly differentially bound between unexposed and recovered individuals. One of these peptides arose from similar regions of the S protein [fusion peptide (FP) site; Fig. 3A], and hCoV-229E and hCoV-NL63 peptides around this position were significantly differentially bound. Such cross-reactivity has been primarily reported for hCoV-OC43 (*13*). Our data suggest that cross-reactivity arising from SARS-CoV-2 infection targets a similar motif in all human hCoVs (RSXIEDLLFXK; Fig. 3C and see fig. S3A for an alignment of the complete bound peptides and fig. S4 illustrating that the motif is exposed on the surface of SARS-CoV-2). Toward the C terminus of the spike protein, another region was bound at significantly different frequencies between recovered and unexposed individuals in SARS-CoV-2, SARS-CoV, MERS-CoV, hCoV-OC43, and hCoV-HKU1 (Fig. 3A). This longer region appeared to contain two distinct motifs (Fig. 3D and figs. S3B and S4 illustrating surface exposure on the SARS-CoV-2 spike protein). In the nucleocapsid protein, less clear motifs were apparent in regions bound by antibodies at similar levels between hCoVs (Fig. 3B and fig. S3, C and D) potentially owing to separate binding of smaller motifs by different antibodies (which may explain why some of the peptides were bound at different levels and others were not; Fig. 3B).

### Antibody cross-reactivity extends toward aCoVs

In addition to antibody binding to hCoV peptides, we also detected dozens of aCoV peptides significantly bound per individual (Fig. 4A). These peptides originated from aCoVs with diverse hosts including all three major groups: bats, other mammals (such as rodents), and birds. Sera of recovered patients with COVID-19 bound significantly more aCoV peptides than unexposed individuals when scoring these groups (Fig. 4A). Scoring differences on a strain level (fig. S5) showed varying antibody responses, as observed for hCoVs (Fig. 2A): Peptides of some bat CoVs closely related to SARS-CoV-2 (fig. S1) were highly significant for being more frequently bound in recovered patients with COVID-19 than in unexposed individuals (*P* < 10^−4^). In contrast, peptides of bat CoVs such as a NL63-related strain were not bound to a different extent between the two groups (*P* > 0.05; fig. S5). This finding is in agreement with the seasonal hCoV-NL63 also not exhibiting a significant difference (Fig. 1A). Similarly, for aCoVs of birds or other mammalian hosts, we observed some strains being bound by antibodies at similar levels in unexposed and recovered patients with COVID-19 and other strains being bound differentially. For example, aCoVs from rabbit, mouse rat, pig, cow, and the bird night heron showed highly significant differences between the two groups, whereas aCoVs from felines, ferrets, and camel did not show significant differences (fig. S5). We also analyzed differences in antibody binding based on genetic differences of the aCoVs (fig. S6). These results suggest that antibody responses against b aCoVs show the greatest statistical difference between unexposed and recovered individuals (as expected from their closer relationship to the b hCoV SARS-CoV-2). Differential responses were not limited to b aCoVs with some antigens of a, g, and d aCoVs also being significantly more frequently bound by antibodies in recovered patients with COVID-19 than unexposed individuals (fig. S6).

Relating these antibody responses to hCoVs, aCoV peptides amounted for about 80% of the initial library content, whereas hCoV peptides represented about 20% (Figs. 1B and 4B). In recovered patients with COVID-19, antibody responses against SARS-CoV-2 were overrepresented, especially regarding peptides bound in larger fractions of the cohort (Fig. 4B). In unexposed individuals, aCoV peptides took up a larger fraction of bound peptides with seasonal hCoVs still being overrepresented. These results suggest that peptides of hCoVs are dominantly bound by human antibodies and aCoVs contribute to the detected antibody repertoire by cross-reactivity.

To pinpoint the potential cross-reactivity underlying the binding signal within aCoVs, we mapped the bound aCoV peptides to the SARS-CoV-2 proteome (summarized in Fig. 4, C and D, see data file S3 for alignment data for all peptides). Two clusters in the S protein near the FP site and the C terminus were apparent (Fig. 4C and fig. S7, A and B), which are similar to the cross-reactive regions observed in hCoVs [Fig. 3, A, C, and D; (*13*), accessible on the surface of the S protein; and fig. S4]. Near the FP site, the RSXIEDLLFXK motif observed from hCoVs (Fig. 3C) appeared in near identical form in aCoVs (Fig. 4E). The N-terminal cluster yielded for aCoVs even a clearer motif (FKEELDXXFKN; Fig. 4F) than the same region in hCoVs (Fig. 3D), possibly owing to the larger number of peptides aligned. In the N protein, bound aCoV peptides clustered mostly in the RNA binding domain (Fig. 4D), exhibiting also a shared motif (Fig. 4G and see fig. S7, C and D, for full alignments). Although similar peptides in the RNA binding domain of the N protein of hCoVs are bound (Fig. 3C), the exact motif identified in aCoVs (Fig. 4G) was not apparent. Furthermore, a C-terminal motif found in aCoVs (Fig. 4G) did not have a direct equivalent in hCoVs (Fig. 3B). The two main spike motifs detected from aCoV peptides (Fig. 4, E and F) also match sequences previously reported in a different cohort (*13*), suggesting that human antibodies raised upon SARS-CoV-2 infection can also cross-react with aCoVs. In addition, cross-reactivity of human antibodies against aCoVs appears to also arise from seasonal hCoVs, because some S and N peptides are bound at similar levels in unexposed controls and recovered patients with COVID-19. These include rat/mouse CoV peptides around amino acid 600 in the S protein (Fig. 4C) or bat peptides around amino acid 100 in the N protein (Fig. 4D).

### Patient-derived immunoglobulins with cross-reactivity potential

**Fig. 5.**
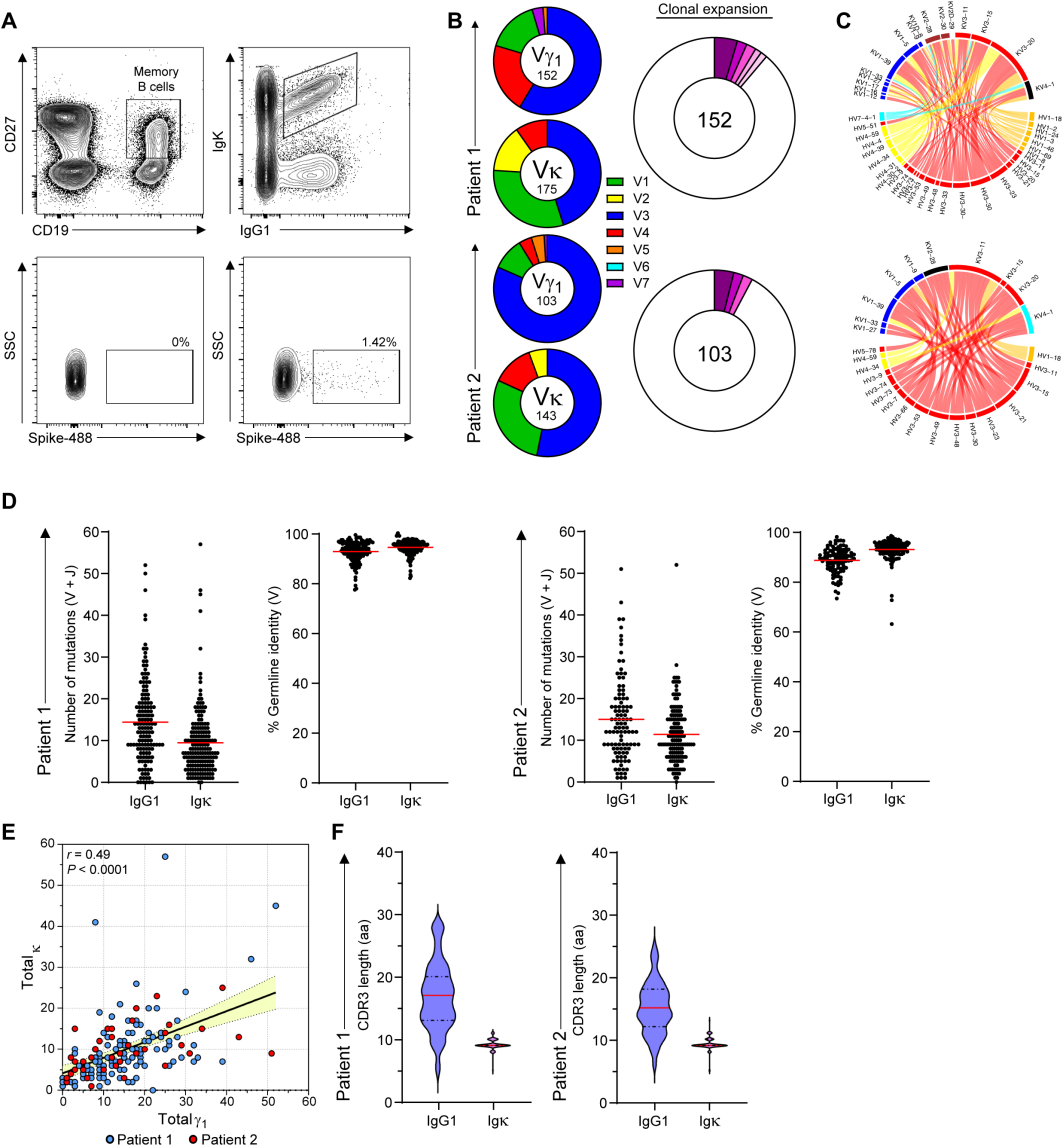
Anti SARS-CoV-2 antibodies by single B cell immunoglobulin sequencing. (**A**) Gating strategy for spike-specific memory B cells sorting (CD19^+^, CC27^+^, IgG1^+^, Igκ^+^, and spike binding). SSC, side scatter. (**B**) Pie charts depicting the distribution of V (variable) genes in Ighγ1, top and Igκ of spike-specific B cells (left) and clonal expansion of spike reactive B cells (right). Each colored slice represents a unique clone. Singleton sequences are shown in white. (**C**) Circos plots showing coupled heavy- and light-chain sequences of the sorted cells. (**D**) The number of mutations identified in the V and J genes of heavy and light immunoglobulin chains and the percent germline identity of their V genes. (**E**) Correlation between the mutational load in the Ighγ1 versus Igκ chains. Each dot represents a sequenced antibody heavy or light chain. Pearson’s correlation test was used for *r* and *P* value. (**F**) CDR3 amino acid lengths of Ighγ1 and Igκ.

Although we observed pronounced antibody cross-reactivity between SARS-CoV-2 and hCoVs and aCoVs (Figs. 2 to 4), the underlying mechanisms are unclear. Cross-reactivity is most likely to arise from polyclonal antibodies; however, the occurrence of shared motifs (Figs. 3, C and D, and 4, E to H) suggests potential cross-reactive recognition by a single antibody. To examine this possibility, we sequenced B cell immunoglobulin genes derived from recovered patients and generated mAbs (*30*) for testing in PhIP-Seq. Because cross-reactive binding to the receptor binding domain (RBD) is less likely to occur, we sequenced patient-derived B cells that show reactivity with the intact spike trimer that carries more conserved domains with cross-reactive potential. Spike-specific single memory B cells (CD19^+^, CC27^+^, IgG1^+^, and Igκ^+^) were sorted from peripheral blood mononuclear cells of convalescent patients and subjected to PCR amplification of their immunoglobulin genes, followed by gene sequencing (Fig. 5A). Analysis of the emerging immunoglobulin sequences revealed significant enrichment for VH3 and VK3, and these types of V genes were the most abundant paired chains (Fig. 5, B and C). On the basis of the variable region sequences, we clustered the clones to determine the clonal expansion of spike-specific memory B cells. This analysis revealed that most of the spike-binding memory B cells were not significantly expanded, and most recovered immunoglobulin sequences appeared only once (Fig. 5B). The average number of somatic mutations per cell was 16 and 10 for the heavy and light chains, respectively (Fig. 5D), which is slightly higher than the average mutation load of memory cells in healthy individuals (*31*). As expected, the number of mutations in the heavy chains was in correlation to the mutation load in the light chains (Fig. 5E), and the CDR3 (complementarity-determining region 3) lengths of the light and heavy chains (Fig. 5F) were similar to typical immunoglobulins in naive B cells (*31*). Comparing our findings with previous published data shows that the frequency of mutations was slightly higher in our cohort; however, the observed clonal expansion was lower (table S1). Most studies focused on the RBD target, whereas we used the full spike trimer as a bait that carries multiple domains. We conclude that although robust clonal expansion was not detected in recovered patients, some of the mutated spike-specific memory cells are the product of antibody affinity maturation and may have broad binding reactivity.

### Pan-specific mAbs can mediate cross-reactivity against hCoVs and aCoVs

**Fig. 6.**
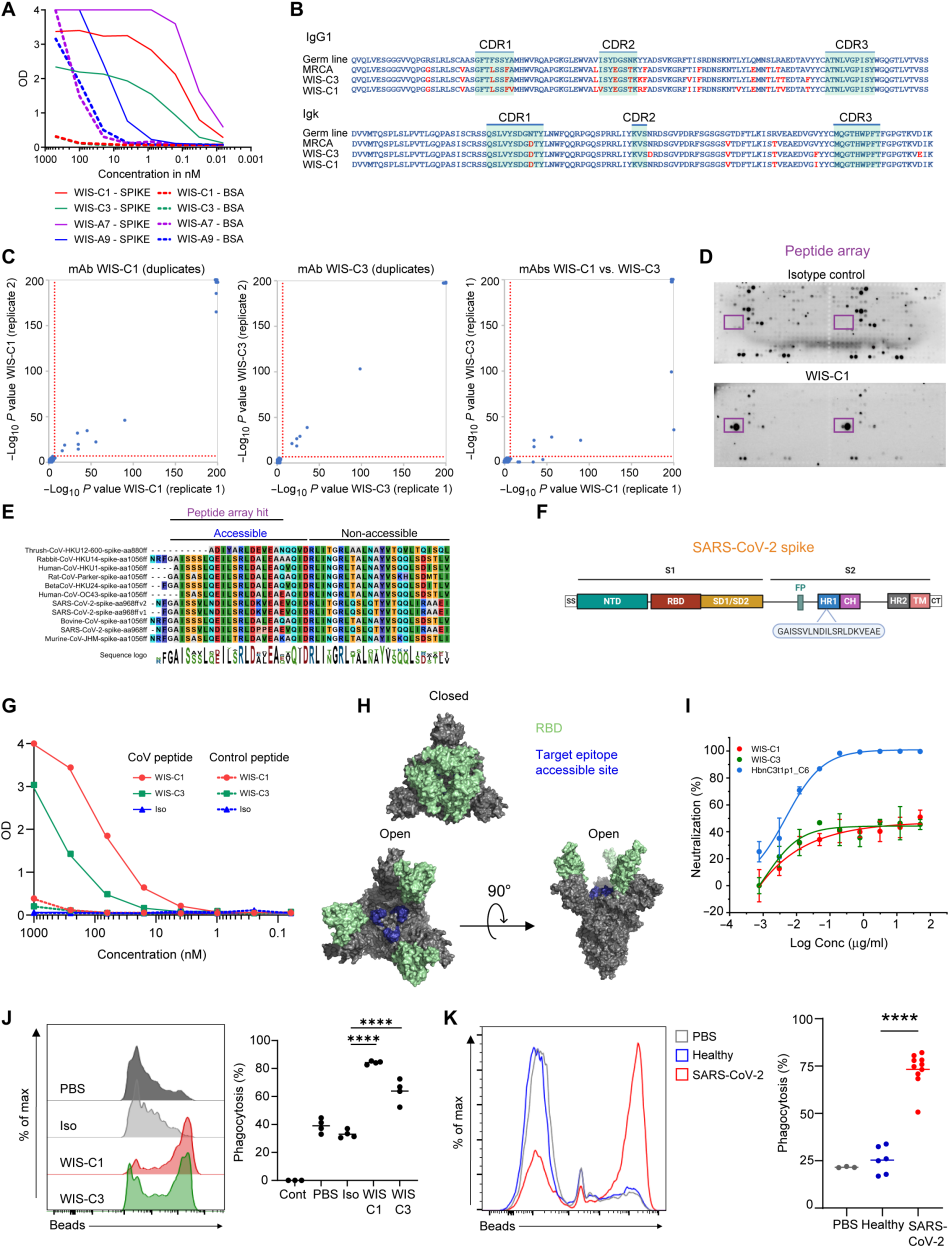
Patient-derived, pan-specific mAbs. (**A**) mAb reactivity to spike protein and BSA at different concentrations by ELISA. (**B**) Comparison between amino acid sequences of WIS-C1 and WIS-C3 to their germ line and most recent common ancestor (MRCA) configurations. Mutated amino acids appear in red. (**C**) PhIP-Seq–based identification of targeted peptides within the CoV-antigen library of WIS-C1 (left), WIS-C3 (middle), and comparison of the peptides bound by the two antibodies (right). The two mAbs were mixed with the phage displayed antigen library and processed in the same way as serum samples. Reaction mixtures were set up in duplicates and significantly bound peptides are marked (threshold indicated by dotted red line). The maximum *P* values computed are cut off at −log_10_ 200. See fig. S8 for a list of significantly bound peptides. (**D**) Epitope mapping using spike peptide array of isotype control antibody (top) and the WIS-C1 antibody (bottom). WIS-C1 epitope target is shown in the purple rectangle. (**E**) Motif analysis of the target epitope of WIS-C1 and WIS-C3, obtained by multiple sequence alignment of the top 6 hCoVs and top 9 aCoVs PhIP-Seq hits. See fig. S8 for full alignments of the peptides and details. (**F**) Linear SARS-CoV-2 spike schematics depicting the location of the target epitope on the viral protein. (**G**) Binding of WIS-C1 and WIS-C3 to the target or control peptide by ELISA. (**H**) WIS-C1 and WIS-C3 target epitope projected on the crystal structure of SARS-CoV-2 spike in the closed (top) and open (bottom) confirmations. (**I**) Neutralization activity of pseudo-viruses by WIS-C1 and WIS-C3 compared with HbnC3t1p1_C6, as a positive control. Representative of two independent experiments; error bars indicate SDs of technical repeats. (**J** and **K**) Fluorescence-activated cell sorting plots depicting the uptake of fluorescent spike-coated spheres by THP-1 monocytes in the presence of (J) mAbs or (K) patient sera. (J) Four independent experiments; ****P < 0.0001, one-way analysis of variance or (K) a representative of two independent experiments; ****P < 0.0001, two-tailed Student’s *t* test. OD, optical density.

To test the patient-derived immunoglobulins in PhIP-Seq, we cloned the recovered sequences into expression vectors and produced mAbs (*30*). Previous studies found that most of the SARS-CoV-2 –neutralizing antibodies are very similar to their germline configuration (*32*–*35*). In contrast, broadly reactive antibodies generated during chronic infection, such as in HIV-infected patients, are highly mutated (*36*). Furthermore, if SARS-CoV-2 activates preexisting cross-reactive memory cells that were raised against other coronaviruses, then the emerging antibodies are expected to carry more mutations than those elicited in a primary response. Therefore, to maximize the probability for detection of broadly reactive mAbs, two highly mutated antibodies were chosen for expression as IgG1 (*30*), followed by spike-binding and PhIP-Seq analyses. In parallel, we also examined additional two antibodies that carried very few somatic mutations, WIS-A7 and WIS-A9 for cross-reactivity comparison. ELISA revealed four antibodies that showed detectable spike-binding activity (Fig. 6A). Among these antibodies, C1 and C3 mAbs carried a significant load of mutations and shared 88.7 to 90.1% VH sequence identity (Fig. 6B). Using our PhIP-Seq assay, we found that both C1 and C3 bound significantly to several peptides of hCoVs (SARS-CoV-2, SARS-CoV, hCoV-OC43, and hCoV-HKU1) and several aCoVs [including bovine, rodent, thrush (bird), rabbit, and bat as hosts] spike proteins found within our phage display library (Fig. 6C, left and middle, and see fig. S8 for a full list of peptides). In contrast, a control mAb did not show any significantly enriched peptides. C1 and C3 showed nearly identical binding to the same peptides, with slight differences for two peptides (Fig. 6C, right), suggesting that the additional mutations of C3 do not strongly affect cross-reactivity against our CoV antigen library. Consistently, a part of the SARS-CoV-2 peptide target was detected in a spike peptide array binding assay (Fig. 6, D and E). The sequence of the binding site was very similar among the different strains of coronaviruses and was composed of a consensus motif that is located in the S2 domain of the SARS-CoV-2 spike protein (Fig. 6F). This motif lies in a region different from frequently bound peptides identified in hCoVs (Fig. 3A) and aCoVs (Fig. 4C). In agreement with these results, the mAbs also bound the target peptide in ELISA (Fig. 6G). Because, structurally, a part of this peptide sequence is not exposed on the spike trimer surface, it is most likely that the mAbs only bind the N terminus of the peptide (Fig. 6, E and H). A7 mAb bound a peptide that was derived from the SARS-CoV-2 spike protein and a very similar peptide that is part of a bat SARS-like aCoV spike protein (fig. S9A). This region of the SARS-CoV-2 spike protein (starting at amino acid 748) was a binding target in 35.3% of our recovered COVID-19 cohort, but not in our healthy control cohort (Fig. 3A). Although A9 was found to bind the spike protein in ELISA, our PhIP-Seq assay did not identify a linear binding target (fig. S9B). This contradiction can be a result of A9 binding to a nonlinear epitope or PTMs that are inadequately represented within our phage library (see also Discussion below). Overall, for the highly mutated antibodies showed significant cross-reactivity, providing a proof-of-concept evidence for the existence that a single antibody can bind multiple coronaviruses. However, elucidating the frequency of single cross-reactive antibodies in the general population will require further studies that involves before and after exposure to SARS-CoV-2 analyses.

To examine whether cross-reactive mAbs support protective functional activity, we examined their capacity to neutralize pseudo-virus that carried the SARS-CoV-2 spike protein. Both the C1 and C3 displayed poor neutralization activity compared with a control antibody that was previously described (Fig. 6I) (*35*). Typically, IgG1 antibodies support non-neutralizing activity as well; however, because C1 and C3 mAbs bind an internal epitope that is exposed only in the open conformation of the spike trimer, they might not be able to support these functions. Antibody-dependent cellular phagocytosis (ADCP) is a process wherein immune cells uptake a target through antibody-mediated interaction with Fc receptors (*37*, *38*). To examine ADCP activity, we produced immune complexes by incubating spike-coated beads with mAbs and tested the ability of THP-1 monocytes to uptake these targets. Both C1 and C3 mAbs promoted the uptake of spike-coated beads by THP-1 cells, whereas the control antibody did not contribute to this process (Fig. 6J). To validate whether serum antibodies can support ADCP, we examined whether patient-derived serum can promote phagocytic activity. We found that all of the patients have antibodies in their serum that can support ADCP (Fig. 6K). This antibody-mediated activity can be supported by few or multiple neutralizing and non-neutralizing antibodies. Collectively, we conclude that cross-reactive mAbs derived from patients with COVID-19 show functional activity.

To examine whether the mAbs were subjected to affinity maturation, we examined whether amino acid replacements in the complementarity-determining regions and in the framework regions of the V regions occurred using the Baseline tool (*39*, *40*). Similar to other antigen-experienced B cells, the mutation patterns in these sequences indicate that the B cells producing these IgGs experienced statistically significant negative selection in the framework region in both the heavy and light chains. As for the CDRs, the selection estimations are not significant (not negative and not positive in neither chain) (fig. S11). This observation is typical when mAb selection is estimated on the basis of a few sequences. These data indicate that according to the selection patterns, it seems that these cells were subjected to typical antigen-experienced maturation process that involved negative (purifying) selection in the framework regions and non-negative in the complementary determining regions.

### Animal coronavirome–based high-throughput diagnostics

**Fig. 7.**
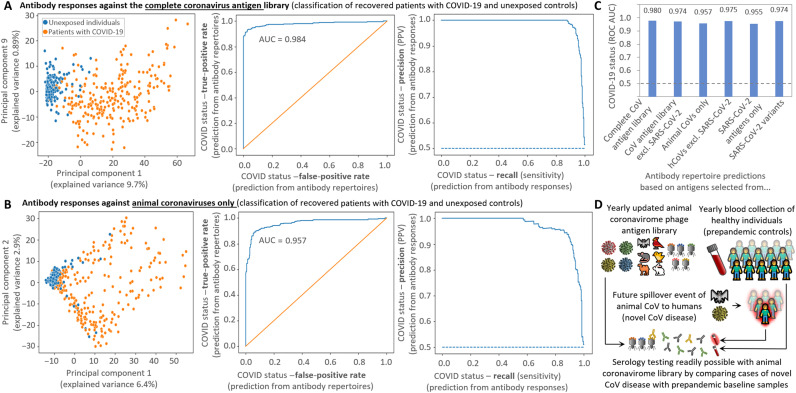
Machine learning based diagnostics. Antibody responses against antigens of aCoVs allow accurate identification of recovered patients with COVID-19 (**A** to **C**) suggesting a possible diagnostic strategy for the early stage of future pandemics (**D**). (A and B) Classification of recovered patients with COVID-19 (*n* = 269) and unexposed controls (*n* = 260) from antibody responses against all antigens of the CoV library (A) and only from the animal coronavirome (B). PCA on fold changes of antibody responses separates between hCoV antibody responses of unexposed individuals and recovered patients with COVID-19 (left) and a machine learning predictor accurately identifies infected individuals illustrated by receiver operating characteristic (ROC) curve (middle) and PR curve (right). (C) AUC of the ROC curve of predictions with subgroups of antigens of the CoV antigen library. All predictions [including (A) and (B)] were performed with gradient boosting decision trees [XGBoost classifier (*70*)] with leave-one-out cross-validation from CoV peptides bound in >5% individuals. The aCoVs used for these predictions are limited to RefSeq strains (deposited in 2018), whereas a bat CoV related to SARS-CoV-2 (*24*) was excluded (data file S1). (D) Creating regularly updated antigen libraries representing the animal coronavirome alongside the collection of baseline samples of healthy individuals can provide the basis for a serological assay to readily respond in case of a novel zoonotic transmission event.

Testing for multiple CoV antigens at high resolution in parallel could yield higher specificity than conventional tests based on single proteins of SARS-CoV-2 by improving discrimination from seasonal hCoVs. Most current SARS-CoV-2 serological tests rely on detection of entire S/RBD or N proteins, reporting an aggregate of binding against all epitopes within (*10*, *15*). PhIP-Seq implementations of CoV antigen libraries primarily based on hCoVs have also demonstrated highly accurate diagnostic performance (*13*, *23*). We set out to benchmark our extensive CoV antigen library containing both hCoVs and a large set of aCoVs for diagnostic applications. Interpreting the epitope-resolved antibody binding data reported by our assay extends beyond conventional serological tests, as binding against various epitopes of all hCoV needs to be weighed. We used gradient boosting trees (XGBoost) to build a predictor that accurately separates patients with COVID-19 from healthy controls based on antibody signatures (Fig. 7 and fig. S10) when trained on antibody binding data of the complete library [area under the curve (AUC) = 0.980; Fig. 7A]. Depending on the intended application and cutoffs used, this assay displays with this set of samples 100% specificity at 87% sensitivity (reporting virtually no false positives for this set of samples) or 92% specificity at 95% sensitivity [Fig. 7A; precision-recall (PR) curve]. Even the subset of antibody binding data solely against peptides of aCoVs allowed accurate separation between exposed and unexposed individuals (AUC = 0.96; Fig. 7B).

When looking at the principal components analysis (PCA; Fig. 7, A and B) performed on the log fold change (number of reads of bound peptides versus baseline sequencing of phages not undergoing IP) of significantly enriched peptides, unexposed individuals clustered together, whereas samples of recovered patients with COVID-19 showed a greater spread. This illustrates the interindividual variability in hCoV antibody responses elicited by SARS-CoV-2 and the PCA separation based on the entire CoV antigen library (Fig. 7A) or the subset of aCoV antigens (Fig. 7B) yielded similar results. This interindividual variability in recovered patients with COVID-19 may represent differences in immune responses or could also be affected by the time passed between infection and blood sampling [although this period is <3 months for our COVID-19 cohort where anti–SARS-CoV-2 antibody responses may show relatively high stability (*41*)].

As outlined above, aCoVs alone are enough to accurately separate between exposed and unexposed individuals. When further adding other hCoVs except SARS-CoV-2 to the aCoVs (Fig. 7C), the accuracy slightly increased to nearly the same level as using the complete library including SARS-CoV-2 antigens (AUC = 0.97; Fig. 7A). In addition, binding data against hCoVs peptides excluding SARS-CoV-2 allowed for accurate detection of past infection (Fig. 7C). SARS-CoV-2 antigens alone yielded lower predictive power, but including also peptides originating of SARS-CoV-2 variants improved the AUC (Fig. 7C). This difference is possibly owing to the low redundancy in the included SARS-CoV-2 peptides of the reference strain, which improves when adding slightly different variants (that potentially do not strongly affect antibody binding). In this respect, including identical SARS-CoV-2 reference peptides in the input library could also be a strategy to improve the performance of diagnostic PhIP-Seq assays. However, given the already large redundancy arising from non–SARS-CoV-2 hCoVs and aCoVs in our existing library, it does not appear necessary to also add duplicates of peptides from these groups. Together, these results indicate that it is possible to accurately detect SARS-CoV-2 exposure, without including actual SARS-CoV-2 antigens, suggesting a potential diagnostic strategy for the early stage of future pandemics.

## DISCUSSION

Overall, our data indicate that antibody cross-reactivity observed for hCoVs (*12*, *13*) extends to aCoVs and PhIP-Seq/VirScan is a feasible strategy to capture cross-reactive antibody responses at an epitope-resolved resolution. Peptides bound in a larger fraction of individuals typically featured similar motifs and isolation of human mAbs confirmed binding of single antibodies to multiple hCoV and aCoV peptides bearing a similar motif. Cross-reactivities against aCoVs observed in our study can be assigned to at least two causes: (i) Cross-reactivity in individuals unexposed to SARS-CoV-2, possibly owing to exposure with seasonal hCoVs. These responses included peptides of rodent CoVs bound in up to 67% of unexposed individuals mapping to the SARS-CoV-2 S protein (around amino acid 600; Fig. 4C) and bat and rat peptides mapping to the SARS-CoV-2 N protein (bound in up to 89% of unexposed individuals; Fig. 4D). (ii) Cross-reactivity toward hCoVs and aCoVs caused by SARS-CoV-2 infection. Such cross-reactivity has previously been reported against seasonal hCoVs and three bat CoVs closely related to SARS-CoV-2 (*13*). Our data indicate that cross-reactivity induced by SARS-CoV-2 infection extends beyond bat CoVs, also including peptides of aCoVs from diverse animals as rodents, rabbit, mink, porcine, bovine, and birds (e.g., night heron) as natural host (among others; data file S3).

A potential third aspect of cross-reactivity would be represented by preexisting immunity against seasonal hCoVs protecting against SARS-CoV-2 infection (*12*) (or even providing protection against potential future spillovers of aCoVs to hCoVs). hCoV or aCoV peptides bound at higher abundance in unexposed individuals than recovered patients with COVID-19 would suggest a protective nature. Because we did not clearly detect any such peptides, our data do not support such a simple protective mechanism. Assessing the protective nature of these population-wide preexisting responses and cross-reactivities would require comparing samples of the same individuals before and after contracting COVID-19 and information about the course (severity) of the disease. The COVID-19 cohort of this study consisted of patients (testing positive in PCR and passing after infection serological tests) who had experienced mild symptoms and had not required hospitalization. Using our aCoV antigen library to compare antibody profiles against seasonal hCoVs between mild and severe COVID-19 cases could also inform about protective effects of cross-reactivity, as demonstrated for other aspects of the anti–SARS-CoV-2 immune response (*42*) and antibody binding of SARS-CoV-2 proteins (*13*, *14*). A theoretical finding that could point toward the protective nature of antibody responses against seasonal hCoVs would be, if severe patients exhibited fewer antibody responses against antigens of seasonal hCoVs than mild cases. Moreover, possible detrimental effects of cross-reactive antibody responses originating from seasonal hCoVs leading to antibody-dependent enhancement (*43*) could be assessed with our CoV antigen library.

Many studies examined the nature and functions of SARS-CoV-2–specific antibodies in patients with COVID-19 and identified mAbs with very potent neutralization activity (*32*–*35*). Nonetheless, it was shown that clearance of SARS-CoV, the pathogen that caused the first CoV pandemic, did not correlate with antibody neutralizing activity in a mouse model but rather antibody-dependent phagocytic activity of monocyte-derived alveolar macrophages played a key role (*44*). These findings suggest that cross-reactive antibodies that do not target the RBD might have beneficial functions through non-neutralizing activity. The recovered cross-reactive mAbs supported ADCP in vitro, and this function can potentially promote virus clearance in infected patients and antigen cross-presentation to T cells by antigen-presenting cells (*45*). In contrast, preexisting cross-reactive antibodies can form immune complexes and promote a deleterious over activation of the immune system (*46*); however, plasma transfer treatments suggest that this phenomenon plays a minor role if any in the context of SARS-CoV-2 (*7*).

Most of the described RBD-specific neutralizing antibodies show very few somatic hypermutations (SHMs), suggesting that they were recently generated in the recovered patients (*32*, *33*, *35*, *47*). Previous observations suggest a “back-boosting” effect in response to SARS-CoV-2 infection by preexisting antibodies or memory cells that were generated in response to common cold hCoVs (*7*, *12*, *13*). Because it is reasonable that most individuals encounter common cold hCoVs several times during their life (*48*–*51*), we hypothesize that preexisting cross-reactive antibodies would carry significant number of SHMs. The two broadly reactive antibodies that we cloned from rare memory cells carried more SHMs than those described previously for most neutralizing antibodies. This result points toward the possibility that these mAbs were present in the patient before exposure to SARS-CoV-2 and could possibly have been subjected to back-boosting. However, strengthening this hypothesis will require further experimental data including comparison of cross-reactive antibodies before and after exposure to SARS-CoV-2. Although the presence of cross-reactive antibodies was suggested to affect the severity of the disease (*13*), whether preexisting hCoV-specific memory cells with cross-reactive capacity provide an additional layer of protection against SARS-CoV-2 up on reexposure requires further investigation.

Leveraging the breadth of antibody responses against our CoV antigen library alongside machine learning algorithms allowed accurate separation between recovered patients with COVID-19 and unexposed individuals. Even when excluding all antigens of SARS-CoV2, antigens of aCoVs and the remaining hCoVs alone allowed separation of recovered patients with COVID-19 from unexposed individuals with similar accuracy as the complete antigen library. Hence, a phage-displayed antigen library consisting solely of CoVs deposited in databases as late as August 2018 (data file S1) allowed accurately diagnosis of the SARS-CoV-2 spillover with frequent infections emerging in the end of 2019. This ability to separate exposed and unexposed individuals without antigens of the causative CoV has implications for the serology of future spillovers of aCoVs to humans. With regularly updated antigen libraries representing emerging aCoV, this strategy could represent a readily available diagnostic test for detecting future transmission events alongside the collection of relevant infected and control cohorts (Fig. 7D). Rather than identifying and sequencing the causative new CoV and producing recombinant proteins as antigens for ELISA tests, large amounts of such coronavirome phage libraries can be produced well in advance and are readily available in case serology testing is required for a new spillover.

To effectively implement this strategy, three major steps are necessary, similarly outlined in plans for a global immunological observatory (*11*): (i) It will be necessary to monitor the animal coronavirome as a potential source of CoVs. This step will potentially involve sampling and metagenomics sequencing of the most common wildlife reservoir hosts and intermediate hosts (*52*). (ii) Using the genetic information of new aCoVs collected, it will be necessary to create and produce updated phage libraries representing the respective antigens at regular intervals. A plausible strategy would be to annually prepare such a library in autumn of the Northern Hemisphere to have an updated version available before the winter (with higher transmission rates of potential new spillovers due to more time spent indoors, etc.). (iii) Baseline samples of unexposed individuals (prepandemic samples) will be needed to compare against samples of individuals infected with a new aCoV spillover. Although, in principle, samples collected several years ago could be used, it would be preferable to use samples collected relatively close to a new outbreak, as exposure to seasonal hCoVs may change from year to year [as well as the impact of relatively quickly waning antibody titers is unclear (*48*)].

Furthermore, ongoing SARS-CoV-2 infections and vaccination campaigns may render the collection of annually updated reference cohorts advisable. At the time of writing, it is unclear how the COVID-19 pandemic will develop, whether global vaccination efforts will help to eradicate SARS-CoV-2, or whether it will transition to an endemic hCoV (*53*) such as current seasonal CoVs or influenza viruses. In either case, baseline antibody responses against CoVs are expected to have substantially changed before/after 2019/2020, with a potential impact on the detectability of future aCoV spillovers with our proposed strategy. Serological testing for future aCoV transmission events could be affected by elevated cross-reactivity arising from SARS-CoV-2 infections/vaccinations. However, any differences of a new CoV strain large enough not to be neutralized by existing immunity and promoting the spread of a new pandemic should also allow discrimination against existing CoVs with our proposed antigen library approach.

Ideally, these three key steps should be coordinated globally and implemented in a redundant fashion (i.e., sampling the animal coronavirome in many geographical locations, producing and storing phage libraries at multiple laboratories dispersed over the globe, and collecting baseline samples on all continents). Once a new aCoV spillover to human has been characterized in detail and reliable ELISAs or chemiluminescent immunoassays (CLIAs) are available, testing efforts will possibly completely shift away from PhIP-Seq/VirScan toward these higher-throughput methods. Although PhIP-Seq is highly cost efficient for testing large numbers of peptides in parallel (<$0.01 per peptide), the fixed assay cost of about $25 per sample (*54*) is greater than the cost of ELISAs or CLIAs detecting single antigens [or groups of peptides (*13*)]. Reducing the PhIP-Seq library size to fewer variants would not markedly reduce cost and hence would not favor the initial serological profiling of new zoonotic CoV spillovers. Different strategies used to select subsets of diagnostic peptides identified by a PhIP-Seq screen for more cost-effective diagnostic methods, such as CLIAs (*13*) and protein microarrays (*23*), can also be applied to aCoV antigens for the detection of new zoonotic spillovers.

In addition, our proposed strategy is limited to serology to detect antibodies generated upon exposure to potential novel aCoVs. DNA sequencing and reverse transcription quantitative PCR (RT-qPCR) testing will remain the dominant methodologies for detecting acute infections of novel CoV spillovers, an approach that our proposed strategy does not compete with. Antibody testing leveraging our library does, however, allow detection of past infections that have been cleared by the immune system (leaving no viral genetic material for nucleic acid–based testing). Applying this phage library for profiling antibody responses against novel CoV spillovers thereby allows the use of serology testing when ELISAs or CLIAs specific to the novel strain are not available yet. Hence, especially in the critical early phase of future pandemics, the immediate availability of an animal coronavirome serological assay allowing the screening of hundreds to thousands of samples for tens of thousands of antigens could represent a distinct advantage in containing the outbreak.

From a technical perspective, our study shares general limitations of PhIP-Seq (*20*), most notably length constraints of presented peptides (64 amino acids in this study) by underlying oligo synthesis and lack of eukaryotic PTMs such as glycosylation. Opposed to neutralization assays carried out with live viruses and cell cultures (*10*), our data do not inform about the neutralizing capacity of the observed binding events. Although linear epitopes should be adequately covered, discontinuous, conformational epitopes relying on the correct folding of domains could be missed. As a major limitation, we did not frequently detect binding to peptides of the RBD (with one adjacent peptide bound in 35.4% of COVID-19 and 0% of unexposed individuals’ sera; Fig. 3A), as similarly observed in other applications of PhIP-Seq/VirScan for CoV antibody profiling (*13*, *23*). Other technologies and diagnostic tests relying on the full-length RBD had reported common antibody responses in patients with COVID-19 (*15*, *16*, *55*, *56*). This discrepancy may be due to antibody responses against conformational epitopes in the RBD and/or a lack of S protein glycans (*57*) in the phage-displayed peptides. Hence, the conclusions of this study are limited to the antibody binding events detectable, and it is possible that binding to additional conformational epitopes or PTMs are missed.

Although current oligo lengths used in PhIP-Seq may underestimate conformational epitopes, it provides a unique layer of information unobtainable from working with full-length antigens or isolated domains. Given the high resolution of the peptide approach, we pinpoint the exact bound regions revealing crucial motifs responsible for cross-reactivities. We have also demonstrated that PhIP-Seq can represent a powerful method to identify the targets of recombinant human mAbs, including cross-reactive recognition of similar peptides.

Regarding the library content, we limited our design to about 13,000 peptides representing all hCoVs and 49 aCoVs, primarily from the NCBI RefSeq database. In principle, much larger PhIP-Seq libraries of hundreds of thousands of variants could be generated (*58*), also including less well-curated aCoV genomes beyond RefSeq. Our current library lacks, for example, pangolin CoVs that have been suggested to have contributed to the SARS-CoV-2 genome by recombination (*6*), which could be easily included in future designs.

In addition to informing about population-wide exposure (Fig. 7D), similarly designed aCoV libraries could also aid in identifying the animal source of future transmission events. For example, in our CoV antigen library, the most frequently bound aCoVs are two bat strains (fig. S5) closely related to SARS-CoV-2 (fig. S1), pointing to its origin. Furthermore, frequently antibody-bound motifs conserved between aCoVs could represent potential targets for broad spectrum anti-CoV vaccines to prevent future spillovers. However, it has been suggested that nonprotective antibody responses could also limit the generation of high-affinity B cells (epitope masking), which may hamper the generation of a universal aCoV vaccine (*7*). Hence, such vaccine designs will necessitate in depth feasibility assessments. Given the low cost of processing phage-displayed libraries in parallel (*54*), the method’s high accuracy (Fig. 7), and its excellent amenability for robot automation (*21*, *54*), regularly updated aCoV libraries could represent a useful tool for serological testing aiding containment in the early phases of future pandemics caused by spillover of aCoVs to humans.

## MATERIALS AND METHODS

### Samples

Serum samples (*n* = 269) of recovered patients with COVID-19 were obtained from MDA (Magen David Adom; the Israeli Red Cross equivalent). These samples had been collected between March and May 2020 from nonsevere cases, who had not been hospitalized. All patients were initially tested positive by RT-qPCR, and before sampling, patients had tested twice negative by RT-qPCR testing. Seropositivity of these samples had been confirmed by MDA with a commercial antibody test (Abbot, SARS-CoV-2 IgG, ref. 6R86-22/6R86-32). Control serum samples of unexposed individuals (*n* = 260) had been collected in 2013 to 2016 in Israel (39 in 2013, 138 in 2014, 78 in 2015, 5 in 2016) and reported previously (*25*, *26*). Research with the COVID-19 serum samples has been approved by the Weizmann Institute of Science’s institutional review board (#1030-4 and #1012-1) and by the Tel Aviv Sourasky Medical Center for the samples of unexposed individuals (#0658-12-TLV). The healthy cohort was selected to match (age/gender) the patients with COVID-19 as closely as possible, leaving minor differences (Fig. 1C). Although age/sex may influence COVID-19 serology of severe cases (*59*–*62*), we do not expect these parameters to affect key conclusions of our study in mildly affected patients [with both cohorts also showing similar antibody responses against viral controls (fig. S2B)]. We furthermore created perfectly age/gender-matched controls, by reducing sample numbers, which did not affect the separation of recovered patients with COVID-19 and unexposed individuals (fig. S10).

### hCoV and aCoV antigen library design

Reference genomes of the seven hCoVs were downloaded from NCBI directly using amino acid sequences of the translated ORFs with the accession numbers provided in data file S1. For each strain, the NSP part of the large polyprotein 1ab (polyprotein 1a was discarded if annotated) were separated. The SARS-CoV-2 polyprotein 1ab was cut according to the table published by Wu *et al.* (*1*). Additional strains’ polyproteins were processed by the following steps: NSP1 to NSP3 were cut by sequences reported in the literature (*63*). The remaining NSPs, which are naturally cut by 3C-like protease, were cut by the conserved protease cleavage site (small)-X-(L/I/V/F/M)-Q#(S/A/G), where X is any amino acid and # represents the cleavage position (*64*), and multiply sequence alignment as verification of the site. Specifically for SARS-CoV-2, four additional ORFs reported in the literature (*1*) (but not annotated in RefSeq NC_045512.2) were added. For SARS-CoV-2, in addition to the reference genome, variants deposited in the NCBI database mid-April of 2020 were included.

The protein sequences of aCoVs included within the library were also downloaded from NCBI RefSeq as protein sequences from the Coronaviridae family. Together, at the time of downloading (mid-April 2020), the data represented 48 aCoV strains (data file S1) composed of 677 proteins. Another bat CoV related to SARS-CoV-2 (*24*) was included in addition. The underlying protein sequences were processed by separating hCoV and aCoV proteins into two lists. For the human strains, the NSPs were processed as above, whereas for aCoVs, peptides were sequentially selected, omitting cleavage site predictions (if two polyproteins were annotated, only the longer one was kept).

The final list of proteins was cut to peptides of 64 amino acids with 20–amino acid overlaps [to cover all possible epitopes of the maximal length of linear epitope (*65*)] between adjacent peptides. The peptide amino acid sequences were reverse-translated to DNA using the *Escherichia coli* codon usage (of highly expressed proteins), aiming to preserve the original codon usage frequencies, excluding restriction sites for cloning (Eco RI and Hind III) within the coding sequence (CDS). The coding was reperformed, if needed, so that a barcode was formed in the CDS, by the 44 nucleotides (nt) at the 3′ end of each oligo. Every such barcode is a unique sequence at Hamming distance three from all prior sequences in the library, which allows for correcting of a single read error in sequencing the barcode. For similar peptide sequences, alternative codons were used after *E. coli* codon usage to achieve discrimination. Including the sequencing barcode as part of the CDS, rather than a separate barcode, allowed to use the entire oligo for encoding a peptide (and as opposed to completely omitting a barcode, it did not require sequencing the complete CDS). After finalizing the peptide sequence, the Eco RI and Hind III restriction sites, stop codon, and annealing sequences for library amplification were added and obtained from Agilent Technologies as 230-mer pool (library amplification primers, forward: GATGCGCCGTGGGAATTCT, reverse: GTCGGGTGGCAAGCTTTCA) and cloned into T7 phages following the manufacturer’s recommendations (Merck, T7Select10-3 Cloning Kit, product number 70550-3).

In this process, we had also included controls of viral proteins with high population-wide seroprevalence previously reported (*21*) and negative controls of 42 random peptides and a human protein (SAP4K, 27 peptides) not expected to elicit binding in healthy individuals. A full list of peptides included within the library as well as the corresponding amino acid and nucleotide sequences is provided in data file S2.

### Phage immunoprecipitation sequencing

The PhIP-Seq experiments were performed as outlined in a published protocol (*20*) with the following modifications: PCR plates for the transfer of beads and washing were blocked with 150 μl of bovine serum albumin (BSA) [30g/liter in Dulbecco’s phosphate-buffered saline (DPBS) buffer, incubation overnight at 4°C], and BSA was added to diluted phage/buffer mixtures for IPs to 2 g/liter. Three micrograms of serum IgG antibodies (measured by ELISA) were mixed with the phage library (4000-fold coverage of phages per library variant). Because technical replicates of the same sample were in excellent agreement, measurements were performed in single reactions.

The phage library and antibody mixtures were incubated in 96-deep-well plates at 4°C with overhead mixing on a rotator. Forty microliters of a 1:1 mixture of protein A and G magnetic beads (Thermo Fisher Scientific, catalog numbers 10008D and 10009D, washed according to the manufacturer’s recommendations) were added after overnight incubation and incubated on a rotator at 4°C. After 4 hours, the beads were transferred to PCR plates and washed twice as previously reported (*20*) using a Tecan Freedom Evo liquid handling robot with filter tips. The following PCR amplifications for pooled Illumina amplicon sequencing were performed with Q5 polymerase (New England Biolabs, catalog number M0493L) according to the manufacturer’s recommendations [primer pairs PCR1: tcgtcggcagcgtcagatgtgtataagagacagGTTACTCGAGTGCGGCCGCAAGC and gtctcgtgggctcggagatgtgtataagagacagATGCTCGGGGATCCGAATTC; PCR2: Illumina Nextera combinatorial dual index primers; PCR3 (of PCR2 pools): AATGATACGGCGACCACCGA and CAAGCAGAAGACGGCATACGA (*20*)]. PCR3 products were cut from agarose gel and purified twice (1x QIAquick Gel Extraction Kit, 1x QIAquick PCR purification kit; QIAGEN catalog numbers 28704/28104) and sequenced on an Illumina NextSeq machine (custom primers for R1: ttactcgagtgcggccgcaagctttca; R2: tgtgtataagagacagatgctcggggatccgaattct; R1/R2, 44/31 nt). Paired-end reads were processed as described below.

### Analysis of PhIP-Seq data

Enriched peptides per sample were calculated (after down-sampling to 800,000 identifiable reads per sample, i.e., reads with a barcode within one error of the set of possible barcodes of the library for which the paired end matched the identified oligo) by comparing reads from the IP reactions with antibodies against reads of input coverage (library sequencing of phages before IPs) after a generalized Poisson distribution approach, parameters for which were estimated for each sample separately, as previously reported (*27*). Derived *P* values were subject to Bonferroni correction (*P* = 0.05) for multiple hypothesis testing, and log fold change (number of reads of bound peptides versus baseline sequencing of phages not undergoing IPs) was computed for all peptides that passed the threshold *P* value; all other peptides were given a log fold change value of −4 (to be clearly marked).

All oligo creation code and analysis code were written in Python, using the libraries scikit-learn (*66*), scipy, statsmodels, pandas, numpy, and matplotlib. Custom code used for analyzing the PhIP-Seq data is publicly available at https://github.com/erans99/PhageIPSeq_CoVs. Alignments shown in Fig. 3 were created with CLC Main Workbench 6 (default settings).

### ELISA and peptide array assay

ELISA for SARS-CoV-2 trimeric spike protein was carried out using flat-bottom MaxiSorp 96-well plates (Invitrogen). The plates were coated with protein solution (5 μg/ml) in PBS at 100 μl per well and left overnight at 4°C. The plates were washed five times with washing buffer [1× PBS with 0.05% Tween 20 (Sigma-Aldrich)] and incubated with 100 μl of blocking buffer (1× PBS with 1% BSA) for 1 hour at room temperature. The blocking solution was subsequently replaced by serial dilutions of mAbs for 2.5 hours at room temperature. Plates were washed six times with washing buffer and then incubated with anti-human IgG secondary antibody conjugated to horseradish peroxidase (HRP) (Jackson ImmunoResearch) in blocking buffer at a 1:5000 dilution. Plates were developed by addition of the HRP substrate, TMB (3,3′,5,5′-tetramethylbenzidine) (Thermo Fisher Scientific), and absorbance was measured at 630 nm with an ELISA microplate reader (Synergy HT, BioTek). For testing binding to target peptides, the plates were first coated with streptavidin, followed by addition of biotinylated peptides.

CelluSpot hullB (Intavis) spike peptide arrays were used as per the manufacturer’s instructions. mAbs were used at 100 nM final concentration. GD01, an antibody that binds Junin virus glycoprotein, was used as an isotype control in the peptide array and PhIP-Seq experiments (without significant binding in either experimental system).

### Neutralization assay

Lentiviruses expressing S COVID-19 spikes were produced by transfecting human embryonic kidney (HEK) 293T cells with Luciferase-pLenti6, Δ19 S_covid-pCMV3, and ΔR89 Ψ vectors at 1:1:1 ratio, using Lipofectamine 2000 (Thermo Fisher Scientific). Medium containing lentiviruses was collected at 48 hours after transfection and centrifuged at 600*g* for 5 min for clarifying from cells, and aliquots were frozen at −80°C. For neutralization assays, HEK293T were transiently transfected with hACE2-pCDNA using Lipofectamine 2000. After 18-hour posttransfection, cells were reseeded on a poly-l-lysine–precoated white, chimney 96-well plates (Greiner Bio-One). Cells were left to adhere for 8 hours, followed by the addition of S-Covid19 lentivirions, which were preincubated with fourfold descending concentration series of mAbs. Luminescence from the activity of luciferase was measured 48 hours after infection using a Tecan Infinite M200 PRO plate reader after applying Bright-Glo reagent (Promega) on cells.

### Single-cell immunoglobulin analysis

For this purpose, we recruited two recovered patients who suffered from mild COVID-19–related symptoms 27 to 32 days after exposure and had initially been tested positive by a PCR. Spike reactive CD19^+^, CD27^+^, IgG1^+^, Igκ^+^ peripheral blood memory B cells were single cell–sorted into 96-well plates. These were processed and subjected to nested PCR amplification and Sanger sequencing of their heavy- and light-chain transcripts, as previously described (*30*). Upon collection of all immunoglobulin transcripts, data analysis was performed as detailed below.

### Determination of clonal expansion and SHMs

Immunoglobulin FASTA sequences were aligned against the IMGT human heavy-chain gene database (downloaded at December 2019) and light-chain gene database (downloaded at February 2017) using NCBI IgBLAST (version 1.17.0). Postprocessing of IgBLAST output and clonal clustering were performed using Change-O v0.4.6 (*67*), Alakazam v0.3.0, SHazaM v0.2.3, and custom scripts within the R statistical computing environment, as follows. V(D)J sequences were assigned to clonal groups by partitioning sequences based on identity of immunoglobulin heavy chain variable (IGHV) region gene annotations, immunoglobulin heavy joining (IGHJ) gene annotations, and junction region lengths. Within these groups, sequences differing from one another by a distance of more than 10 nt between the V genes were defined as separate clones. Full-length germline sequences were reconstructed for each clonal cluster with D segment and N/P regions masked (replaced with Ns), with any ambiguous gene assignments within clonal groups resolved by the majority rule. Circus plot were created using circlize R package v0.4.10.

### ADCP assay

ADCP was assessed by the measurement of the uptake of antibody-opsonized, antigen-coated fluorescent beads by THP-1 monocytic cell line. Briefly, 2 μg of biotinylated spike protein was used to saturate the binding sites of fluorescent NeutrAvidin beads (Invitrogen). Excess antigen was removed by washing the beads, which were then blocked with 1% BSA. Next, the beads were washed and incubated with either antibodies at a final concentration of 0.5 μM or serum from convalescent patients diluted to 1:100 for 2 hours at 37°C. The beads were washed, and unbound antibodies were removed. For measurement of phagocytic activity, THP-1 cells (American Type Culture Collection) were incubated with the coated beads for 1 hour at 37°C. The cells were then fixed, and the extent of phagocytosis was measured via flow cytometry (CytoFLEX). The data are reported as a phagocytic score, which takes into account the proportion of effector cells that phagocytosed and the degree of phagocytosis.

## Supplementary Material

20210729-1

## Supplementary Materials

### This PDF file includes:

Figs. S1 to S11 Table S1 Legends for data files S1 to S4 References (*71*–*73*)

## Notes

View/request a protocol for this paper from *Bio-protocol*.
