# Supplementary material for: Cross-reactive antibodies against human coronaviruses and the animal coronavirome suggest diagnostics for future zoonotic spillovers
Source: Sci Immunol. 2021 Jul 29;6(61):eabe9950. doi: 10.1126/sciimmunol.abe9950 (PMC9267281; doi:10.1126/sciimmunol.abe9950)
Supplement: 20210729-1 [file sciimmunol.abe9950.v1.pdf]

Cite as: S. Klompus *et al.*, *Sci. Immunol.* 10.1126/sciimmunol.abe9950 (2021).

CORONAVIRUS

Cross-reactive antibodies against human coronaviruses and the animal coronavirome suggest diagnostics for future zoonotic spillovers

Shelley Klompus[1,2#], Sigal Leviatan[1,2#], Thomas Vogl[1,2#*], Roei D. Mazor[3#], Iris N. Kalka[1,2], Liat Stoler-Barak[3], Nachum Nathan[3], Ayelet Peres[4], Lihee Moss[3], Anastasia Godneva[1,2], Sharon Kagan Ben Tikva[3], Eilat Shinar[5], Hadas Cohen Dvashi[6], Ronen Gabizon[6], Nir London[6], Ron Diskin[6], Gur Yaari[4], Adina Weinberger[1,2], Ziv Shulman[3*], Eran Segal[1,2*]

[1]Department of Computer Science and Applied Mathematics, Weizmann Institute of Science, Rehovot 76100, Israel. [2]Department of Molecular Cell Biology, Weizmann Institute of Science, Rehovot 76100, Israel. [3]Department of Immunology, Weizmann Institute of Science, Rehovot 76100, Israel. [4]Faculty of Engineering, Bar Ilan University, Ramat Gan 52900, Israel. [5]Magen David Adom Blood Services, Israel. [6]Department of Chemical and Structural Biology, Weizmann Institute of Science, Rehovot 7610001, Israel.

[#] Contributed equally

***Corresponding author. Email: thomas.vogl@weizmann.ac.il, ziv.shulman@weizmann.ac.il, eran.segal@weizmann.ac.il**

The spillover of animal coronaviruses (aCoVs) to humans has caused SARS, MERS, and COVID-19. While antibody responses displaying cross-reactivity between SARS-CoV-2 and seasonal/common cold human coronaviruses (hCoVs) have been reported, potential cross-reactivity with aCoVs and the diagnostic implications are incompletely understood. Here, we probed for antibody binding against all seven hCoVs and 49 aCoVs represented as 12,924 peptides within a phage-displayed antigen library. Antibody repertoires of 269 recovered COVID-19 patients showed distinct changes compared to 260 unexposed pre-pandemic controls, not limited to binding of SARS-CoV-2 antigens but including binding to antigens from hCoVs and aCoVs with shared motifs to SARS-CoV-2. We isolated broadly reactive monoclonal antibodies from recovered COVID-19 patients that bind a shared motif of SARS-CoV-2, hCoV-OC43, hCoV-HKU1, and several aCoVs, demonstrating that interspecies cross-reactivity can be mediated by a single immunoglobulin. Employing antibody binding data against the entire CoV antigen library allowed accurate discrimination of recovered COVID-19 patients from unexposed individuals by machine learning. Leaving out SARS-CoV-2 antigens and relying solely on antibody binding to other hCoVs and aCoVs achieved equally accurate detection of SARS-CoV-2 infection. The ability to detect SARS-CoV-2 infection without knowledge of its unique antigens solely from cross-reactive antibody responses against other hCoVs and aCoVs suggests a potential diagnostic strategy for the early stage of future pandemics. Creating regularly updated antigen libraries representing the animal coronavirome can provide the basis for a serological assay already poised to identify infected individuals following a future zoonotic transmission event.

INTRODUCTION

COVID-19 (coronavirus disease 2019), caused by SARS-CoV-2 (severe acute respiratory syndrome coronavirus 2), represents a pandemic with millions of cases worldwide. The related beta-coronaviruses SARS-CoV and MERS-CoV were the cause of the SARS outbreak in 2003 and Middle East respiratory syndrome (MERS) in 2012 (*1*). These three highly pathogenic coronaviruses (CoVs) are believed to represent spillovers of animal CoVs (aCoVs) to humans, with bats as the initial source (*2*, *3*). Additional intermediate animal hosts have possibly contributed to the transmission to humans, including palm civets as well as racoon dogs for SARS-CoV and camels for MERS-CoV. The intermediate host of SARS-CoV-2 is unclear, with a potential involvement of pangolins (*4*–*6*).

Given the large reservoir of aCoVs in the wild (i.e., the animal coronavirome) (*2*) and the possibility of recombination events leading to variants with an altered host spectrum (*6*), it has been speculated that more zoonotic transmissions of aCoVs to humans could happen in the future (*2*). To this end, broadly neutralizing vaccines targeting conserved regions of CoVs (*7*, *8*) and diagnostics for assessing their spread in humans could represent critical tools to counteract potential future pandemics. Serological assays based on antibody responses against pathogens are invaluable to inform on the

population-wide exposure to a pandemic (*9*, *10*). While testing based on the detection of viral nucleic acids informs on acute infections, antibody tests allow assessment of past exposure and can thereby reveal the contribution of asymptomatic cases possibly undetected by nucleic acid-based testing. The rapid availability of accurate serological tests (as well as access to pre-pandemic controls representing baseline antibody repertoires) could be key to increase the preparedness for future pandemics caused by zoonotic spillovers to humans (*11*).

However, the accuracy of serological tests can be perturbed by antibody cross-reactivity with similar antigens. Multiple CoV strains infect humans (hCoVs). In addition to SARS-CoV-2, SARS-CoV, and MERS-CoV, the seasonal endemic hCoVs (OC43, HKU1, NL63, 229E) are widely circulating in the population (*2*). Previous exposures to seasonal hCoVs could affect the accuracy of serological tests as well as potentially eliciting immunological memory that could affect the course of SARS-CoV-2 infections. While increasing amounts of data are accumulating on antibody cross-reactivity between hCoVs (*12*, *13*), cross-reactivity with the animal coronavirome and its diagnostic potential for detecting future spillovers of aCoVs to humans is incompletely understood. Also, the mechanism of cross-reactivity between hCoVs has not been characterized in detail. It remains unclear, whether multiple antibodies in patients' sera target different CoV-derived peptides or single monoclonal antibodies (mAbs) can mediate cross-reactivity between CoVs.

Assessing cross-reactivity against the animal coronavirome is challenging due to the large number of aCoV strains, necessitating immunological methods to probe for thousands of antigens in parallel. Antibody binding of antigens of SARS-CoV-2 is typically assessed by ELISAs against full length proteins/domains (*14*, *15*), by resolving crystal structures (*16*, *17*), or by peptide arrays (*18*, *19*). Pinpointing protein segments recognized by cross-reactive antibodies of all hCoVs and aCoVs requires high-resolution and high-throughput methods. Phage immunoprecipitation sequencing (PhIP-Seq) relies on the display of synthetic oligonucleotide libraries on T7 phages (*20*, *21*). Thereby the displayed antigens can be rationally selected allowing hundreds of thousands of antigens to be probed in parallel. After mixing of the phage library with serum antibodies, unbound phages are washed away after immunoprecipitation and enriched phages are detected by next generation sequencing (NGS) (Fig. 1a). PhIP-Seq has been adapted for assaying antibody binding against viruses (termed VirScan (*21*, *22*)) including SARS-CoV-2 and primarily other human hCoVs (*13*, *23*) as well as three bat CoVs (*13*). These studies have demonstrated suitability for diagnostic applications (*13*, *23*), as well as providing insights into cross-reactivity of hCoVs and COVID-19 severity (*13*). Limitations of PhIP-Seq (*20*) include length constraints of presented peptides (with short peptides inadequately representing conformational epitopes) and lack of eukaryotic post-translational modifications (PTMs), that also affect the detectability of CoV antigens (discussed in detail in the Discussion).

Here, we have generated a PhIP-Seq/VirScan library covering all seven hCoVs and 49 aCoVs originating from diverse hosts including bats, rodents, domestic animals and birds, represented as 12,924 peptides. We demonstrate that human serum antibody cross-reactivity extends beyond hCoVs to aCoVs and can be mediated by single mAbs. This pronounced cross-reactivity allows accurate detection of SARS-CoV-2 exposure without using any SARS-CoV-2 peptides and hence suggests diagnostic applications for the early stage of future pandemics caused by zoonotic spillovers of viruses to humans.

RESULTS

A library of 12,924 hCoV and aCoV peptides

We designed a PhIP-Seq library (experimental outline is shown in Fig. 1a) covering all open reading frames of human and animal CoVs as 64 amino acid (aa) sections with 20 aa overlaps between adjacent peptides (Fig. 1b, Data file S1). The sequences of 48 aCoVs were obtained from the NCBI reference genome (RefSeq, April 2020) database and the sequence of another bat CoV related to SARS-CoV-2 (*24*) was included in addition. These strains broadly covered all groups of α, β, γ, and δ CoVs with their phylogeny illustrated in Fig. S1. The CoV antigen library consisted in total of 12,924 peptides, with hCoVs representing ~20% of peptides and aCoVs ~80% of CoV antigens (Fig. 1b, Data file S2). The 49 aCoVs contained 11 strains infecting birds, 19 strains infecting bats, and 19 more CoVs infecting other mammals such as rabbits, rodents, bovines *etc.* (see Data file S1 for a full list of aCoVs and hosts). For SARS-CoV-2, in addition to the reference genome, variants deposited in the NCBI database as of mid-April of 2020 were included. The antigen library also includes positive controls which confirmed detection of antibody responses against viruses previously reported to elicit population-wide immunity (*21*) and negative controls, that did not show substantial binding (Fig. S2).

We tested IgG antibody binding against this CoV library with 260 pre-pandemic serum samples of individuals unexposed to SARS-CoV-2, that had been collected in 2013 to 2016 (*25*, *26*) (Fig. 1c). These antibody repertoires were compared to 269 samples of recovered COVID-19 patients obtained in April and May 2020. The serum samples were mixed individually with the phage library displaying the CoV antigens (Fig. 1a). Phages bound by antibodies were immunoprecipitated and unbound phages washed away. The bound phages were PCR amplified and sequenced. Thereby we obtained for each library variant in each sample a read count after immunoprecipitation. These read counts were compared to the 'input' read counts of the phage material before mixing with serum

samples. We employed a Generalized Poisson distribution approach previously reported (*27*) to calculate p-values for significance of the enrichment of each library variant in each sample. These p-values were filtered in each sample by strict Bonferroni correction (<0.05) to counteract the problem of multiple hypothesis testing (see materials and methods for details). In total we have assayed for approximately five million antibody-peptide interactions (12,924 hCoV peptides in each of the 529 individuals). On average of 114 CoV peptides were significantly bound per unexposed individual and 189 CoV peptides per recovered COVID-19 patient. Most analyses were based on antibody responses against 579 CoV peptides from different CoV strains and proteins, shared by more than five percent of either group (Data file S3). Out of these, 190 peptides showed significantly different abundances between the groups (Data file S3). Bound antigens included peptides originating from hCoVs (Fig. 2, Fig. 3) as well as aCoVs (Fig. 4), which are discussed sequentially in the following sections.

Antibody repertoires against hCoVs and cross-reactivities

As expected, sera of recovered COVID-19 patients bound significantly more peptides of SARS-CoV-2 than sera of unexposed individuals (Fig. 2a). Also many peptides of SARS-CoV, MERS-CoV, as well as the seasonal hCoVs OC43 and HKU1 were significantly more frequently bound in recovered patients than unexposed individuals, in line with cross-reactivity previously reported (*13*).

Unexposed individuals showed abundant antibody responses against all seasonal hCoVs (Fig. 1e-h): Peptides of hCoV-NL63 were significantly bound in up to 88% of unexposed individuals, peptides of hCoV-HKU1 in up to 87%, peptides of hCoV-229E in up to 83%, and peptides of hCoV-OC43 in up to 46% (Fig. 1e-h, Data file S2). The same peptides were bound at similar frequencies in recovered COVID-19 patients and originated mostly from S- or N-proteins (Fig. 3, Data file S3) with these epitope resolved results being in agreement with previous studies on the seroprevalence of seasonal hCoVs using ELISAs (*28*).

Recovered COVID-19 patients' sera showed an overrepresentation of several peptides of SARS-CoV-2 that showed no binding or binding at very low percentages in unexposed individuals (Fig. 3b). Twelve peptides passed FDR (Benjamini Hochberg false discovery rate (*29*), a method to correct for multiple hypothesis testing) correction for being significantly different between the two groups of individuals (for the SARS-CoV-2 reference genome, additional peptides of SARS-CoV-2 variants passed significance thresholds and are listed in Data file S3). While nearly all COVID-19 patients showed binding against at least one peptide in S- or N-proteins and some peptides being bound in up to 81.4% of recovered patients (Fig. 1b), no convergence of antibody responses against the same peptide were detected in all individuals (which is limited to the interactions detectable with this PhIP-Seq library, see the discussion section). This finding differs from near universal recognition of some viral epitopes previously observed by PhIP-Seq/VirScan for other human viruses (*12*) and replicated with controls in this study (Fig. S2), suggesting that the antibody response against SARS-CoV-2 can exhibit substantial inter-individual variability.

COVID-19 serum samples also showed common binding against SARS-CoV (Fig. 2c), to which it is unlikely that these individuals had been exposed, indicating detection of cross-reactivity of antibodies targeting SARS-CoV-2 (*14*, *15*). Most notably three SARS-CoV spike peptides had significantly enriched binding in up to 88.1% of COVID-19 recovered individuals compared to up to 6.9% of unexposed individuals (Fig. 3c, Data file S3). A non-structural protein (NSP2) of SARS-CoV was even bound in 30% of unexposed individuals and 36% of recovered, possibly owing to higher conservation of such NSPs underlying less selective pressure than S- and N-proteins mostly responsible for infectivity and targeted by neutralizing immune responses. Also three peptides from the MERS-CoV (Fig. 3d) S-protein were differentially enriched in the COVID-19 recovered cohort, passing FDR correction for significance of this difference (Fig. 3a, Data file S3).

Strikingly, cross-reactive responses from SARS-CoV-2 also extended to the spike proteins all four seasonal hCoVs (Fig. 3a) related to specific motifs (Fig. 3c,d). Three peptides from each hCoV-OC43 and hCoV-HKU1 were significantly differentially bound between unexposed and recovered individuals. One of these peptides arose from similar regions of the S protein (FP site, Fig. 3a) and also hCoV-229E and hCoV-NL63 peptides around this position were significantly differentially bound. Such cross-reactivity has been primarily reported for hCoV-OC43 (*13*). Our data suggests, that cross-reactivity arising from SARS-CoV-2 infection targets a similar motif in all human hCoVs (RSXIEDLLFXK, Fig. 3c, see Fig. S3a for an alignment of the complete bound peptides and Fig. S4 illustrating that the motif is exposed on the surface of SARS-CoV-2). Toward the C terminus of the spike protein, another region was bound at significantly different frequencies between recovered and unexposed individuals in SARS-CoV-2, SARS-CoV, MERS-CoV, hCoV-OC43, and hCoV-HKU1 (Fig. 3a). This longer region appeared to contain two distinct motifs (Fig. 3d, Fig. S3b, and Fig. S4 illustrating surface exposure on the SARS-CoV-2 spike protein). In the nucleocapsid protein, less clear motifs were apparent in regions bound by antibodies at similar levels between hCoVs (Fig. 3b and Fig. S3c,d) potentially owing to separate binding of smaller motifs by different antibodies (which may explain why some of the peptides were bound at different levels and others not Fig. 3b).

Antibody cross-reactivity extends toward animal coronaviruses

In addition to antibody binding to hCoV peptides, we

detected also dozens of aCoV peptides significantly bound per individual (Fig. 4a). These peptides originated from aCoVs with diverse hosts including all three major groups: bats, other mammals (such as rodents), and birds. Recovered COVID-19 patients' sera bound significantly more aCoV peptides than unexposed individuals when scoring these groups (Fig. 4a). Scoring differences on a strain level (Fig. S5) showed varying antibody responses, as observed for hCoVs (Fig. 2a): Peptides of some bat CoVs closely related to SARS-CoV-2 (Fig. S1) were highly significant for being more frequently bound in recovered COVID-19 patients, than in unexposed individuals (p-value $<10^{-4}$). In contrast, peptides of bat CoVs such as a NL-63 related strain were not bound to a different extent between the two groups (p-value >0.05, Fig. S5). This finding is in agreement with the seasonal hCoV-NL63 also not exhibiting a significant difference (Fig. 1a). Similarly, for aCoVs of birds or other mammalian hosts, we observed some strains being bound by antibodies at similar levels in unexposed and recovered COVID-19 patients, and other strains bound differentially. For example aCoVs from rabbit, mouse rat, pig and cow as well as the bird night heron showed highly significant differences between the two groups, whereas aCoVs from felines, ferrets and camel did not show significant differences (Fig. S5). We also analyzed differences in antibody binding based on genetic differences of the aCoVs (Fig. S6). These results suggest that antibody responses against beta aCoVs show the greatest statistical difference between unexposed and recovered individuals (as expected from their closer relationship to the beta hCoV SARS-CoV-2). Differential responses were not limited to beta aCoVs with some antigens of alpha, gamma, and delta aCoVs also being significantly more frequently bound by antibodies in recovered COVID-19 patients than unexposed individuals (Fig. S6).

Relating these antibody responses to hCoVs, aCoV peptides amounted for approximately 80% of the initial library content while hCoVs represented about 20% (Fig. 4b, Fig. 1b). In recovered COVID-19 patients, antibody responses against SARS-CoV-2 were overrepresented, especially regarding peptides bound in larger fractions of the cohort (Fig. 4b). In unexposed individuals, aCoV peptides took up a larger fraction of bound peptides with seasonal hCoVs still being overrepresented. These results suggest that peptides of hCoVs are dominantly bound by human antibodies and aCoVs contribute to the detected antibody repertoire by cross-reactivity.

To pinpoint the potential cross-reactivity underlying the binding signal within aCoVs, we mapped the bound aCoV peptides to the SARS-CoV-2 proteome (summarized in Fig. 4c,d, see Data file S3 for alignment data for all peptides). Two clusters in the S protein near the FP site and the C terminus were apparent (Fig. 4c, Fig. S7a,b), which are similar to the cross-reactive regions observed in hCoVs (Fig. 3a,c,d; (*13*), accessible on the surface of the S protein Fig. S4). Near the FP site, the RSXIEDLLFXK motif observed from hCoVs (Fig. 3c) appeared in near identical form in aCoVs (Fig. 4e). The N-terminal cluster yielded for aCoVs even a clearer motif (FKEELDXXFKN, Fig. 4f) than the same region in hCoVs (Fig. 3d), possibly owing to the larger number of peptides aligned. In the N-protein, bound aCoV peptides clustered mostly in the RNA binding domain (Fig. 4d), exhibiting also a shared motif (Fig. 4 g and see Fig. **S7**c,d for full alignments). While similar peptides in the RNA binding domain of the N-protein of hCoVs are bound (Fig. 3c), the exact motif identified in aCoVs (Fig. 4 g) was not apparent. Furthermore, a C-terminal motif found in aCoVs (Fig. 4 g) did not have a direct equivalent in hCoVs (Fig. 3b). The two main spike motifs detected from aCoV peptides (Fig. 4e,f) also match sequences previously reported in a different cohort (*13*) suggesting that human antibodies raised upon SARS-CoV-2 infection can also cross-react with aCoVs. Additionally, cross-reactivity of human antibodies against aCoVs appears to arise also from seasonal hCoVs, as some S- and N- peptides are bound at similar levels in unexposed controls and recovered COVID-19 patients. These include rat/mouse CoV peptides around amino acid 600 in the S-protein (Fig. 4c) or bat peptides around amino acid 100 in the N-protein (Fig. 4d).

Patient-derived immunoglobulins with cross-reactivity potential

While we observed pronounced antibody cross-reactivity between SARS-CoV-2 and hCoVs as well as aCoVs (Fig. 2 to Fig. 4), the underlying mechanisms are unclear. Cross-reactivity is most likely to arise from polyclonal antibodies, yet, the occurrence of shared motifs (Fig. 3c,d, Fig. 4e-h) suggests potential cross-reactive recognition by a single antibody. To examine this possibility, we sequenced B cell immunoglobulin genes derived from recovered patients and generated monoclonal antibodies (*30*) for testing in PhIP-Seq. Since cross reactive binding to the receptor binding domain (RBD) is less likely to occur, we sequenced patient-derived B cells that show reactivity with the intact spike trimer that carries more conserved domains with cross-reactive potential. Spike-specific single memory B cells (CD19+, CC27+, IgG1+, Igκ+) were sorted from peripheral blood mononuclear cells of convalescent patients and subjected to PCR amplification of their immunoglobulin genes followed by gene sequencing (Fig. 5a). Analysis of the emerging immunoglobulin sequences revealed significant enrichment for VH3 and VK3 and these types of V genes were the most abundant paired chains (Fig. 5b,c). Based on the variable region (VDJ) sequences we clustered the clones to determine the clonal expansion of spike-specific memory B cells. This analysis revealed that most of the spike-binding memory B cells were not significantly expanded, and the majority of the recovered immunoglobulin sequences appeared only once (Fig. 5b). The average number of somatic mutations per cell was 16 and 10 for the heavy and

light chain, respectively (Fig. 5d), which is slightly higher than the average mutation load of memory cells in healthy subjects (*31*). As expected, the number of mutations in the heavy chains was in correlation to the mutation load in the light chains (Fig. 5e) and the CDR3 lengths of the light and heavy chains (Fig. 5f) were similar to typical immunoglobulins in naive B cells (*31*). Comparing our findings to previous published data shows that the frequency of mutations was slightly higher in our cohort, however, the observed clonal expansion was lower (Table S 1). It is important to note, that most studies focused on the RBD target whereas we used the full spike trimer as a bait which carry multiple domains. We conclude that although robust clonal expansion was not detected in recovered patients, some of the mutated spike-specific memory cells are the product of antibody affinity maturation and may have broad binding reactivity.

Pan-specific mAbs can mediate cross-reactivity against hCoVs and aCoVs

In order to test the patient-derived immunoglobulins in PhIP-Seq, we cloned the recovered sequences into expression vectors and produced monoclonal antibodies (*30*). Previous studies found that most of the SARS-CoV-2 neutralizing antibodies are very similar to their germline configuration (*32*–*35*). In contrast, broadly reactive antibodies generated during chronic infection, such as in HIV-infected patients, are highly mutated (*36*). Furthermore, if SARS-CoV-2 activates pre-existing cross-reactive memory cells that were raised against other corona viruses, the emerging antibodies are expected to carry more mutations than those elicited in a primary response. Therefore, to maximize the probability for detection of broadly reactive mAbs, two highly mutated antibodies were chosen for expression as IgG1 (*30*) followed by spike-binding and PhIP-Seq analyses. In parallel, we also examined additional two antibodies that carried very few somatic mutations, WIS-A7 and WIS-A9 for cross-reactivity comparison. ELISA revealed four antibodies that showed detectable spike-binding activity (Fig. 6a). Among these antibodies, C1 and C3 mAbs carried a significant load of mutations and shared 88.7-90.1% VH sequence identity (Fig. 6b). Using our PhIP-seq assay, we found that both C1 and C3 bound significantly to several peptides of hCoVs (SARS-CoV-2, SARS-CoV, hCoV-OC43, hCoV-HKU1) as well as several aCoVs (including bovine, rodent, thrush (bird), rabbit, and bat as hosts) spike proteins found within our phage display library (Fig. 6c, left and middle panel, see Fig. S8 for a full list of peptides). In contrast a control monoclonal antibody did not show any significantly enriched peptides. C1 and C3 showed nearly identical binding to the same peptides, with slight differences for two peptides (Fig. 6c, right panel), suggesting that the additional mutations of C3 do not strongly impact cross-reactivity against our CoV antigen library. Consistently, a part of the SARS-CoV-2 peptide target was detected in a spike peptide array binding assay (Fig. 6d,e). The sequence of the binding site was very similar among the different strains of corona viruses and was composed of a consensus motif that is located in the S2 domain of the SARS-CoV-2 spike protein (Fig. 6f). This motif lies in a region different from frequently bound peptides identified in hCoVs (Fig. 3a) and aCoVs (Fig. 4c). In agreement with these results, the mAbs also bound the target peptide in ELISA (Fig. 6 g). Since, structurally, part of this peptide sequence is not exposed on the spike trimer surface, it is most likely that the mAbs bind only the N terminus of the peptide (Fig. 6e,h). A7 mAb bound a peptide that was derived from the SARS-CoV-2 spike protein and a very similar peptide that is part of a bat SARS like aCoV spike complex (Fig. S9a). This region of the SARS-CoV-2 spike protein (starting at amino acid 748), was a binding target in 35.3% of our recovered COVID-19 cohort, but not in our healthy control cohort (Fig. 3a). Although A9 was found to bind the spike protein in ELISA, our PhIP-Seq assay did not identify a linear binding target (Fig. S9b). This contradiction can be a result of A9 binding to a non-linear epitope or posttranslational modifications that are inadequately represented within our phage library (see also the discussion section below). Overall, for the highly mutated antibodies showed significant cross-reactivity, providing a proof of concept evidence for the existence that a single antibody can bind multiple corona viruses. Yet, elucidating the frequency of single cross-reactive antibodies in the general population will require further studies that involves pre- and post-exposure to SARS-CoV-2 analyses.

To examine if cross-reactive monoclonal antibodies support protective functional activity, we examined their capacity to neutralize pseudovirus that carried the SARS-CoV-2 spike protein. Both the C1 and C3 displayed poor neutralization activity compared with a control antibody that was previously described (*35*) (Fig. 6h). Typically, IgG1 antibodies support non-neutralizing activity as well, however, since C1 and C3 mAbs bind an internal epitope that is exposed only in the open conformation of the spike trimer, they might not be able to support these functions. Antibody-dependent cellular phagocytosis (ADCP) is a process wherein immune cells uptake a target through antibody-mediated interaction with Fc receptors (*37*, *38*). To examine ADCP activity, we produced immune complexes by incubating spike-coated beads with monoclonal antibodies and tested the ability of THP-1 monocytes to uptake these targets. Both C1 and C3 monoclonal antibodies promoted the uptake of spike-coated beads by THP-1 cells whereas the control antibody did not contribute to this process (Fig. 6j). To validate if serum antibodies can support ADCP, we examined if patient-derived serum can promote phagocytic activity. We found that all of the patients have antibodies in their serum that can support ADCP (Fig. 6k). This antibody-mediated activity can be supported by few or multiple neutralizing and non-neutralizing antibodies.

Collectively, we conclude that cross-reactive monoclonal antibodies derived from COVID-19 patients show functional activity.

To examine if the monoclonal antibodies were subjected to affinity maturation we examined if amino acid replacements in the complementary determining regions and in the framework regions of the V regions using the Baseline tool (*39*, *40*). Similar to other antigen-experienced B cells, the mutation patterns in these sequences indicate that the B cells producing these IgGs experienced statistically significant negative selection in the FWR (framework region) in both the heavy and the light chain. As for the CDRs, the selection estimations are not significant (not negative and not positive in neither chain) (Fig. S11). This observation is typical when mAb selection is estimated based on a few sequences. These data indicate that according to the selection patterns it seems that these cells were subjected to typical antigen-experienced maturation process that involved negative (purifying) selection in the framework regions and nonnegative in the complementary determining regions.

Animal coronavirome-based high throughput diagnostics

Testing for multiple CoV antigens at high resolution in parallel could yield higher specificity than conventional tests based on single proteins of SARS-CoV-2 by improving discrimination from seasonal hCoVs. Most current SARS-CoV-2 serological tests rely on detection of entire S-/RBD or N-proteins, reporting an aggregate of binding against all epitopes within (*10*, *15*). PhIP-Seq implementations of CoV antigen libraries primarily based on hCoVs have also demonstrated highly accurate diagnostic performance (*13*, *23*). We set out to benchmark our extensive CoV antigen library containing both hCoVs and a large set of aCoVs for diagnostic applications. Interpreting the epitope-resolved antibody binding data reported by our assay extends beyond conventional serological tests, as binding against various epitopes of all hCoV needs to be weighed. We used gradient boosting trees (XGBoost) to build a predictor that accurately separates COVID-19 patients from healthy controls based on antibody signatures (Fig. 7, Fig. S10) when trained on antibody binding data of the complete library (AUC=0.980, Fig. 7a). Depending on the intended application and cutoffs employed, this assay displays with this set of samples 100% specificity at 87% sensitivity (reporting virtually no false-positives for this set of samples) or 92% specificity at 95% sensitivity (Fig. 7a PR curve). Interestingly, even the subset of antibody binding data solely against peptides of aCoVs allowed to accurately separate between exposed and unexposed individuals (AUC=0.96, Fig. 7b).

When looking at the principal components of an analysis (PCA, Fig. 7a,b) performed on the log fold change (number of reads of bound peptides vs. baseline sequencing of phages not undergoing immunoprecipitation) of significantly enriched peptides, unexposed individuals clustered together while recovered COVID-19 patients' samples showed a greater spread. This illustrates the interindividual variability in hCoV antibody responses elicited by SARS-CoV-2 and the PCA separation based on the entire CoV antigen library (Fig. 7a) or the subset of aCoV antigens (Fig. 7b) yielded similar results. This interindividual variability in COVID-19 recovered patients may represent differences in immune responses or could also be affected by the time passed between infection and blood sampling (although this period is <3 months for our COVID-19 cohort where anti-SARS-CoV-2 antibody responses may show relatively high stability (*41*)).

As outlined above, aCoVs alone are enough to accurately separate between exposed and unexposed individuals. When further adding other hCoVs except SARS-CoV-2 to the aCoVs (Fig. 7c), the accuracy slightly increased to nearly the same level as using the complete library including SARS-CoV-2 antigens (AUC=0.97, Fig. 7a). Also binding data against hCoVs peptides excluding SARS-CoV-2 allowed for accurate detection of past infection (Fig. 7c). SARS-CoV-2 antigens alone yielded lower predictive power, but including also peptides originating of SARS-CoV-2 variants improved the AUC (Fig. 7c). This difference is possibly owing to the low redundancy in the included SARS-CoV-2 peptides of the reference strain, which improves when adding slightly different variants (that potentially do not strongly affect antibody binding). In this respect, including identical SARS-CoV-2 reference peptides in the input library, could also be a strategy to improve the performance of diagnostic PhIP-Seq assays. However, given the already large redundancy arising from non-SARS-CoV-2 hCoVs and aCoVs in our existing library, it does not appear necessary to also add duplicates of peptides from these groups. Taken together, these results indicate, that is possible to accurately detect SARS-CoV-2 exposure, without including actual SARS-CoV-2 antigens, suggesting a potential diagnostic strategy for the early stage of future pandemics.

DISCUSSION

Overall, our data indicates that antibody cross-reactivity observed for hCoVs (*12*, *13*) extends to aCoVs and PhIP-Seq/VirScan is a feasible strategy to capture cross-reactive antibody responses at an epitope resolved resolution. Peptides bound in a larger fraction of individuals typically featured similar motifs and isolation of human mAbs confirmed binding of single antibodies to multiple hCoV and aCoV peptides bearing a similar motif. Cross-reactivities against aCoVs observed in our study can be assigned to at least two causes: 1.) Cross-reactivity in individuals unexposed to SARS-CoV-2, possibly owing to exposure with seasonal hCoVs. These responses included peptides of rodent CoVs bound in up to 67% of unexposed individuals mapping to the SARS-CoV-2 S-protein (Fig. 4c around amino acid 600) as well as bat and rat

peptides mapping to the SARS-CoV-2 N-protein (bound in up to 89% of unexposed individuals; Fig. 4d). 2.) Cross-reactivity toward hCoVs and aCoVs caused by SARS-CoV-2 infection. Such cross-reactivity has previously been reported against seasonal hCoVs and three bat CoVs closely related to SASRS-CoV-2 (*13*). Our data indicates that cross-reactivity induced by SARS-CoV-2 infection extends beyond bat CoVs, including also peptides of aCoVs from diverse animals as rodents, rabbit, mink, porcine, bovine as well as birds (*e.g.* night heron) as natural host (among others Data file S3).

A potential third aspect of cross-reactivity would be represented by pre-existing immunity against seasonal hCoVs protecting against SARS-CoV-2 infection (*12*) (or even providing protection against potential future spillovers of aCoVs to humans). hCoV or aCoV peptides bound at higher abundance in unexposed individuals than recovered COVID-19 patients, would suggest a protective nature. As we did not clearly detect any such peptides our data does not support such a simple protective mechanism. Assessing the protective nature of these population-wide preexisting responses and cross-reactivities would require comparing samples of the same individuals before and after contracting COVID-19 and information on the course (severity) of the disease. The COVID-19 cohort of this study consisted of patients (testing positive in PCR and passing post infection serological tests) who had experienced mild symptoms, and had not required hospitalization. Employing our aCoV antigen library to compare antibody profiles against seasonal hCoVs between mild and severe COVID-19 cases could also inform on protective effects of cross-reactivity, as demonstrated for other aspects of the anti-SARS-CoV-2 immune response (*42*) and antibody binding of SARS-CoV-2 proteins (*13*, *14*). A theoretical finding that could point toward the protective nature of antibody responses against seasonal hCoVs would be, if severe patients exhibited fewer antibody responses against antigens of seasonal hCoVs than mild cases. Moreover, possible detrimental effects of cross-reactive antibody responses originating from seasonal hCoVs leading to antibody-dependent enhancement (*43*) could be assessed with our CoV antigen library.

Many studies examined the nature and functions of SARS-CoV-2-specific antibodies in COVID-19 patients and identified mAbs with very potent neutralization activity (*32–35*). Nonetheless, it was shown that clearance of SARS-CoV, the pathogen that caused the first coronavirus pandemic, did not correlate with antibody neutralizing activity in a mouse model but rather antibody-dependent phagocytic activity of monocyte-derived alveolar macrophages played a key role (*44*). These findings suggest that cross-reactive antibodies that do not target the RBD might have beneficial functions through non-neutralizing activity. Indeed, the recovered cross-reactive monoclonal antibodies supported ADCP in vitro and this function can potentially promote virus clearance in infected patients as well as antigen cross presentation to T cells by antigen presenting cells (*45*). In contrast, pre-existing cross-reactive antibodies can form immune complexes and promote a deleterious over activation of the immune system (*46*); however plasma transfer treatments suggest that this phenomena plays a minor role if any in the context of SARS-CoV-2 (*7*).

Most of the described RBD-specific neutralizing antibodies show very few somatic hyper mutations (SHM), suggesting that they were recently generated in the recovered patients (*32*, *33*, *35*, *47*). Previous observations suggest a "back-boosting" effect in response to SARS-CoV-2 infection by pre-existing antibodies or memory cells that were generated in response to common cold hCoVs (*7*, *12*, *13*). Since it is reasonable that most individuals encounter common cold hCoVs several times during their life (*48–51*), we hypothesize that pre-existing cross-reactive antibodies would carry significant number of SHM. The two broadly reactive antibodies that we cloned from rare memory cells carried more SHM than those described previously for most neutralizing antibodies. This result points toward the possibility that these mAbs were present in the patient before exposure to SARS-CoV-2 and could possibly have been subjected to "back-boosting". ". However, strengthening this hypothesis will require further experimental data including comparison of cross-reactive antibodies before and after exposure to SARS-CoV-2. Although the presence of cross-reactive antibodies was suggested to affect the severity of the disease (*13*), whether pre-existing hCoV-specific memory cells with cross reactive capacity provide an additional layer of protection against SARS-CoV-2 up on re-exposure requires further investigation.

Leveraging the breadth of antibody responses against our CoV antigen library alongside machine learning algorithms allowed to accurately separate between recovered COVID-19 patients and unexposed individuals. Strikingly, even when excluding all antigens of SARS-CoV2, antigens of aCoVs and the remaining hCoVs alone allowed to separate recovered COVID-19 patients from unexposed individuals with similar accuracy as the complete antigen library. Hence, a phage displayed antigen library consisting solely of CoVs deposited in databases as late as August 2018 (Data file S1), allowed to accurately diagnose the SARS-CoV-2 spillover with frequent infections emerging in the end of 2019. This ability to separate exposed and unexposed individuals without antigens of the causative CoV has implications for the serology of future spillovers of aCoVs to humans. With regularly updated antigen libraries representing emerging animal coronaviruses, this strategy could represent a readily available diagnostic test for detecting future transmission events alongside the collection of relevant infected and control cohorts (Fig. 7d). Rather than identifying and sequencing the causative new CoV as well as producing recombinant proteins as antigens

for ELISA tests, large amounts of such coronavirome phage libraries can be produced well in advance and are readily available in case serology testing is required for a new spillover.

To effectively implement this strategy, three major steps are necessary, similarly outlined in plans for a global immunological observatory (*11*): 1.) It will be necessary to monitor the animal coronavirome as potential source of CoVs. This step will potentially involve sampling and metagenomics sequencing of the most common wildlife reservoir hosts as well as intermediate hosts (*52*). 2.) Using the genetic information of new aCoVs collected, it will be necessary to create and produce updated phage libraries representing the respective antigens at regular intervals. A plausible strategy would be to annually prepare such a library in autumn of the Northern Hemisphere to have an updated version available before the winter (with higher transmission rates of potential new spillovers due to more time spent indoors, etc.). 3.) Baseline samples of unexposed individuals (pre-pandemic samples) will be needed to compare against samples of individuals infected with a new aCoV spillover. While in principle samples collected several years ago could be used, it would be preferable to use samples collected relatively close to a new outbreak, as exposure to seasonal hCoVs may change from year to year (also the impact of relatively quickly waning antibody titers is unclear (*48*)).

Furthermore, ongoing SARS-CoV-2 infections as well as vaccination campaigns may render the collection of annually updated reference cohorts advisable. At the time of writing, it is unclear how the COVID-19 pandemic will develop and if global vaccination efforts will help to eradicate SARS-CoV-2, or if it will transition to an endemic hCoV (*53*) such as current seasonal CoVs or Influenza viruses. In either case, baseline antibody responses against CoVs are expected to have substantially changed pre/post 2019/2020, with a potential impact on the detectability of future aCoV spillovers with our proposed strategy. Serological testing for future aCoV transmission events could be affected by elevated cross-reactivity arising from SARS-CoV-2 infections/vaccinations. However, any differences of a new CoV strain large enough not to be neutralized by existing immunity and promoting the spread of a new pandemic, should also allow to discriminate against existing CoVs with our proposed antigen library approach.

Ideally, these three key steps should be coordinated globally and implemented in a redundant fashion (*i.e.* sampling the animal coronavirome in many geographical locations, producing and storing phage libraries at multiple laboratories dispersed over the globe, as well as collecting baseline samples on all continents). Once a new aCoV spillover to human has been characterized in detail and reliable ELISAs or CLIAs are available, testing efforts will possibly completely shift away from PhIP-Seq/VirScan toward these higher throughput methods. While PhIP-Seq is highly cost-efficient for testing large numbers of peptides in parallel (< $0.01 per peptide), the fixed assay cost of approximately $25 per sample (*54*) is greater than the cost of ELISAs or CLIAs detecting single antigens (or groups of peptides (*13*)). Reducing the PhIP-Seq library size to fewer variants would not drastically reduce cost and hence would not favor the initial serological profiling of new zoonotic CoV spillovers. Different strategies used to select subsets of diagnostic peptides identified by a PhIP-Seq screen for more cost-effective diagnostic methods, such as CLIAs (*13*) and protein microarrays (*23*), can also be applied to aCoV antigens for the detection of new zoonotic spillovers.

Additionally, our proposed strategy is limited to serology to detect antibodies generated upon exposure to potential novel aCoVs. DNA sequencing and RT-qPCR testing will remain the dominant methodologies for detecting acute infections of novel CoV spillovers, an approach that our proposed strategy does not compete with. Antibody testing leveraging our library does however allow detection of past infections that have been cleared by the immune system (leaving no viral genetic material for nucleic-acids based testing). Applying this phage library for profiling antibody responses against novel CoV spillovers thereby allows use of serology testing when ELISAs or CLIAs specific to the novel strain are not available yet. Hence, especially in the critical early phase of future pandemics, the immediate availability of an animal coronavirome serological assay allowing the screening of hundreds to thousands of samples for tens of thousands of antigens could represent a distinct advantage in containing the outbreak.

From a technical perspective, our study shares general limitations of PhIP-Seq (*20*), most notably length constraints of presented peptides (64 aa in this study) by underlying oligo synthesis and lack of eukaryotic post translational modifications (PTMs) such as glycosylation. Opposed to neutralization assays carried out with live viruses and cell cultures (*10*), our data does not inform on the neutralizing capacity of the observed binding events. While linear epitopes should be adequately covered, discontinuous, conformational epitopes relying on the correct folding of domains could be missed. As a major limitation, we did not frequently detect binding to peptides of the RBD (with one adjacent peptide bound in 35.4% of COVID-19 and 0% of unexposed individuals' sera, Fig. 3a), as similarly observed in other applications of PhIP-Seq/VirScan for CoV antibody profiling (*13*, *23*). Other technologies and diagnostic tests relying on the full length RBD had reported common antibody responses in COVID-19 patients (*15*, *16*, *55*, *56*). This discrepancy may be due to antibody responses against conformational epitopes in the RBD and/or a lack of S protein glycans (*57*) in the phage displayed peptides. Hence the conclusions of this study are limited to the

antibody binding events detectable and it is possible that binding to additional conformational epitopes or PTMs are missed.

While current oligo lengths employed in PhIP-Seq may underestimate conformational epitopes, it provides a unique layer of information unobtainable from working with full length antigens or isolated domains. Given the high resolution of the peptide approach, we pinpoint the exact bound regions revealing crucial motifs responsible for cross-reactivities. We have also demonstrated that PhIP-Seq can represent a powerful method to identify the targets of recombinant human mAbs, including cross-reactive recognition of similar peptides.

Regarding the library content, we limited our design to ca. 13,000 peptides representing all hCoVs and 49 aCoVs, primarily from the NCBI RefSeq database. In principle, much larger PhIP-Seq libraries of hundreds of thousands of variants could be generated (*58*), including also less well curated aCoV genomes beyond RefSeq. Our current library lacks for example pangolin CoVs, that have been suggested to have contributed to the SARS-CoV-2 genome by recombination (*6*), which could be easily included in future designs.

In addition to informing on population-wide exposure (Fig. 7d), similarly designed aCoV libraries could also aid in identifying the animal source of future transmission events. For example, in our CoV antigen library the most frequently bound aCoVs are two bat strains (Fig. **S5**) closely related to SARS-CoV-2 (Fig. S1), pointing to its origin. Furthermore, frequently antibody-bound motifs conserved between aCoVs could represent potential targets for broad spectrum anti-CoV vaccines to prevent future spillovers. However, it has been suggested that non-protective antibody responses could also limit the generation of high affinity B cells (epitope masking), which may hamper the generation of a universal aCoV vaccine (*7*). Hence such vaccine designs will necessitate in depth feasibility assessments. Given the low cost of processing phage-displayed libraries in parallel (*54*), the method's high accuracy (Fig. 7), and its excellent amenability for robot automation (*21*, *54*), regularly updated aCoV libraries could represent a useful tool for serological testing aiding containment in the early phases of future pandemics caused by spillover of aCoVs to humans.

MATERIALS AND METHODS

Samples

Serum samples (n=269) of recovered COVID-19 patients were obtained from MDA (Magen David Adom, the Israeli Red Cross equivalent). These samples had been collected between March and May 2020 from non-severe cases, who had not been hospitalized. All patients were initially tested positive by RT-qPCR, and before sampling, patients had tested twice negative by RT-qPCR testing. Seropositivity of these samples had been confirmed by MDA with a commercial antibody test (Abbot, SARS-CoV-2 IgG, ref. 6R86-22/6R86-32). Control serum samples of unexposed individuals (n=260) had been collected in 2013 to 2016 in Israel (39 in 2013, 138 in 2014, 78 in 2015, 5 in 2016) and reported previously (*25*, *26*). Research with the COVID-19 serum samples has been approved by the Weizmann Institute of Science's institutional review board (#1030-4 and #1012-1), and by the Tel Aviv Sourasky Medical Center for the samples of unexposed individuals (#0658-12-TLV). The healthy cohort was selected to match (age/gender) the COVID-19 patients as closely as possible, leaving minor differences (Fig. 1c). While age/sex may influence COVID-19 serology of severe cases (*59*–*62*), we do not expect these parameters to affect key conclusions of our study in mildly affected patients (with both cohorts also showing similar antibody responses against viral controls [Fig. S2b]). We furthermore created perfectly age/gender matched controls, by reducing sample numbers, which did not affect the separation of recovered COVID-19 patients and unexposed individuals (Fig. **S10**).

hCoV and aCoV antigen library design

Reference genomes of the seven hCoVs were downloaded from NCBI directly using amino acid sequences of the translated ORFs with the accession numbers provided in Data file S1. For each strain the nonstructural proteins (NSPs) part of the large polyprotein 1ab (polyprotein 1a was discarded if annotated) were separated. The SARS-CoV-2 polyprotein 1ab was cut according to the table published by Wu *et al.* (*1*). Additional strains' polyproteins were processed by the following steps: NSPs 1-3 were cut by sequences reported in the literature (*63*). The remaining NSPs, which are naturally cut by 3Clike protease (3CLpro), were cut by the conserved protease cleavage site (small)-X-(L/I/V/F/M)-Q#(S/A/G), where X is any amino acid and # represents the cleavage position (*64*), and multiply sequence alignment as verification of the site. Specifically for SARS-CoV-2, four additional ORFs reported in the literature (*1*) (but not annotated in RefSeq NC_045512.2) were added. For SARS-CoV-2, in addition to the reference genome, variants deposited in the NCBI database mid-April of 2020 were included.

The protein sequences of aCoVs included within the library were also downloaded from NCBI RefSeq as protein sequences from the Coronaviridae family. All together at the time of downloading (mid-April 2020) the data represented 48 aCoV strains (Data file S1) composed of 677 proteins. Another bat CoV related to SARS-CoV-2 (*24*) was included in addition. The underlying protein sequences were processed by separating hCoV and aCoV proteins into two lists. For the human strains, the NSPs were processed as above, whereas for aCoVs peptides were sequentially selected, omitting cleavage site predictions (if two polyproteins were annotated only the longer one was kept).

The final list of proteins was cut to peptides of 64 amino

acids (aa) with 20 aa overlaps (to cover all possible epitopes of the maximal length of linear epitope (*65*)) between adjacent peptides. The peptide aa sequences were reverse translated to DNA using the *Escherichia coli* codon usage (of highly expressed proteins), aiming to preserve the original codon usage frequencies, excluding restriction sites for cloning (*Eco*RI and *Hind*III) within the coding sequence (CDS). The coding was re-performed, if needed, so that a barcode was formed in the CDS, by the 44 nt at the 3′ end of each oligo. Every such barcode is a unique sequence at Hamming distance three from all prior sequences in the library, which allows for correcting of a single read error in sequencing the barcode. For similar peptide sequences, alternative codons were used following *E. coli* codon usage, to achieve discrimination. Including the sequencing barcode as part of the CDS, rather than a separate barcode, allowed to use the entire oligo for encoding a peptide (and as opposed to completely omitting a barcode, it did not require sequencing the complete CDS). After finalizing the peptide sequence, the *Eco*RI and *Hind*III restriction sites, stop codon, and annealing sequences for library amplification were added and obtained from Agilent Technologies as 230mer pool (library amplification primers, fwd: GATGCGCCGTGGGAATTCT, rev: GTCGGGTGGCAAGCTTTCA) and cloned into T7 phages following the manufacturer's recommendations (Merck, T7Select®10-3 Cloning Kit, product number 70550-3).

In this process we had also included controls of viral proteins with high population-wide seroprevalence previously reported (*21*) and negative controls of 42 random peptides and a human protein (SAP4K, 27 peptides) not expected to elicit binding in healthy individuals. A full list of peptides included within the library as well as the corresponding amino acid and nucleotide sequences is provided in Data file S2.

Phage immunoprecipitation sequencing

The PhIP-Seq experiments were performed as outlined in a published protocol (*20*) with the following modifications: PCR plates for the transfer of beads and washing were blocked with 150 μL BSA (30 g/L in DPBS buffer, incubation overnight at 4°C) and BSA was added to diluted phage/buffer mixtures for immunoprecipitations (IPs) to 2 g/L. Three microgram of serum IgG antibodies (measured by ELISA) were mixed with the phage library (4,000-fold coverage of phages per library variant). As technical replicates of the same sample were in excellent agreement, measurements were performed in single reactions.

The phage library and antibody mixtures were incubated in 96 deep well plates at 4°C with overhead mixing on a rotator. Forty microliters of a 1:1 mixture of protein A and G magnetic beads (Thermo Fisher Scientific, catalog numbers 10008D and 10009D, washed according to the manufacturer's recommendations) were added after overnight incubation and incubated on a rotator at 4°C. After four hours, the beads were transferred to PCR plates and washed twice as previously reported (*20*) using a Tecan Freedom Evo liquid handling robot with filter tips. The following PCR amplifications for pooled Illumina amplicon sequencing were performed with Q5 polymerase (New England Biolabs, catalog number M0493L) according to the manufacturer's recommendations (primer pairs PCR1: tcgtcggcagcgtcagatgtgtataagagacagGTTACTCGAGTGCGGCCGCAAGC and gtctcgtgggctcggagatgtgtataagagacagATGCTCGGGGATCCGAATTC, PCR2: Illumina Nextera combinatorial dual index primers, PCR3 [of PCR2 pools]: AATGATACGGCGACCACCGA and CAAGCAGAAGACGGCATACGA (*20*)). PCR3 products were cut from agarose gel and purified twice (1x QIAquick Gel Extraction Kit, 1x QIAquick PCR purification kit; Qiagen catalog numbers 28704/28104) and sequenced on an Illumina NextSeq machine (custom primers for R1: ttactcgagtgcggccgcaagctttca, and for R2: tgtgtataagagacagatgctcggggatccgaattct, R1/R2 44/31 nts). Paired end reads were processed as described below.

Analysis of PhIP-Seq data

Enriched peptides per sample were calculated (after down-sampling to 800,000 identifiable reads per sample, i.e., reads with a barcode within one error of the set of possible barcodes of the library for which the paired end matched the identified oligo) by comparing reads from the immunoprecipitation reactions with antibodies against reads of input coverage (library sequencing of phages before IPs) following a Generalized Poisson distribution approach, parameters for which were estimated for each sample separately, as previously reported (*27*). Derived p-values were subject to Bonferroni correction (p-value 0.05) for multiple hypothesis testing, and log-fold-change (number of reads of bound peptides vs. baseline sequencing of phages not undergoing IPs) was computed for all peptides which passed the threshold p-value, all other peptides were given a log-fold-change value of -4 (to be clearly marked).

All oligo creation code, and analysis code was written in Python, using the libraries scikit-learn (*66*), scipy, statsmodels, pandas, numpy and matplotlib. Custom code used for analyzing the PhIP-Seq data are publicly available: https://github.com/erans99/PhageIPSeq_CoVs. Alignments shown in Fig. 3 were created with CLC Main Workbench 6 (default settings).

ELISA and peptide array assay

ELISA for SARS-CoV-2 trimeric spike protein was carried out using flat-bottom MaxiSorp 96-well plates (Invitrogen). The plates were coated with 5 μg/ml protein solution in PBS at 100 μl per well and left overnight at 4°C. The plates were washed 5 times with washing buffer (1× PBS with 0.05% Tween-20 (Sigma-Aldrich)) and incubated with 100 μl blocking buffer (1× PBS with 1% BSA) for 1h at room temperature (RT). The blocking solution was subsequently replaced by

serial dilutions of monoclonal antibodies for 2.5h at RT. Plates were washed 6 times with washing buffer and then incubated with anti-human IgG secondary antibody conjugated to horseradish peroxidase (HRP) (Jackson ImmunoResearch) in blocking buffer at a 1:5,000 dilution. Plates were developed by addition of the HRP substrate, TMB (Thermo Fisher) and absorbance was measured at 630 nm with an ELISA microplate reader (Synergy HT, BioTek). For testing binding to target peptides, the plates were first coated with streptavidin followed by addition of biotinylated peptides.

CelluSpot hullB (Intavis) spike peptide arrays were used per the manufacturer's instructions. Monoclonal antibodies were used at 100 nM final concentration. GD01, an antibody that binds Junin virus glycoprotein, was used as an isotype control in the peptide array and PhIP-Seq experiments (without significant binding in either experimental system).

Neutralization assay

Lentiviruses expressing S-Covid19 spikes were produced by transfecting HEK293T cells with Luciferase-pLenti6, ∆19 S_covid-pCMV3 and ∆R89 Ψ vectors at 1:1:1 ratio, using Lipofectamine 2000 (Thermo Fisher). Media containing lentiviruses was collected at 48 hours post-transfection, centrifuged at 600x g for 5 min for clarifying from cells, and aliquots were frozen at -80°C. For neutralization assays, HEK293T were transiently transfected with hACE2-pCDNA using Lipofectamine 2000. Following 18 hours post-transfection, cells were re-seeded on a poly-L-lysine pre-coated white, chimney 96-well plates (Greiner Bio-One). Cells were left to adhere for 8 hours, followed by the addition of S-Covid19 lentivirions, which were pre-incubated with 4-fold descending concentration series of monoclonal antibodies. Luminescence from the activity of luciferase was measured 48 hours post-infection using a Tecan Infinite M200 PRO plate reader after applying Bright-Glo reagent (Promega) on cells.

Single-cell immunoglobulin analysis

For this purpose, we recruited two recovered patients that suffered from mild COVID-19-related symptoms 27-32 days after exposure and had initially been tested positive by a PCR. Spike reactive CD19+, CD27+, IgG1+, Igκ+ peripheral blood memory B cells were single cell sorted into 96-well plates. These were processed and subjected to nested PCR amplification and Sanger sequencing of their heavy and light chain transcripts, as previously described (*30*). Upon collection of all immunoglobulin transcripts, data analysis was performed as detailed below.

Determination of clonal expansion and somatic hypermutations

Ig FASTA sequences were aligned against the IMGT human heavy chain gene database (downloaded at Dec. 2019) and light chain gene database (downloaded at Feb. 2017) using NCBI IgBLAST (version 1.17.0). Post processing of IgBLAST output, and clonal clustering were performed using Change-O v0.4.6 (*67*), Alakazam v0.3.0, SHazaM v0.2.3, and custom scripts within the R statistical computing environment, as follows. V(D)J sequences were assigned to clonal groups by partitioning sequences based on identity of IGHV gene annotations, IGHJ gene annotations, and junction region lengths. Within these groups, sequences differing from one another by a distance of more than 10 nucleotides between the V genes were defined as separate clones. Full-length germline sequences were reconstructed for each clonal cluster with D segment and N/P regions masked (replaced with Ns), with any ambiguous gene assignments within clonal groups resolved by the majority rule. Circus plot were created using circlize R package v0.4.10.

ADCP assay

ADCP (antibody-dependent cellular phagocytosis) was assessed by the measurement of the uptake of antibody-opsonized, antigen-coated fluorescent beads by THP-1 monocytic cell line. Briefly, 2 μg of biotinylated spike protein was used to saturate the binding sites of fluorescent NeutrAvidin beads (Invitrogen). Excess antigen was removed by washing the beads, which were then blocked with 1% BSA. Next, the beads were washed and incubated with either antibodies at a final concentration of 0.5 μM or serum from convalescent patients diluted to 1:100 for 2h at 37°C. The beads were washed, and unbound antibodies were removed. For measurement of phagocytic activity, THP-1 cells (ATCC) were incubated with the coated beads for 1h at 37°C. The cells were then fixed, and the extent of phagocytosis was measured via flow cytometry (CytoFLEX). The data are reported as a phagocytic score, which takes into account the proportion of effector cells that phagocytosed and the degree of phagocytosis.

SUPPLEMENTARY MATERIALS

immunology.sciencemag.org/cgi/content/full/6/61/eabe9950/DC1

Fig. S1: A phylogenetic tree of all hCoVs and aCoVs included in the phage antigen library.
Fig. S2: Control antigens included in the library.
Fig. S3: Full alignments of hCoV peptides.
Fig. S4: Peptides bound by antibodies in recovered COVID-19 patients and featuring conserved motifs between hCoVs and aCoVs lie on the surface of the SARS-CoV-2 spike protein.
Fig. S5: Antibody responses against antigens of the 49 animal coronaviruses included within the library.
Fig. S6: Antibody responses against aCoVs by genetic distance and grouping of α, β, γ, and δ aCoVs.
Fig. S7: Full alignments of aCoV peptides shown in Fig. 4.
Fig. S8: List of peptides significantly bound by the mAbs WIS-C1 and WIS-C3 and alignments.
Fig. S9: PhIP-Seq based identification of targeted peptides within the CoV-antigen library of two additional human mAbs.
Fig. S10: Matching recovered COVID-19 patients and unexposed controls by age or year of birth does not affect the accurate separation of the two groups by a machine learning classifier from antibody repertoire data.
Fig. S11: Affinity driven selection quantification.
Table S1: Comparison of the current findings to previous studies.
Data file S1: List of the human and animal coronaviruses included in the antigen phage library.
Data file S2: Full list of peptides included within the library as well as the corresponding

amino acid sequences (including controls).
Data file S3: Most frequent hCoV/aCoV antigens bound in unexposed individuals and recovered COVID-19 patients.
Data file S4: Raw data file.

REFERENCES AND NOTES

1. A. Wu, Y. Peng, B. Huang, X. Ding, X. Wang, P. Niu, J. Meng, Z. Zhu, Z. Zhang, J. Wang, J. Sheng, L. Quan, Z. Xia, W. Tan, G. Cheng, T. Jiang, Genome Composition and Divergence of the Novel Coronavirus (2019-nCoV) Originating in China. *Cell Host Microbe* **27**, 325–328 (2020). doi:10.1016/j.chom.2020.02.001 Medline
2. A. C. Walls, Y.-J. Park, M. A. Tortorici, A. Wall, A. T. McGuire, D. Veesler, Structure, Function, and Antigenicity of the SARS-CoV-2 Spike Glycoprotein. *Cell* **181**, 281–292.e6 (2020). doi:10.1016/j.cell.2020.02.058 Medline
3. B. P. Zhou, Z. Shi, SARS-CoV-2 spillover events (2021).
4. P. Liu, J.-Z. Jiang, X.-F. Wan, Y. Hua, L. Li, J. Zhou, X. Wang, F. Hou, J. Chen, J. Zou, J. Chen, Are pangolins the intermediate host of the 2019 novel coronavirus (SARS-CoV-2)? *PLOS Pathog.* **16**, e1008421 (2020). doi:10.1371/journal.ppat.1008421 Medline
5. J. Zhao, W. Cui, B. P. Tian, The Potential Intermediate Hosts for SARS-CoV-2. *Front. Microbiol.* **11**, 580137 (2020). doi:10.3389/fmicb.2020.580137 Medline
6. T. T. Y. Lam, N. Jia, Y.-W. Zhang, M. H.-H. Shum, J.-F. Jiang, H.-C. Zhu, Y.-G. Tong, Y.-X. Shi, X.-B. Ni, Y.-S. Liao, W.-J. Li, B.-G. Jiang, W. Wei, T.-T. Yuan, K. Zheng, X.-M. Cui, J. Li, G.-Q. Pei, X. Qiang, W. Y.-M. Cheung, L.-F. Li, F.-F. Sun, S. Qin, J.-C. Huang, G. M. Leung, E. C. Holmes, Y.-L. Hu, Y. Guan, W.-C. Cao, Identifying SARS-CoV-2-related coronaviruses in Malayan pangolins. *Nature* **583**, 282–285 (2020). doi:10.1038/s41586-020-2169-0 Medline
7. J. J. Guthmiller, P. C. Wilson, Remembering seasonal coronaviruses. *Science* **370**, 1272–1273 (2020). doi:10.1126/science.abf4860 Medline
8. A. A. Cohen, P. N. P. Gnanapragasam, Y. E. Lee, P. R. Hoffman, S. Ou, L. M. Kakutani, J. R. Keeffe, H. J. Wu, M. Howarth, A. P. West, C. O. Barnes, M. C. Nussenzweig, P. J. Bjorkman, Mosaic nanoparticles elicit cross-reactive immune responses to zoonotic coronaviruses in mice. *Science* **371**, 735–741 (2021). Medline
9. C. J. E. Metcalf, J. Farrar, F. T. Cutts, N. E. Basta, A. L. Graham, J. Lessler, N. M. Ferguson, D. S. Burke, B. T. Grenfell, Use of serological surveys to generate key insights into the changing global landscape of infectious disease. *Lancet* **388**, 728–730 (2016). doi:10.1016/S0140-6736(16)30164-7 Medline
10. F. Krammer, V. Simon, Serology assays to manage COVID-19. *Science* **368**, 1060–1061 (2020). doi:10.1126/science.abc1227 Medline
11. M. J. Mina, C. J. E. Metcalf, A. B. McDermott, D. C. Douek, J. Farrar, B. T. Grenfell, A Global Immunological Observatory to meet a time of pandemics. *eLife* **9**, 1–5 (2020). doi:10.7554/eLife.58989 Medline
12. K. W. Ng, N. Faulkner, G. H. Cornish, A. Rosa, R. Harvey, S. Hussain, R. Ulferts, C. Earl, A. G. Wrobel, D. J. Benton, C. Roustan, W. Bolland, R. Thompson, A. Agua-Doce, P. Hobson, J. Heaney, H. Rickman, S. Paraskevopoulou, C. F. Houlihan, K. Thomson, E. Sanchez, G. Y. Shin, M. J. Spyer, D. Joshi, N. O'Reilly, P. A. Walker, S. Kjaer, A. Riddell, C. Moore, B. R. Jebson, M. Wilkinson, L. R. Marshall, E. C. Rosser, A. Radziszewska, H. Peckham, C. Ciurtin, L. R. Wedderburn, R. Beale, C. Swanton, S. Gandhi, B. Stockinger, J. McCauley, S. J. Gamblin, L. E. McCoy, P. Cherepanov, E. Nastouli, G. Kassiotis, Preexisting and de novo humoral immunity to SARS-CoV-2 in humans. *Science* **370**, 1339–1343 (2020). doi:10.1126/science.abe1107 Medline
13. E. Shrock, E. Fujimura, T. Kula, R. T. Timms, I.-H. Lee, Y. Leng, M. L. Robinson, B. M. Sie, M. Z. Li, Y. Chen, J. Logue, A. Zuiani, D. McCulloch, F. J. N. Lelis, S. Henson, D. R. Monaco, M. Travers, S. Habibi, W. A. Clarke, P. Caturegli, O. Laeyendecker, A. Piechocka-Trocha, J. Z. Li, A. Khatri, H. Y. Chu, A.-C. Villani, K. Kays, M. B. Goldberg, N. Hacohen, M. R. Filbin, X. G. Yu, B. D. Walker, D. R. Wesemann, H. B. Larman, J. A. Lederer, S. J. Elledge; MGH COVID-19 Collection & Processing Team, Viral epitope profiling of COVID-19 patients reveals cross-reactivity and correlates of severity. *Science* **370**, 1–23 (2020). doi:10.1126/science.abd4250 Medline
14. C. Atyeo, S. Fischinger, T. Zohar, M. D. Slein, J. Burke, C. Loos, D. J. McCulloch, K. L. Newman, C. Wolf, J. Yu, K. Shuey, J. Feldman, B. M. Hauser, T. Caradonna, A. G. Schmidt, T. J. Suscovich, C. Linde, Y. Cai, D. Barouch, E. T. Ryan, R. C. Charles, D. Lauffenburger, H. Chu, G. Alter, Distinct Early Serological Signatures Track with SARS-CoV-2 Survival. *Immunity* **53**, 524–532.e4 (2020). Medline
15. R. Weissleder, H. Lee, J. Ko, M. J. Pittet, COVID-19 diagnostics in context. *Sci. Transl. Med.* **12**, 1–6 (2020). doi:10.1126/scitranslmed.abc1931 Medline
16. B. Ju, Q. Zhang, J. Ge, R. Wang, J. Sun, X. Ge, J. Yu, S. Shan, B. Zhou, S. Song, X. Tang, J. Yu, J. Lan, J. Yuan, H. Wang, J. Zhao, S. Zhang, Y. Wang, X. Shi, L. Liu, J. Zhao, X. Wang, Z. Zhang, L. Zhang, Human neutralizing antibodies elicited by SARS-CoV-2 infection. *Nature* **584**, 115–119 (2020). doi:10.1038/s41586-020-2380-z Medline
17. C. O. Barnes, A. P. West Jr., K. E. Huey-Tubman, M. A. G. Hoffmann, N. G. Sharaf, P. R. Hoffman, N. Koranda, H. B. Gristick, C. Gaebler, F. Muecksch, J. C. C. Lorenzi, S. Finkin, T. Hägglöf, A. Hurley, K. G. Millard, Y. Weisblum, F. Schmidt, T. Hatziioannou, P. D. Bieniasz, M. Caskey, D. F. Robbiani, M. C. Nussenzweig, P. J. Bjorkman, Structures of human antibodies bound to SARS-CoV-2 spike reveal common epitopes and recurrent features of antibodies. *Cell* **182**, 828–842.e16 (2020). doi:10.1016/j.cell.2020.06.025 Medline
18. H. W. Jiang, Y. Li, H. N. Zhang, W. Wang, X. Yang, H. Qi, H. Li, D. Men, J. Zhou, S. C. Tao, SARS-CoV-2 proteome microarray for global profiling of COVID-19 specific IgG and IgM responses. *Nat. Commun.* **11**, 3581 (2020). doi:10.1038/s41467-020-17488-8 Medline
19. Li *et al*., Linear epitope landscape of SARS-CoV-2 Spike protein constructed from 1,051 COVID-19 patients, (2020), medRxiv doi:10.1101/2020.07.13.20152587
20. D. Mohan, D. L. Wansley, B. M. Sie, M. S. Noon, A. N. Baer, U. Laserson, H. B. Larman, PhIP-Seq characterization of serum antibodies using oligonucleotide-encoded peptidomes. *Nat. Protoc.* **13**, 1958–1978 (2018). doi:10.1038/s41596-018-0025-6 Medline
21. G. J. Xu, T. Kula, Q. Xu, M. Z. Li, S. D. Vernon, T. Ndung'u, K. Ruxrungtham, J. Sanchez, C. Brander, R. T. Chung, K. C. O'Connor, B. Walker, H. B. Larman, S. J. Elledge, Viral immunology. Comprehensive serological profiling of human populations using a synthetic human virome. *Science* **348**, aaa0698 (2015). doi:10.1126/science.aaa0698 Medline
22. M. J. Mina, T. Kula, Y. Leng, M. Li, R. D. de Vries, M. Knip, H. Siljander, M. Rewers, D. F. Choy, M. S. Wilson, H. B. Larman, A. N. Nelson, D. E. Griffin, R. L. de Swart, S. J. Elledge, Measles virus infection diminishes preexisting antibodies that offer protection from other pathogens. *Science* **366**, 599–606 (2019). doi:10.1126/science.aay6485 Medline
23. C. R. Zamecnik, J. V. Rajan, K. A. Yamauchi, S. A. Mann, R. P. Loudermilk, G. M. Sowa, K. C. Zorn, B. D. Alvarenga, C. Gaebler, M. Caskey, M. Stone, P. J. Norris, W. Gu, C. Y. Chiu, D. Ng, J. R. Byrnes, X. X. Zhou, J. A. Wells, D. F. Robbiani, M. C. Nussenzweig, J. L. DeRisi, M. R. Wilson, ReScan, a Multiplex Diagnostic Pipeline, Pans Human Sera for SARS-CoV-2 Antigens. *Cell Reports Med.* **1**, 100123 (2020). doi:10.1016/j.xcrm.2020.100123 Medline
24. B. Coutard, C. Valle, X. de Lamballerie, B. Canard, N. G. Seidah, E. Decroly, The spike glycoprotein of the new coronavirus 2019-nCoV contains a furin-like cleavage site absent in CoV of the same clade. *Antiviral Res.* **176**, 104742 (2020). doi:10.1016/j.antiviral.2020.104742 Medline
25. D. Zeevi, T. Korem, N. Zmora, D. Israeli, D. Rothschild, A. Weinberger, O. Ben-Yacov, D. Lador, T. Avnit-Sagi, M. Lotan-Pompan, J. Suez, J. A. Mahdi, E. Matot, G. Malka, N. Kosower, M. Rein, G. Zilberman-Schapira, L. Dohnalová, M. Pevsner-Fischer, R. Bikovsky, Z. Halpern, E. Elinav, E. Segal, Personalized Nutrition by Prediction of Glycemic Responses. *Cell* **163**, 1079–1094 (2015). doi:10.1016/j.cell.2015.11.001 Medline
26. T. Korem, D. Zeevi, N. Zmora, O. Weissbrod, N. Bar, M. Lotan-Pompan, T. Avnit-Sagi, N. Kosower, G. Malka, M. Rein, J. Suez, B. Z. Goldberg, A. Weinberger, A. A. Levy, E. Elinav, E. Segal, Bread Affects Clinical Parameters and Induces Gut Microbiome-Associated Personal Glycemic Responses. *Cell Metab.* **25**, 1243–1253.e5 (2017). doi:10.1016/j.cmet.2017.05.002 Medline
27. H. B. Larman, Z. Zhao, U. Laserson, M. Z. Li, A. Ciccia, M. A. M. Gakidis, G. M. Church, S. Kesari, E. M. Leproust, N. L. Solimini, S. J. Elledge, Autoantigen discovery with a synthetic human peptidome. *Nat. Biotechnol.* **29**, 535–541 (2011). doi:10.1038/nbt.1856 Medline
28. G. J. Gorse, G. B. Patel, J. N. Vitale, T. Z. O'Connor, Prevalence of antibodies to four human coronaviruses is lower in nasal secretions than in serum. *Clin. Vaccine Immunol.* **17**, 1875–1880 (2010). doi:10.1128/CVI.00278-10 Medline
29. Y. Benjamini, Y. Hochberg, Controlling the False Discovery Rate: A Practical and Powerful Approach to Multiple Testing. *J. R. Stat. Soc. B* **57**, 289–300 (1995). doi:10.1111/j.2517-6161.1995.tb02031.x
30. T. Tiller, E. Meffre, S. Yurasov, M. Tsuiji, M. C. Nussenzweig, H. Wardemann, Efficient generation of monoclonal antibodies from single human B cells by single

cell RT-PCR and expression vector cloning. *J. Immunol. Methods* **329**, 112–124 (2008). doi:10.1016/j.jim.2007.09.017 Medline
31. M. Ghraichy, J. D. Galson, A. Kovaltsuk, V. von Niederhäusern, J. Pachlopnik Schmid, M. Recher, A. J. Jauch, E. Miho, D. F. Kelly, C. M. Deane, J. Trück, Maturation of the Human Immunoglobulin Heavy Chain Repertoire With Age. *Front. Immunol.* **11**, 1734 (2020). doi:10.3389/fimmu.2020.01734 Medline
32. D. F. Robbiani, C. Gaebler, F. Muecksch, J. C. C. Lorenzi, Z. Wang, A. Cho, M. Agudelo, C. O. Barnes, A. Gazumyan, S. Finkin, T. Hägglöf, T. Y. Oliveira, C. Viant, A. Hurley, H.-H. Hoffmann, K. G. Millard, R. G. Kost, M. Cipolla, K. Gordon, F. Bianchini, S. T. Chen, V. Ramos, R. Patel, J. Dizon, I. Shimeliovich, P. Mendoza, H. Hartweger, L. Nogueira, M. Pack, J. Horowitz, F. Schmidt, Y. Weisblum, E. Michailidis, A. W. Ashbrook, E. Waltari, J. E. Pak, K. E. Huey-Tubman, N. Koranda, P. R. Hoffman, A. P. West Jr., C. M. Rice, T. Hatziioannou, P. J. Bjorkman, P. D. Bieniasz, M. Caskey, M. C. Nussenzweig, Convergent antibody responses to SARS-CoV-2 in convalescent individuals. *Nature* **584**, 437–442 (2020). doi:10.1038/s41586-020-2456-9 Medline
33. Y. Cao, B. Su, X. Guo, W. Sun, Y. Deng, L. Bao, Q. Zhu, X. Zhang, Y. Zheng, C. Geng, X. Chai, R. He, X. Li, Q. Lv, H. Zhu, W. Deng, Y. Xu, Y. Wang, L. Qiao, Y. Tan, L. Song, G. Wang, X. Du, N. Gao, J. Liu, J. Xiao, X. D. Su, Z. Du, Y. Feng, C. Qin, C. Qin, R. Jin, X. S. Xie, Potent Neutralizing Antibodies against SARS-CoV-2 Identified by High-Throughput Single-Cell Sequencing of Convalescent Patients' B Cells. *Cell* **182**, 73–84.e16 (2020). doi:10.1016/j.cell.2020.05.025 Medline
34. A. Z. Wec, D. Wrapp, A. S. Herbert, D. P. Maurer, D. Haslwanter, M. Sakharkar, R. K. Jangra, M. E. Dieterle, A. Lilov, D. Huang, L. V. Tse, N. V. Johnson, C.-L. Hsieh, N. Wang, J. H. Nett, E. Champney, I. Burnina, M. Brown, S. Lin, M. Sinclair, C. Johnson, S. Pudi, R. Bortz 3rd, A. S. Wirchnianski, E. Laudermilch, C. Florez, J. M. Fels, C. M. O'Brien, B. S. Graham, D. Nemazee, D. R. Burton, R. S. Baric, J. E. Voss, K. Chandran, J. M. Dye, J. S. McLellan, L. M. Walker, Broad neutralization of SARS-related viruses by human monoclonal antibodies. *Science* **369**, 731–736 (2020). doi:10.1126/science.abc7424 Medline
35. C. Kreer, M. Zehner, T. Weber, M. S. Ercanoglu, L. Gieselmann, C. Rohde, S. Halwe, M. Korenkov, P. Schommers, K. Vanshylla, V. Di Cristanziano, H. Janicki, R. Brinker, A. Ashurov, V. Krähling, A. Kupke, H. Cohen-Dvashi, M. Koch, J. M. Eckert, S. Lederer, N. Pfeifer, T. Wolf, M. J. G. T. Vehreschild, C. Wendtner, R. Diskin, H. Gruell, S. Becker, F. Klein, Longitudinal Isolation of Potent Near-Germline SARS-CoV-2-Neutralizing Antibodies from COVID-19 Patients. *Cell* **182**, 843–854.e12 (2020). doi:10.1016/j.cell.2020.06.044 Medline
36. J. F. Scheid, H. Mouquet, N. Feldhahn, M. S. Seaman, K. Velinzon, J. Pietzsch, R. G. Ott, R. M. Anthony, H. Zebroski, A. Hurley, A. Phogat, B. Chakrabarti, Y. Li, M. Connors, F. Pereyra, B. D. Walker, H. Wardemann, D. Ho, R. T. Wyatt, J. R. Mascola, J. V. Ravetch, M. C. Nussenzweig, Broad diversity of neutralizing antibodies isolated from memory B cells in HIV-infected individuals. *Nature* **458**, 636–640 (2009). doi:10.1038/nature07930 Medline
37. M. Z. Tay, K. Wiehe, J. Pollara, Antibody-Dependent Cellular Phagocytosis in Antiviral Immune Responses. *Front. Immunol.* **10**, 332 (2019). doi:10.3389/fimmu.2019.00332 Medline
38. D. J. DiLillo, J. V. Ravetch, Fc-Receptor Interactions Regulate Both Cytotoxic and Immunomodulatory Therapeutic Antibody Effector Functions. *Cancer Immunol. Res.* **3**, 704–713 (2015). doi:10.1158/2326-6066.CIR-15-0120 Medline
39. G. Yaari, M. Uduman, S. H. Kleinstein, Quantifying selection in high-throughput Immunoglobulin sequencing data sets. *Nucleic Acids Res.* **40**, e134 (2012). doi:10.1093/nar/gks457 Medline
40. G. Yaari, J. A. Vander Heiden, M. Uduman, D. Gadala-Maria, N. Gupta, J. N. H. Stern, K. C. O'Connor, D. A. Hafler, U. Laserson, F. Vigneault, S. H. Kleinstein, Models of somatic hypermutation targeting and substitution based on synonymous mutations from high-throughput immunoglobulin sequencing data. *Front. Immunol.* **4**, 358 (2013). doi:10.3389/fimmu.2013.00358 Medline
41. J. M. Dan *et al.*, *bioRxiv*, in press (available at https://doi.org/10.1101/2020.11.15.383323).
42. P. S. Arunachalam, F. Wimmers, C. K. P. Mok, R. A. P. M. Perera, M. Scott, T. Hagan, N. Sigal, Y. Feng, L. Bristow, O. Tak-Yin Tsang, D. Wagh, J. Coller, K. L. Pellegrini, D. Kazmin, G. Alaaeddine, W. S. Leung, J. M. C. Chan, T. S. H. Chik, C. Y. C. Choi, C. Huerta, M. Paine McCullough, H. Lv, E. Anderson, S. Edupuganti, A. A. Upadhyay, S. E. Bosinger, H. T. Maecker, P. Khatri, N. Rouphael, M. Peiris, B. Pulendran, Systems biological assessment of immunity to mild versus severe COVID-19 infection in humans. *Science* **369**, 1210–1220 (2020). doi:10.1126/science.abc6261 Medline
43. A. M. Arvin, K. Fink, M. A. Schmid, A. Cathcart, R. Spreafico, C. Havenar-Daughton, A. Lanzavecchia, D. Corti, H. W. Virgin, A perspective on potential antibody-dependent enhancement of SARS-CoV-2. *Nature* **584**, 353–363 (2020). Medline
44. F. Yasui, M. Kohara, M. Kitabatake, T. Nishiwaki, H. Fujii, C. Tateno, M. Yoneda, K. Morita, K. Matsushima, S. Koyasu, C. Kai, Phagocytic cells contribute to the antibody-mediated elimination of pulmonary-infected SARS coronavirus. *Virology* **454-455**, 157–168 (2014). doi:10.1016/j.virol.2014.02.005 Medline
45. D. H. Schuurhuis, A. Ioan-Facsinay, B. Nagelkerken, J. J. van Schip, C. Sedlik, C. J. M. Melief, J. S. Verbeek, F. Ossendorp, Antigen-antibody immune complexes empower dendritic cells to efficiently prime specific CD8+ CTL responses in vivo. *J. Immunol.* **168**, 2240–2246 (2002). doi:10.4049/jimmunol.168.5.2240 Medline
46. W. Dejnirattisai, A. Jumnainsong, N. Onsirisakul, P. Fitton, S. Vasanawathana, W. Limpitikul, C. Puttikhunt, C. Edwards, T. Duangchinda, S. Supasa, K. Chawansuntati, P. Malasit, J. Mongkolsapaya, G. Screaton, Cross-reacting antibodies enhance dengue virus infection in humans. *Science* **328**, 745–748 (2010). doi:10.1126/science.1185181 Medline
47. S. J. Zost, P. Gilchuk, J. B. Case, E. Binshtein, R. E. Chen, J. P. Nkolola, A. Schäfer, J. X. Reidy, A. Trivette, R. S. Nargi, R. E. Sutton, N. Suryadevara, D. R. Martinez, L. E. Williamson, E. C. Chen, T. Jones, S. Day, L. Myers, A. O. Hassan, N. M. Kafai, E. S. Winkler, J. M. Fox, S. Shrihari, B. K. Mueller, J. Meiler, A. Chandrashekar, N. B. Mercado, J. J. Steinhardt, K. Ren, Y.-M. Loo, N. L. Kallewaard, B. T. McCune, S. P. Keeler, M. J. Holtzman, D. H. Barouch, L. E. Gralinski, R. S. Baric, L. B. Thackray, M. S. Diamond, R. H. Carnahan, J. E. Crowe Jr., Potently neutralizing and protective human antibodies against SARS-CoV-2. *Nature* **584**, 443–449 (2020). doi:10.1038/s41586-020-2548-6 Medline
48. A. W. D. Edridge, J. Kaczorowska, A. C. R. Hoste, M. Bakker, M. Klein, K. Loens, M. F. Jebbink, A. Matser, C. M. Kinsella, P. Rueda, M. Ieven, H. Goossens, M. Prins, P. Sastre, M. Deijs, L. van der Hoek, Seasonal coronavirus protective immunity is short-lasting. *Nat. Med.* **26**, 1691–1693 (2020). doi:10.1038/s41591-020-1083-1 Medline
49. E. G. Severance, I. Bossis, F. B. Dickerson, C. R. Stallings, A. E. Origoni, A. Sullens, R. H. Yolken, R. P. Viscidi, Development of a nucleocapsid-based human coronavirus immunoassay and estimates of individuals exposed to coronavirus in a U.S. metropolitan population. *Clin. Vaccine Immunol.* **15**, 1805–1810 (2008). doi:10.1128/CVI.00124-08 Medline
50. R. Dijkman, M. F. Jebbink, N. B. El Idrissi, K. Pyrc, M. A. Müller, T. W. Kuijpers, H. L. Zaaijer, L. van der Hoek, Human coronavirus NL63 and 229E seroconversion in children. *J. Clin. Microbiol.* **46**, 2368–2373 (2008). doi:10.1128/JCM.00533-08 Medline
51. A. T. Huang, B. Garcia-Carreras, M. D. T. Hitchings, B. Yang, L. C. Katzelnick, S. M. Rattigan, B. A. Borgert, C. A. Moreno, B. D. Solomon, L. Trimmer-Smith, V. Etienne, I. Rodriguez-Barraquer, J. Lessler, H. Salje, D. S. Burke, A. Wesolowski, D. A. T. Cummings, A systematic review of antibody mediated immunity to coronaviruses: Kinetics, correlates of protection, and association with severity. *Nat. Commun.* **11**, 4704 (2020). doi:10.1038/s41467-020-18450-4 Medline
52. P. Daszak, K. J. Olival, H. Li, A strategy to prevent future epidemics similar to the 2019-nCoV outbreak. *Biosaf. Heal.* **2**, 6–8 (2020). doi:10.1016/j.bsheal.2020.01.003 Medline
53. J. S. Lavine, O. N. Bjornstad, R. Antia, Immunological characteristics govern the transition of COVID-19 to endemicity. *Science* **371**, 741–745 (2021). doi:10.1126/science.abe6522 Medline
54. H. B. Larman, U. Laserson, L. Querol, K. Verhaeghen, N. L. Solimini, G. J. Xu, P. L. Klarenbeek, G. M. Church, D. A. Hafler, R. M. Plenge, P. A. Nigrovic, P. L. De Jager, I. Weets, G. A. Martens, K. C. O'Connor, S. J. Elledge, PhIP-Seq characterization of autoantibodies from patients with multiple sclerosis, type 1 diabetes and rheumatoid arthritis. *J. Autoimmun.* **43**, 1–9 (2013). doi:10.1016/j.jaut.2013.01.013 Medline
55. L. Premkumar, B. Segovia-Chumbez, R. Jadi, D. R. Martinez, R. Raut, A. Markmann, C. Cornaby, L. Bartelt, S. Weiss, Y. Park, C. E. Edwards, E. Weimer, E. M. Scherer, N. Rouphael, S. Edupuganti, D. Weiskopf, L. V. Tse, Y. J. Hou, D. Margolis, A. Sette, M. H. Collins, J. Schmitz, R. S. Baric, A. M. de Silva, The receptor binding domain of the viral spike protein is an immunodominant and highly specific target of antibodies in SARS-CoV-2 patients. *Sci. Immunol.* **5**, 1–10 (2020).

doi:10.1126/sciimmunol.abc8413 Medline
56. M. Yuan, N. C. Wu, X. Zhu, C. D. Lee, R. T. Y. So, H. Lv, C. K. P. Mok, I. A. Wilson, A highly conserved cryptic epitope in the receptor binding domains of SARS-CoV-2 and SARS-CoV. *Science* **368**, 630–633 (2020). doi:10.1126/science.abb7269 Medline
57. Y. Watanabe, J. D. Allen, D. Wrapp, J. S. Mclellan, M. Crispin, Site-specific glycan analysis of the SARS-CoV-2 spike. **9983**, 1–9 (2020).
58. J. Zhu, H. B. Larman, G. Gao, R. Somwar, Z. Zhang, U. Laserson, A. Ciccia, N. Pavlova, G. Church, W. Zhang, S. Kesari, S. J. Elledge, Protein interaction discovery using parallel analysis of translated ORFs (PLATO). *Nat. Biotechnol.* **31**, 331–334 (2013). doi:10.1038/nbt.2539 Medline
59. E. P. Scully, J. Haverfield, R. L. Ursin, C. Tannenbaum, S. L. Klein, Considering how biological sex impacts immune responses and COVID-19 outcomes. *Nat. Rev. Immunol.* **20**, 442–447 (2020). doi:10.1038/s41577-020-0348-8 Medline
60. E. J. Márquez, J. Trowbridge, G. A. Kuchel, J. Banchereau, D. Ucar, The lethal sex gap: COVID-19. *Immun. Ageing* **17**, 13 (2020). doi:10.1186/s12979-020-00183-z Medline
61. T. Takahashi, M. K. Ellingson, P. Wong, B. Israelow, C. Lucas, J. Klein, J. Silva, T. Mao, J. E. Oh, M. Tokuyama, P. Lu, A. Venkataraman, A. Park, F. Liu, A. Meir, J. Sun, E. Y. Wang, A. Casanovas-Massana, A. L. Wyllie, C. B. F. Vogels, R. Earnest, S. Lapidus, I. M. Ott, A. J. Moore, A. Shaw, J. B. Fournier, C. D. Odio, S. Farhadian, C. Dela Cruz, N. D. Grubaugh, W. L. Schulz, A. M. Ring, A. I. Ko, S. B. Omer, A. Iwasaki; Yale IMPACT Research Team, Sex differences in immune responses that underlie COVID-19 disease outcomes. *Nature* **588**, 315–320 (2020). doi:10.1038/s41586-020-2700-3 Medline
62. M. J. Bunders, M. Altfeld, Implications of sex differences in immunity for SARS-CoV-2 pathogenesis and design of therapeutic interventions. *Immunity* **53**, 487–495 (2020). doi:10.1016/j.immuni.2020.08.003 Medline
63. X. Yang, X. Chen, G. Bian, J. Tu, Y. Xing, Y. Wang, Z. Chen, Proteolytic processing, deubiquitinase and interferon antagonist activities of Middle East respiratory syndrome coronavirus papain-like protease. *J. Gen. Virol.* **95**, 614–626 (2014). doi:10.1099/vir.0.059014-0 Medline
64. E. J. Snijder, E. Decroly, J. Ziebuhr, The Nonstructural Proteins Directing Coronavirus RNA Synthesis and Processing. *Adv. Virus Res.* **96**, 59–126 (2016). doi:10.1016/bs.aivir.2016.08.008 Medline
65. B. Forsström, B. B. Axnäs, J. Rockberg, H. Danielsson, A. Bohlin, M. Uhlen, Dissecting antibodies with regards to linear and conformational epitopes. *PLOS ONE* **10**, e0121673 (2015). doi:10.1371/journal.pone.0121673 Medline
66. Buitinck *et al.* API design for machine learning software: experiences from the scikit-learn, (2013) ECML PKDD Workshop: Languages for Data Mining and Machine Learning 108-122.
67. N. T. Gupta, J. A. Vander Heiden, M. Uduman, D. Gadala-Maria, G. Yaari, S. H. Kleinstein, Change-O: A toolkit for analyzing large-scale B cell immunoglobulin repertoire sequencing data. *Bioinformatics* **31**, 3356–3358 (2015). doi:10.1093/bioinformatics/btv359 Medline
68. D. Wrapp, N. Wang, K. S. Corbett, J. A. Goldsmith, C.-L. Hsieh, O. Abiona, B. S. Graham, J. S. McLellan, Cryo-EM structure of the 2019-nCoV spike in the prefusion conformation. *Science* **367**, 1260–1263 (2020). doi:10.1126/science.abb2507 Medline
69. Cubuk *et al*., (2020) The SARS-CoV-2 nucleocapsid protein is dynamic, disordered, and phase separates with RNA, preprint, https://www.biorxiv.org/content/10.1101/2020.06.17.158121v1
70. T. Chen, C. Guestrin, in *Proceedings of the 22nd ACM SIGKDD International Conference on Knowledge Discovery and Data Mining* (ACM, New York, NY, USA, 2016; https://dl.acm.org/doi/10.1145/2939672.2939785), pp. 785–794.
71. S. Kumar, G. Stecher, M. Li, C. Knyaz, K. Tamura, MEGA X: Molecular Evolutionary Genetics Analysis across Computing Platforms. *Mol. Biol. Evol.* **35**, 1547–1549 (2018). doi:10.1093/molbev/msy096 Medline
72. Klompus *et al*., (2020) Cross-reactive antibody responses against SARS-CoV-2 and seasonal common cold coronaviruses, preprint medRxiv, version 2 (September 9, 2020)
73. T. F. Rogers, F. Zhao, D. Huang, N. Beutler, A. Burns, W. T. He, O. Limbo, C. Smith, G. Song, J. Woehl, L. Yang, R. K. Abbott, S. Callaghan, E. Garcia, J. Hurtado, M. Parren, L. Peng, S. Ramirez, J. Ricketts, M. J. Ricciardi, S. A. Rawlings, N. C. Wu, M. Yuan, D. M. Smith, D. Nemazee, J. R. Teijaro, J. E. Voss, I. A. Wilson, R. Andrabi, B. Briney, E. Landais, D. Sok, J. G. Jardine, D. R. Burton, Isolation of potent SARS-CoV-2 neutralizing antibodies and protection from disease in a small animal model. *Science* **369**, 956–963 (2020). doi:10.1126/science.abc7520 Medline

Acknowledgments: E.S.'s COVID-19 research is supported by the Seerave Foundation and the Israeli Ministry of Health (3-16933). T.V. is supported by an Erwin Schrödinger fellowship (J 4256) from the Austrian Science Fund (FWF). Z.S. is supported by Israel Science Foundation (ISF) KillCorona (3877019) and by Miel de Botton and the Corona Response Fund at the Weizmann Institute of Science. R.D.'s COVID-19 research is supported by the Ernst I Ascher Foundation, the Ben B. & Joyce E. Eisenberg Foundation, and by a research grant from Natan Sharansky. G.Y. is supported by the Israeli Ministry of Science grant number 3-16909. **Author Contributions:** T.V. and S.K. conceived the project and designed the library. S.L. designed and implemented the coding of the library. T.V. and S.K. performed the PhIP-Seq experiments. S.L. designed and implemented the computational pipeline. S.L., I.N.K., and A.G. performed high-throughput data analysis. T.V. analyzed additional data and wrote the manuscript. E.Shinar performed sample acquisition. E.Segal and A.W. conceived and directed the project. T.V., S.K., S.L., I.N.K., A.G., E.Shinar, A.W., and E.Segal reviewed and edited the manuscript. R.M. sequenced, generated and did functional testing of patient-derived antibodies. L.S.M. sorted patient-derived cells. N.N. produced and did functional testing of antibodies. A.P. and G.Y. did immunoglobulin sequence analyses. L.M. did structural predictions. S.K.B.T. collected patient blood. H.C.D. did antibody neutralization assays. R.G. produced peptides. N.L. supervised peptide synthesis. R.N. supervised antibody neutralization assays. Z.S. supervised antibody sequencing, production and functional testing, wrote the manuscript, and acquired funding. **Competing Interests:** The Weizmann Institute of Science and Yeda Research & Development Co. Ltd. has filed a provisional patent application in connection with monoclonal antibodies presented in this work on which Z.S., R.M., L.S.B., S.B.K., L.M. and N.N. are inventors (Israel Patent Application No. 280340). The other authors declare no competing interests. **Data and Materials Availability:** The majority of data generated or analyzed during this study are included with the manuscript and the supplementary materials. Custom code used for analyzing the PhIP-Seq data are publicly available: https://github.com/erans99/PhageIPSeq_CoVs. Additional datasets (and code) generated and analyzed during the current study are available from the corresponding authors on reasonable request. This work is licensed under a Creative Commons Attribution 4.0 International (CC BY 4.0) license, which permits unrestricted use, distribution, and reproduction in any medium, provided the original work is properly cited. To view a copy of this license, visit https://creativecommons.org/licenses/by/4.0/. This license does not apply to figures/photos/artwork or other content included in the article that is credited to a third party; obtain authorization from the rights holder before using such material.

Submitted 27 September 2020
Accepted 27 July 2021
Published First Release 29 July 2021
10.1126/sciimmunol.abe9950

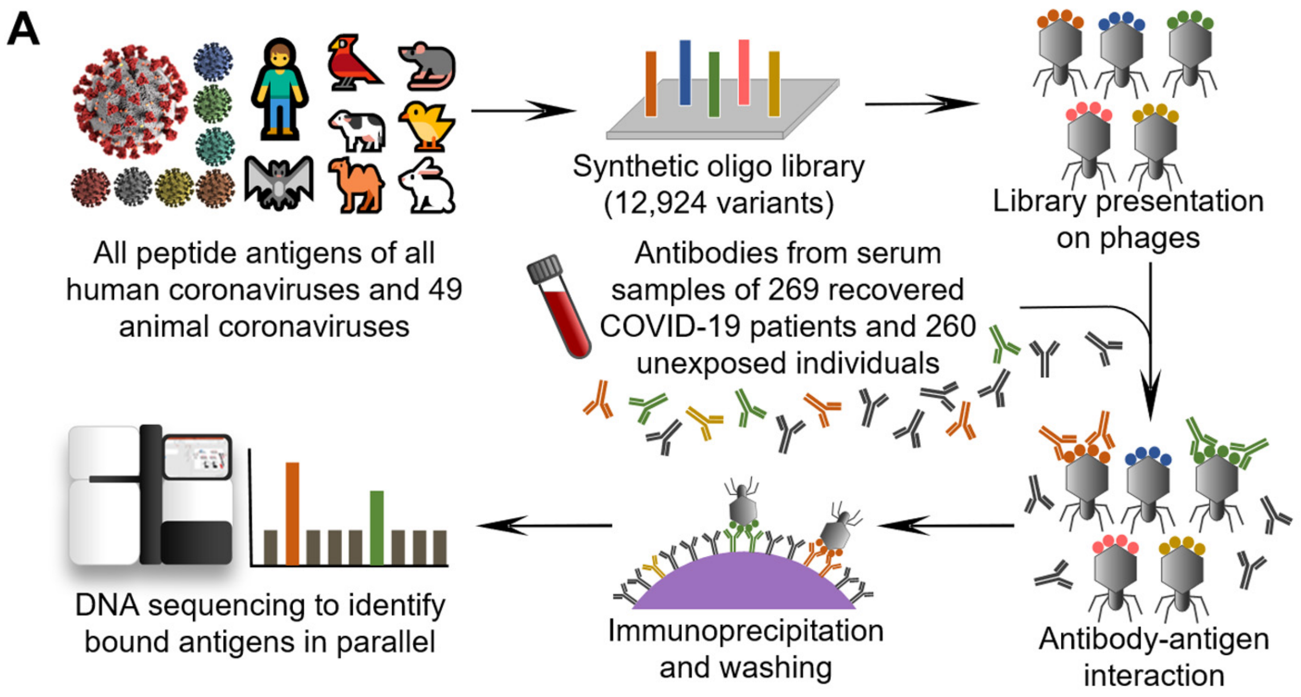

B

			Proteins	Peptides
Human COVs	SARS-CoV-2		29	230
	SARS-CoV		27	229
	MERS-CoV		30	353
	Seasonal hCOVs	OC43	23	243
		HKU1	22	228
		NL63	20	207
		229E	21	203
Animal CoVs	Bats (19 CoVs)		n.a.	4,067
	Other mammals (19 CoVs))		n.a.	4,135
	Birds (11 CoVs)		n.a.	2,166
SARS-CoV-2 variants			n.a.	988
Controls			13	268
Complete CoV library			n.a.	13,192*

C

		Serum samples
**Recovered COVID-19 patients**		
Number of samples		269
Sex	Male / Female	74 / 195
Age	Range	17 to 73
	Average ± SD	36.4 ± 13.0
Collection dates		Apr. 2020 to May 2020
**Unexposed individuals**		
Number of samples		260
Sex	Male / Female	80/180
Age	Range	17 to 70
	Average ± SD	34.7 ± 11.0
Collection dates		Jul. 2013 to Mar. 2016

Fig. 1. A phage displayed antigen library (a) of 12,924 human and animal coronavirus peptide antigens (b) was applied to serum samples of 260 unexposed individuals and 269 recovered COVID-19 patients (c). The numbers of hCoV proteins per strain in panel b includes polyproteins being split into separate proteins. The listed MERS-CoV peptides include also the variant betacoronavirus England 1. See Data file 1 for a detailed list of all strains including accession numbers. The SARS-CoV-2 variants listed include also the reference SARS-CoV-2 peptides. The illustration of the SARS-CoV-2 virion is reproduced from CDC PHIL #23312 released as public domain (CDC/ Alissa Eckert, MSMI; Dan Higgins, MAMS). *Number of unique peptides (a few are shared between groups).

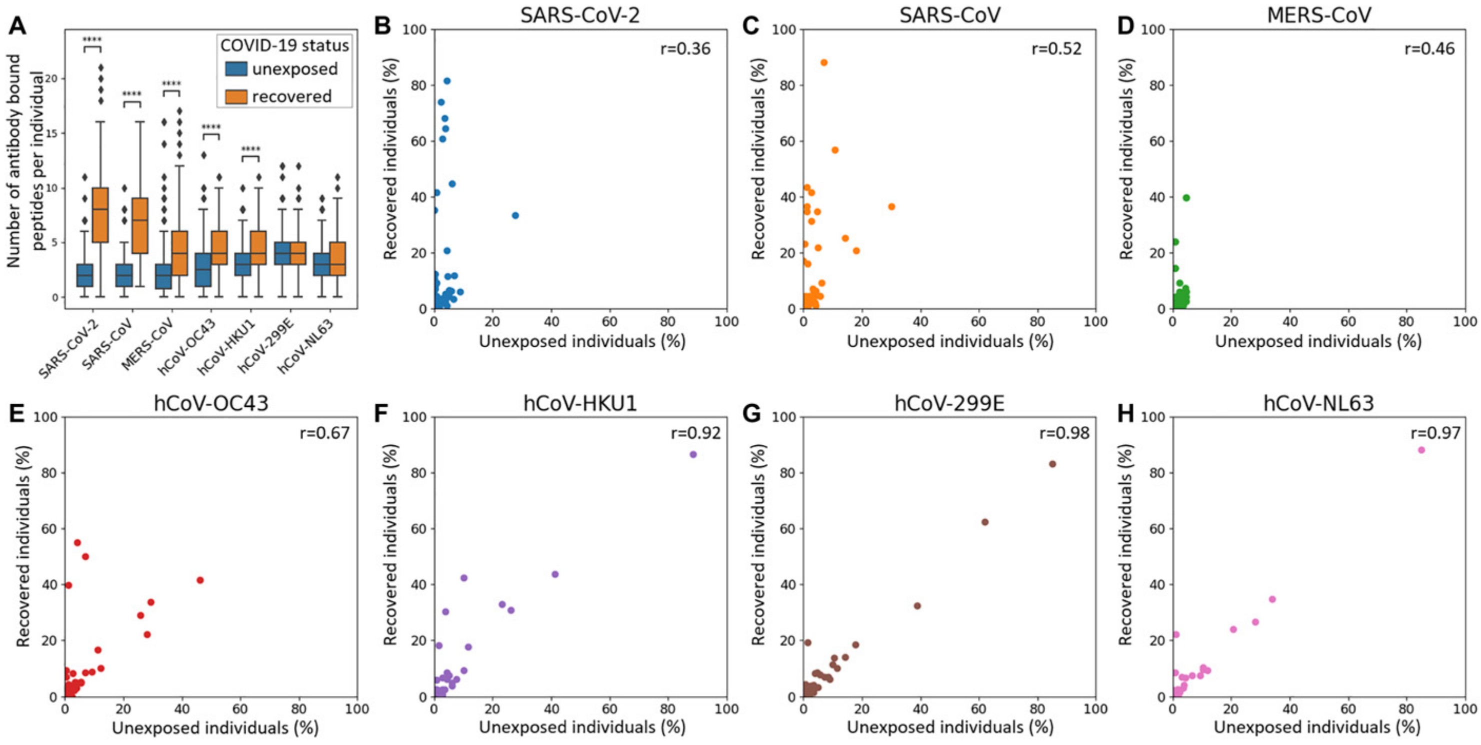

Fig. 2. Detection of a high serum prevalence of seasonal hCoVs, interindividual variability of antibody repertoires against SARS-CoV-2, and cross-reactive antibody responses against seasonal hCoVs upon SARS-CoV-2 infection. **a** The numbers of antibody bound peptide antigens of hCoVs per individual are compared between unexposed individuals (n=260) and recovered COVID-19 patients (n=269). For SARS-CoV-2 only peptides of the reference genome are included, while variants are not shown (listed in Data file S3). The MERS-CoV peptides shown include also the variant Betacoronavirus England 1. The center line shows the median; box limits indicate the 25th and 75th percentiles as determined by Seaborn software; whiskers extend 1.5 times the interquartile range from the 25th and 75th percentiles, outliers are represented by dots. Significance between the groups was calculated with the Mann-Whitney test (**** indicates p-values $<10^{-4}$, see Fig. S5 for additional p-value criteria). **b-h**, Antibody responses in unexposed individuals and recovered COVID-19 patients shown for each hCoV strain separately. Each dot represents a peptide with its abundance in the respective cohort plotted on the x/y axes. The correlation coefficient (Pearson r) between the groups of unexposed individuals and recovered individuals is displayed in the top right corner of each panel.

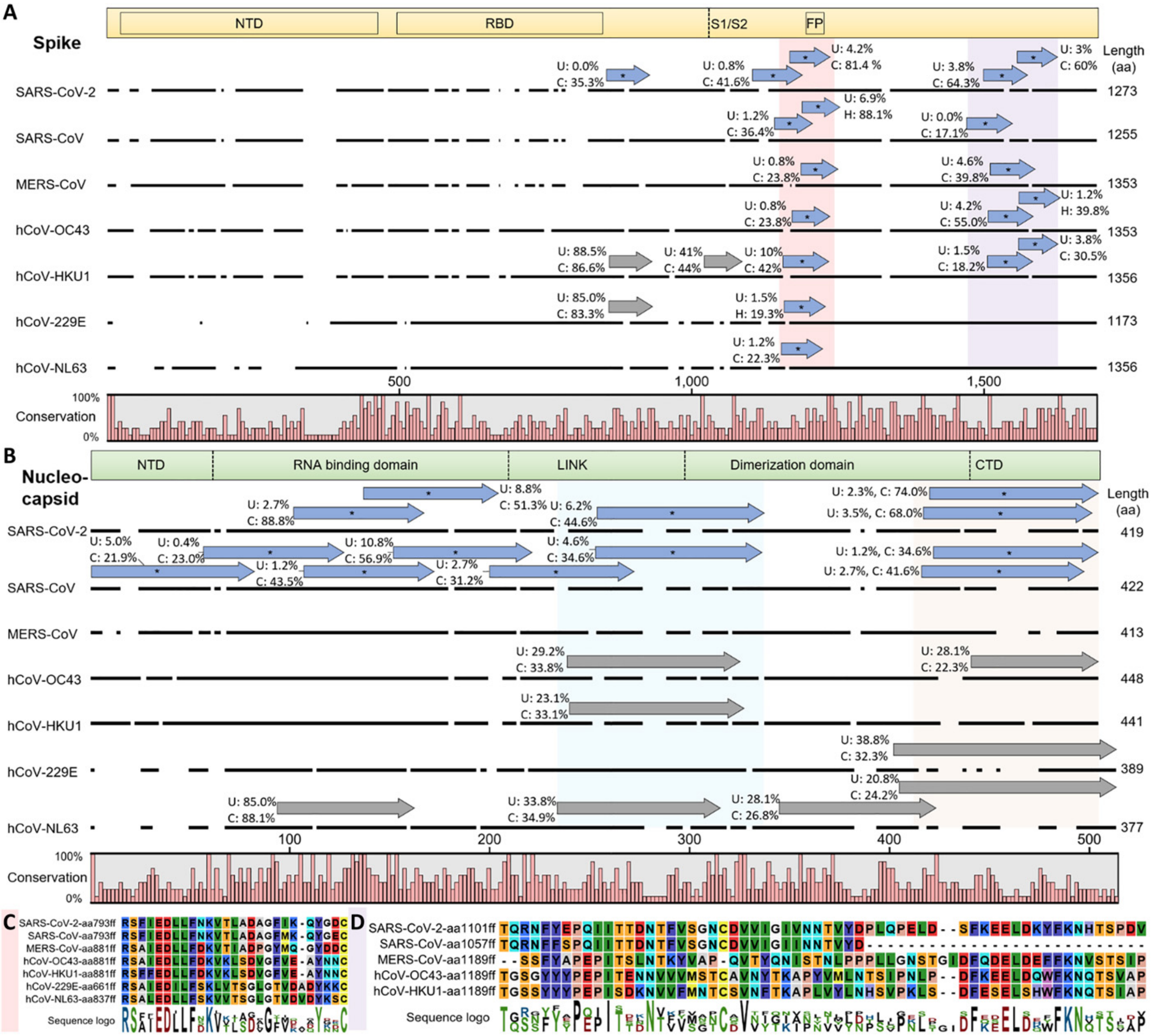

Fig. 3. **Cross-reactive and selective antibody binding of SARS-CoV-2 peptides and other hCoVs clusters in similar regions of the spike (a) and nucleocapsid (b) protein, with shared motifs of bound spike peptides highlighted (c,d).** **a,b** Alignments of S- and N-proteins of all hCoVs, the dark line next to the strain identifier represents the protein sequence indicating gaps in the consensus alignment. Peptides bound at significantly different percentages (Chi-squared/Kolmogorov–Smirnov tests and passing FDR correction, see Data file S3) between unexposed ('U') individuals or recovered COVID-19 patients ('C') are shown as blue arrows above the corresponding protein sequence and marked with an asterisk. The abundance of binding in 'U' and 'C' is indicated as percentages written next to the peptides. Grey arrows indicate similar recognition in >20% of unexposed individuals and COVID-19 patients. For SARS-CoV-2 only peptides of the reference genome are included (variants not shown but listed in Data file S3). The domain structure on top of each panel is based on SARS-CoV-2 S- (*68*)/N- (*69*) proteins, positions in other hCoVs shift along the alignment. Due to the different lengths of S- and N- proteins, the two panels are not drawn at the same scale. **c,d** Motifs from alignments of bound spike peptides in the regions marked in light red (c) and light purple (d) in panel a. See Fig. S 3a,b for full alignments of the peptides and details. Alignments of the nucleocapsid regions marked in light blue and light orange are due to space constraints shown in Fig. S3c,d.

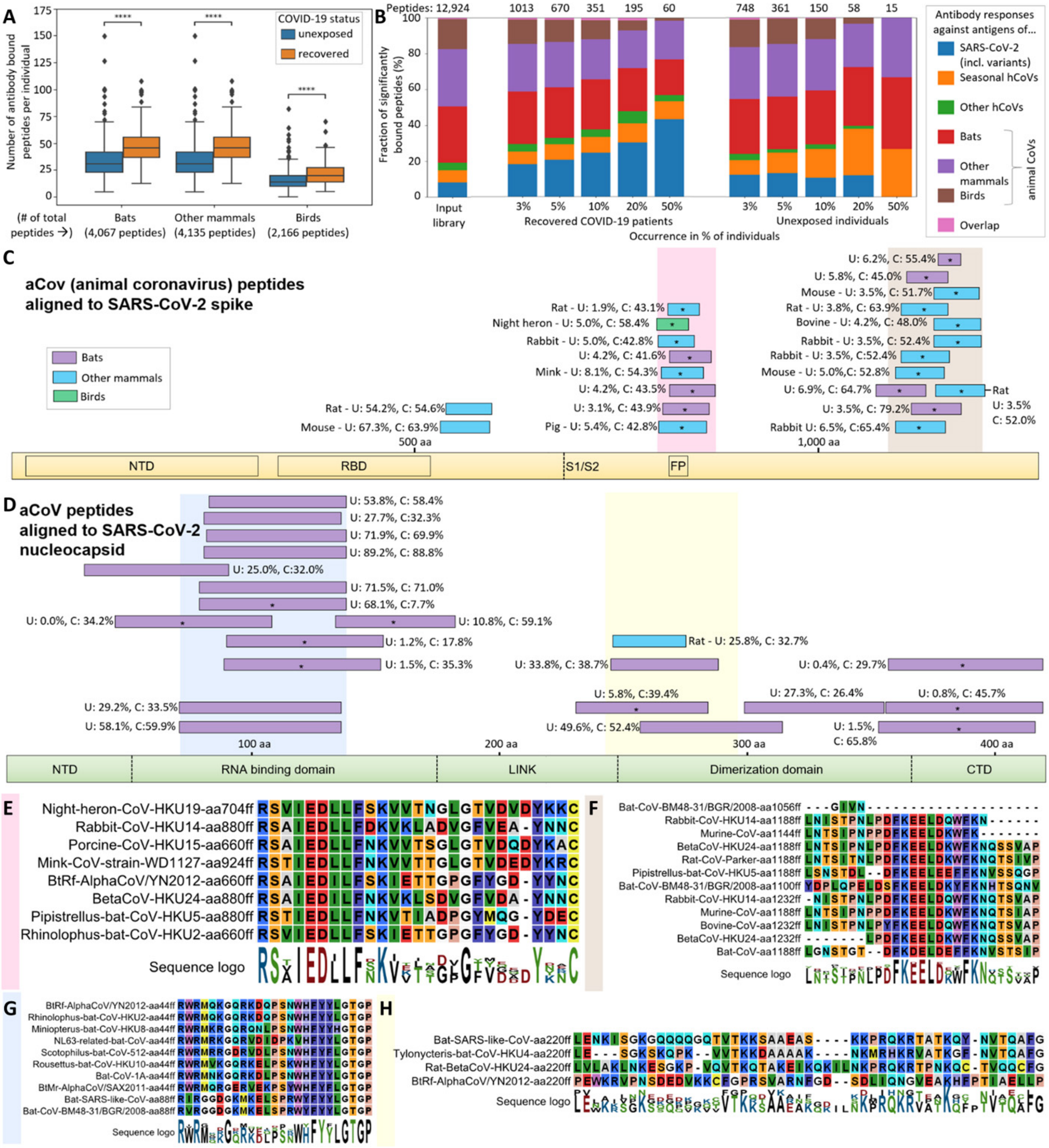
A
COVID-19 status
unexposed
recovered
Number of antibody bound peptides per individual
175
150
125
100
75
50
25
0
****
****
****
(# of total peptides →)
Bats (4,067 peptides)
Other mammals (4,135 peptides)
Birds (2,166 peptides)
B
Peptides: 12,924
1013
670
351
195
60
748
361
150
58
15
Fraction of significantly bound peptides (%)
100
80
60
40
20
0
Input library
3%
5%
10%
20%
50%
Recovered COVID-19 patients
Unexposed individuals
Occurrence in % of individuals
Antibody responses against antigens of...
SARS-CoV-2 (incl. variants)
Seasonal hCoVs
Other hCoVs
Bats
Other mammals
Birds
animal CoVs
Overlap
C
aCov (animal coronavirus) peptides aligned to SARS-CoV-2 spike
Bats
Other mammals
Birds
Rat - U: 54.2%, C: 54.6%
Mouse - U: 67.3%, C: 63.9%
Rat - U: 1.9%, C: 43.1%
Night heron - U: 5.0%, C: 58.4%
Rabbit - U: 5.0%, C:42.8%
U: 4.2%, C: 41.6%
Mink - U: 8.1%, C: 54.3%
U: 4.2%, C: 43.5%
U: 3.1%, C: 43.9%
Pig - U: 5.4%, C: 42.8%
U: 6.2%, C: 55.4%
U: 5.8%, C: 45.0%
Mouse - U: 3.5%, C: 51.7%
Rat - U: 3.8%, C: 63.9%
Bovine - U: 4.2%, C: 48.0%
Rabbit - U: 3.5%, C: 52.4%
Rabbit - U: 3.5%, C:52.4%
Mouse - U: 5.0%,C: 52.8%
U: 6.9%, C: 64.7%
Rat U: 3.5% C: 52.0%
U: 3.5%, C: 79.2%
Rabbit U: 6.5%, C:65.4%
500 aa
1,000 aa
NTD
RBD
S1/S2
FP
D
aCoV peptides aligned to SARS-CoV-2 nucleocapsid
U: 53.8%, C: 58.4%
U: 27.7%, C:32.3%
U: 71.9%, C: 69.9%
U: 89.2%, C: 88.8%
U: 25.0%, C:32.0%
U: 71.5%, C: 71.0%
U: 68.1%, C:7.7%
U: 0.0%, C: 34.2%
U: 10.8%, C: 59.1%
U: 1.2%, C: 17.8%
Rat - U: 25.8%, C: 32.7%
U: 1.5%, C: 35.3%
U: 33.8%, C: 38.7%
U: 0.4%, C: 29.7%
U: 5.8%, C:39.4%
U: 27.3%, C: 26.4%
U: 0.8%, C: 45.7%
U: 29.2%, C: 33.5%
U: 58.1%, C:59.9%
U: 49.6%, C: 52.4%
U: 1.5%, C: 65.8%
100 aa
200 aa
300 aa
400 aa
NTD
RNA binding domain
LINK
Dimerization domain
CTD
E
Night-heron-CoV-HKU19-aa704ff RSVIEDLLFSKVVTNGLGTVDVDYKKC
Rabbit-CoV-HKU14-aa880ff RSAIEDLLFDKVKLADVGFVEA-YNNC
Porcine-CoV-HKU15-aa660ff RSAIEDLLFNKVVTSGLGTVDQDYKAC
Mink-CoV-strain-WD1127-aa924ff RSTIEDLLFNKVVTTGLGTVDEDYKRC
BtRf-AlphaCoV/YN2012-aa660ff RSAIEDILFSKIETTGPGFYGD-YYNC
BetaCoV-HKU24-aa880ff RSAIEDILFNKVKLSDVGFVDA-YNNC
Pipistrellus-bat-CoV-HKU5-aa880ff RSTIEDLLFNKVTIADPGYMQG-YDEC
Rhinolophus-bat-CoV-HKU2-aa660ff RSVIEDLLFSKIETTGPGFYGD-YYNC
Sequence logo
F
Bat-CoV-BM48-31/BGR/2008-aa1056ff
Rabbit-CoV-HKU14-aa1188ff
Murine-CoV-aa1144ff
BetaCoV-HKU24-aa1188ff
Rat-CoV-Parker-aa1188ff
Pipistrellus-bat-CoV-HKU5-aa1188ff
Bat-CoV-BM48-31/BGR/2008-aa1100ff
Rabbit-CoV-HKU14-aa1232ff
Murine-CoV-aa1188ff
Bovine-CoV-aa1232ff
BetaCoV-HKU24-aa1232ff
Bat-CoV-aa1188ff
Sequence logo
G
BtRf-AlphaCoV/YN2012-aa44ff
Rhinolophus-bat-CoV-HKU2-aa44ff
Miniopterus-bat-CoV-HKU8-aa44ff
NL63-related-bat-CoV-aa44ff
Scotophilus-bat-CoV-512-aa44ff
Rousettus-bat-CoV-HKU10-aa44ff
Bat-CoV-1A-aa44ff
BtMr-AlphaCoV/SAX2011-aa44ff
Bat-SARS-like-CoV-aa88ff
Bat-CoV-BM48-31/BGR/2008-aa88ff
Sequence logo
H
Bat-SARS-like-CoV-aa220ff
Tylonycteris-bat-CoV-HKU4-aa220ff
Rat-BetaCoV-HKU24-aa220ff
BtRf-AlphaCoV/YN2012-aa220ff
Sequence logo

Fig. 4. Cross-reactivity of human serum antibodies extends toward animal coronaviruses (aCoVs) in both SARS-CoV-2 recovered patients and unexposed individuals (a,b). Alignments of bound aCoV peptides to SARS-CoV-2 cluster in similar regions of the spike (c) and nucleocapsid (d) protein, with shared motifs of bound spike (e,f) and nucleocapsid (g,h) peptides highlighted. a Antibody responses against antigens of 49 animal coronaviruses are summarized for the groups indicated (separate data for each strain is shown in Fig. S5). In all panels of this figure antibody binding data of the full set of 269 recovered COVID-19 patients and 260 unexposed individuals is shown. '# of total peptides' refers to number peptides included for each strain within the library (Fig. 1b), whereas the number of antibody bound peptides per individual is plotted for each group on the y-axis. The center line shows the median; box limits indicate the 25th and 75th percentiles as determined by Seaborn software; whiskers extend 1.5 times the interquartile range from the 25th and 75th percentiles, outliers are represented by dots. Significance between the groups was calculated with the Mann-Whitney test (**** indicates p-values $<10^{-4}$, see **Fig. S5** for additional p-value criteria). **b** Ratios of hCoVs and aCoVs antigens bound at different frequencies in recovered COVID-19 patients and unexposed individuals. The input library is shown as control representing the library content before testing for antibody binding. The numbers on top of the panel indicate the absolute number of peptides per group (as each bar shows a relative distribution between the groups). 'Other hCoVs' includes SARS-CoV and MERS-CoV. 'Overlap' refers to a few identical peptides of multiple strains that cannot be assigned to a single group. **c,d** Alignments of S- and N-peptides of aCoVs to SARS-CoV-2 S- (c) and N-proteins (d). The peptides were aligned using the BLAST algorithm in standard parameters and only aligned regions of the peptides are shown. Peptides bound at significantly different percentages (Chi-squared/ Kolmogorov–Smirnov test and passing FDR correction, see Data file S3) between unexposed ('U') individuals or recovered COVID-19 patients ('C') are shown are marked with an asterisk. The abundance of binding in 'U' and 'C' is indicated as percentages written next to the peptides. Non significantly scored peptides are only shown if being bound in >20% of unexposed individuals and COVID-19 patients. Due to the large number of significantly bound aCoV S-peptides, only peptides being bound in >40% of recovered individuals are shown while a full list is provided in Data file S3 (including BLAST alignments). The S/N-protein domain structure at the bottom of each panel as outlined in Fig. 3. Due to the different lengths of S- and N- proteins, the two panels are not drawn at the same scale. **e-h** Motifs from alignments of bound aCoV S-peptides in the regions marked in light red (e) and light brown (f) in panel c and bound aCoV N-peptides in the regions marked in light blue (g) and light yellow (h) in panel d. See Fig. S7 for full alignments of the peptides and details.

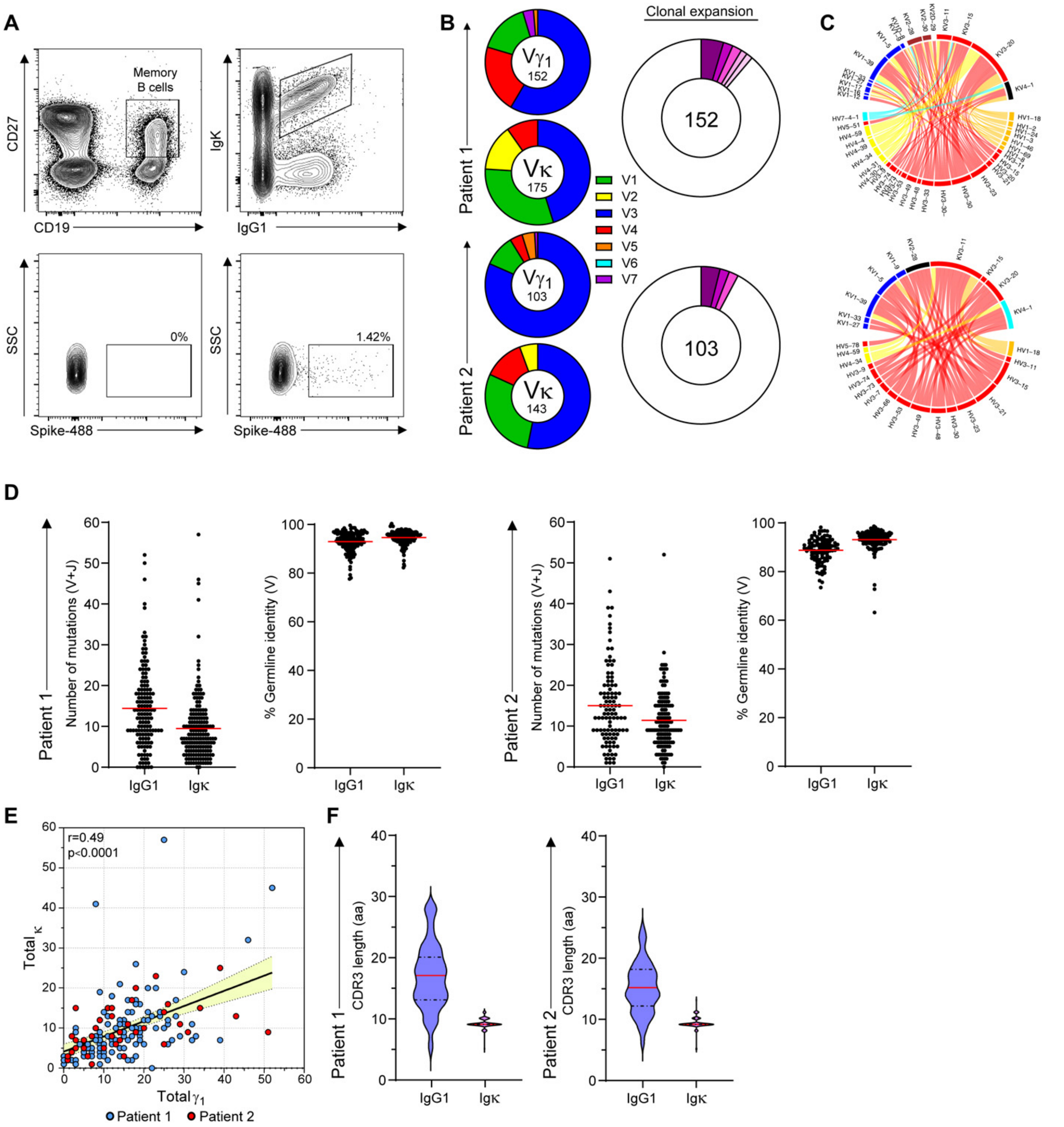

A
Memory B cells
CD27
CD19
IgK
IgG1
SSC
Spike-488
0%
1.42%
B
Patient 1
Patient 2
Vγ1
152
Vκ
175
103
143
V1
V2
V3
V4
V5
V6
V7
Clonal expansion
C
D
Number of mutations (V+J)
% Germline identity (V)
IgG1
Igκ
E
r=0.49
p<0.0001
Totalκ
Totalγ1
Patient 1
Patient 2
F
CDR3 length (aa)

Fig. 5. Anti SARS-CoV-2 antibodies by single B cell immunoglobulin sequencing. **a** Gating strategy for spike-specific memory B cells sorting (CD19$^+$, CC27$^+$, IgG1$^+$, Igκ$^+$ and Spike-binding). **b** Pie charts depicting the distribution of V (variable) genes in Ighγ1, top and Igκ of spike-specific B cells (left) and clonal expansion of spike reactive B cells (right). Each colored slice represents a unique clone. Singleton sequences are shown in white. **c** Circos plots showing coupled heavy and light chain sequences of the sorted cells. **d** The number of mutations identified in the V and J genes of heavy and light immunoglobulin chains and the percent germline identity of their V genes. **e** Correlation between the mutational load in the Ighγ1 vs. Igκ chains. Each dot represents a sequenced antibody heavy or light chain. Pearson's correlation test was used for coefficient (r) and p-value. **f** CDR3 amino acid lengths of Ighγ1 and Igκ.

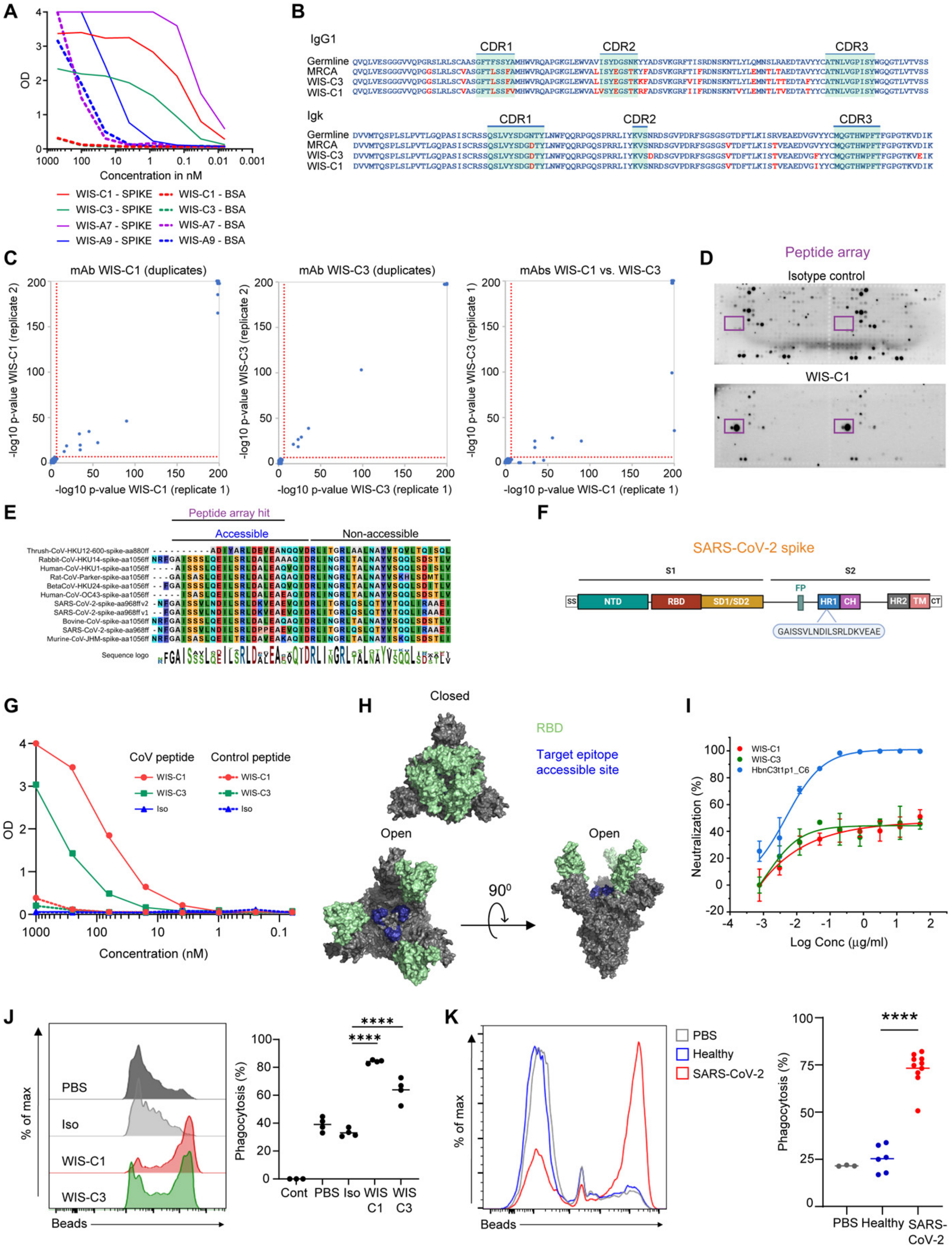
A
B
C
D
E
F
G
H
I
J
K
IgG1
Igk
CDR1
CDR2
CDR3
Germline
MRCA
WIS-C3
WIS-C1
Concentration in nM
WIS-C1 - SPIKE
WIS-C1 - BSA
WIS-C3 - SPIKE
WIS-C3 - BSA
WIS-A7 - SPIKE
WIS-A7 - BSA
WIS-A9 - SPIKE
WIS-A9 - BSA
mAb WIS-C1 (duplicates)
mAb WIS-C3 (duplicates)
mAbs WIS-C1 vs. WIS-C3
-log10 p-value WIS-C1 (replicate 1)
-log10 p-value WIS-C1 (replicate 2)
-log10 p-value WIS-C3 (replicate 1)
-log10 p-value WIS-C3 (replicate 2)
Peptide array
Isotype control
WIS-C1
Peptide array hit
Accessible
Non-accessible
Sequence logo
SARS-CoV-2 spike
S1
S2
SS
NTD
RBD
SD1/SD2
FP
HR1
CH
HR2
TM
CT
GAISSVLNDILSRLDKVEAE
CoV peptide
Control peptide
Iso
Concentration (nM)
Closed
Open
RBD
Target epitope accessible site
90⁰
HbnC3t1p1_C6
Neutralization (%)
Log Conc (µg/ml)
PBS
Iso
WIS-C1
WIS-C3
% of max
Beads
Phagocytosis (%)
Cont
Healthy
SARS-CoV-2
****

Fig. 6. Patient-derived, pan-specific monoclonal antibodies. **a** Monoclonal antibody reactivity to spike protein and BSA at different concentrations by ELISA. **b** Comparison between amino acid sequences of WIS-C1 and WIS-C3 to their germline and most recent common ancestor (MRCA) configurations. Mutated amino acids appear in red. CDR, complementarity-determining region. **c** PhIP-Seq based identification of targeted peptides within the CoV-antigen library of WIS-C1 (left), WIS-C3 (middle) and comparison of the peptides bound by the two antibodies (right). The two mAbs were mixed with the phage displayed antigen library and processed in the same way as serum samples. Reaction mixtures were set up in duplicates and significantly bound peptides are marked (threshold indicated by dotted red line). The maximum p-values computed are cut off at -log10 200. See Fig. S8 for a list of significantly bound peptides. **d** Epitope mapping using spike peptide array of isotype control antibody (top) and the WIS-C1 antibody (bottom). WIS-C1 epitope target is shown in the purple rectangle. **e** Motif analysis of the target epitope of WIS-C1 and WIS-C3, obtained by multiple sequence alignment of the top 6 hCoVs and top 9 aCoVs PhIP-Seq hits. See Fig. S8 for full alignments of the peptides and details. **f** Linear SARS-CoV-2 spike schematics depicting the location of the target epitope on the viral protein. **g** Binding of WIS-C1 and WIS-C3 to the target or control peptide by ELISA. **h** WIS-C1 and WIS-C3 target epitope projected on the crystal structure of SARS-CoV-2 spike in the closed (top) and open (bottom) confirmations. **i** Neutralization activity of pseudo-viruses by WIS-C1 and WIS-C3 compared with HbnC3t1p1_C6, as a positive control. Representative of two independent experiments, error bars indicate standard deviations of technical repeats. **(j,k)** FACS plots depicting the uptake of fluorescent spike-coated spheres by THP-1 monocytes in the presence of (j) monoclonal antibodies or (k) patient sera. (j) four independent experiments; ****P < 0.0001, one-way ANOVA or (k) a representative of 2 independent experiments; ****P < 0.0001, two-tailed Student's *t* test.

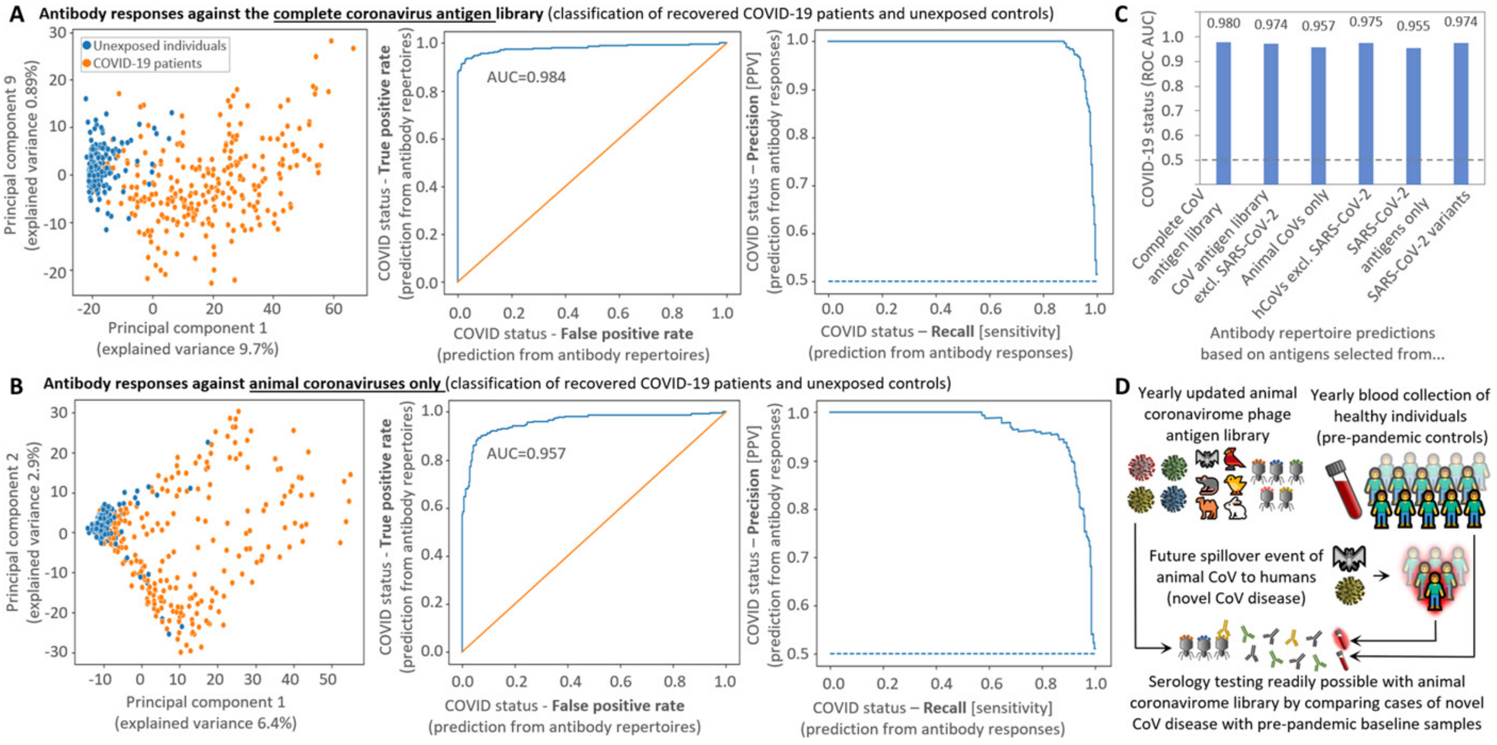

Fig. 7. Antibody responses against antigens of animal coronaviruses allow to accurately identify recovered COVID-19 patients (a-c) suggesting a possible diagnostic strategy for the early stage of future pandemics (d). a,b Classification of recovered COVID-19 patients (n=269) and unexposed controls (n=260) from antibody responses against all antigens of the CoV library (a) and only from the animal coronavirome (b). Principal component analysis (PCA) on fold-changes of antibody responses separates between hCoV antibody responses of unexposed individuals and recovered COVID-19 patients (left panel) and a machine learning predictor accurately identifies infected individuals illustrated by receiver operating characteristic (middle) and precision-recall (right) curves (ROC,PRC). **c** AUC (area under the curve of the ROC [receiver operating characteristic] curve) of predictions with subgroups of antigens of the CoV antigen library. All predictions (including panels a,b) were performed with gradient boosting decision trees (XGBoost classifier (*70*)) with leave-one-out cross-validation from CoV peptides bound in >5% individuals. The aCoVs used for these predictions are limited to RefSeq strains (deposited in 2018) while a bat CoV related to SARS-CoV-2 (*24*) was excluded (Data file S1). **d** Creating regularly updated antigen libraries representing the animal coronavirome alongside the collection of baseline samples of healthy individuals can provide the basis for a serological assay to readily respond in case of a novel zoonotic transmission event.
