# Supplementary material for: Cross-reactive antibodies against human coronaviruses and the animal coronavirome suggest diagnostics for future zoonotic spillovers
Source: Sci Immunol. 2021 Jul 29;6(61):eabe9950. doi: 10.1126/sciimmunol.abe9950 (PMC9267281; doi:10.1126/sciimmunol.abe9950)
Supplement: Supplementary file 2 — Figs. S1 to S11 Table S1 Legends for data files S1 to S4 References (71–73) [file sciimmunol.abe9950_sm.pdf]

Science Immunology
AAAS

Supplementary Materials for

Cross-reactive antibodies against human coronaviruses and the animal coronavirome suggest diagnostics for future zoonotic spillovers

Shelley Klompus *et al.*

Corresponding author: Thomas Vogl, thomas.vogl@weizmann.ac.il; Ziv Shulman, ziv.shulman@weizmann.ac.il; Eran Segal, eran.segal@weizmann.ac.il

Sci. Immunol. **6**, eabe9950 (2021)
DOI: 10.1126/sciimmunol.abe9950

The PDF file includes:

Figs. S1 to S11
Table S1
Legends for data files S1 to S4
References (*71–73*)

Other Supplementary Material for this manuscript includes the following:

Data files S1 to S4

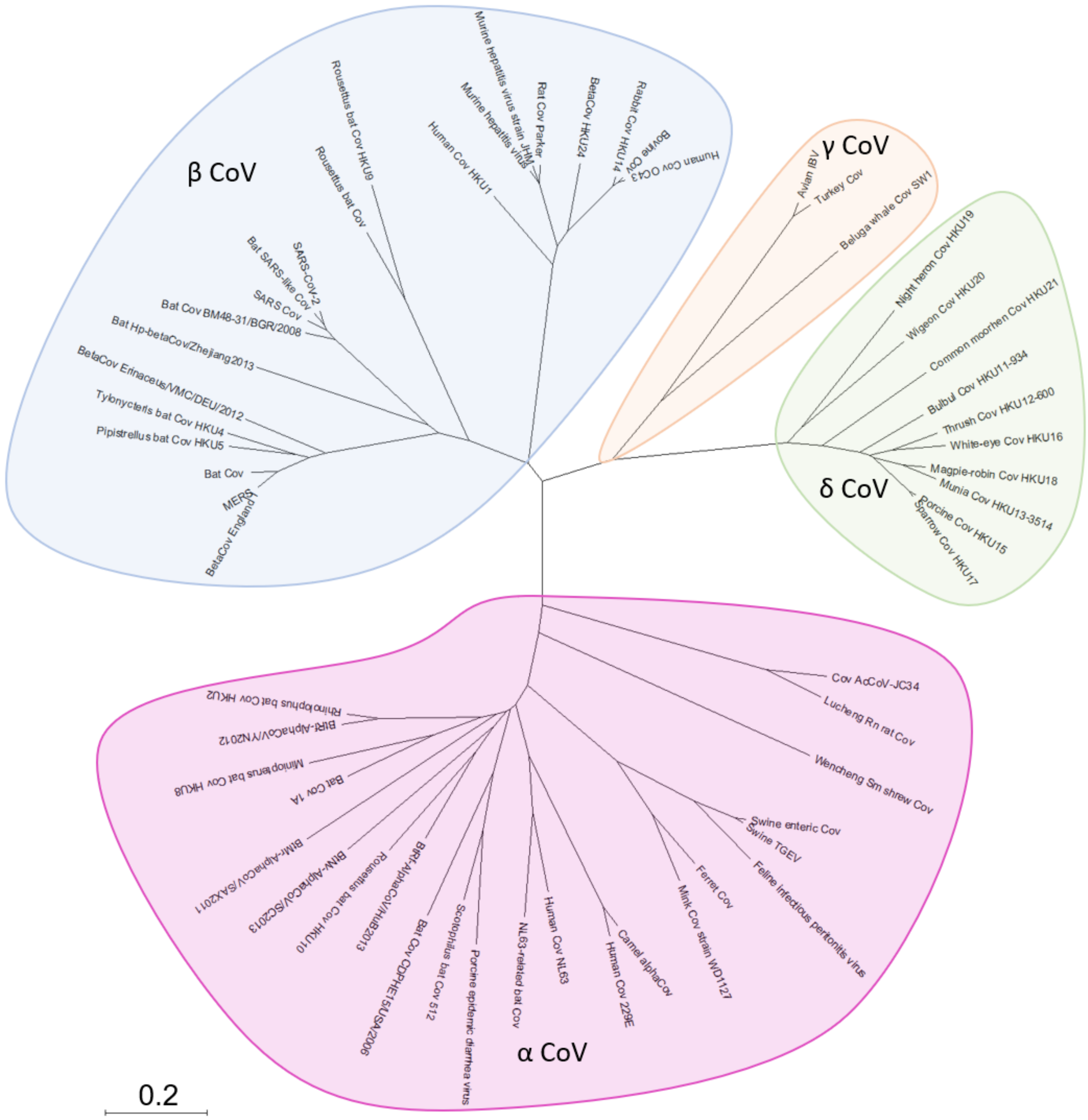

Fig. S1: A phylogenetic tree of all hCoVs and aCoVs included in the phage antigen library. The tree was generated with MEGAX (71) based on nucleocapsid sequences of the respective CoVs (Data file S1). The scale bar indicates the estimated number of substitutions per 20 nucleotides.

a

Antigen details			Antibody responses (in >5% both cohorts)		
Group	Protein information	Number of peptides per protein/group	≥1 peptide per protein		Number of peptides appearing
			Unexposed indiviudals (%)	Recovered COVID-19 patients (%)	
Negative controls	Random peptides (n=42)	42	none	none	0
	Negative control (human SAPK4/MAPK13 protein)	8	none	none	0
	HIV 1 Envelope glycoprotein gp 160	19	7.7	11.5	1
Viral positive controls	Human herpesvirus 3 Envelope glycoprotein C	13	28.1	39.4	1
	Human adenovirus C Pre-histone-like nucleoprotein	5	79.2	71.4	3
	Enterovirus B Genome polyprotein	50	97.7	96.3	13
	Human herpesvirus 1 Envelope glycoprotein D	9	45.8	50.6	3
	Norwalk virus Non-structural polyprotein	38	81.9	85.9	8
	Rhinovirus B Genome polyprotein	50	98.8	97.8	16
	Human herpesvirus 5 Envelope glycoprotein M	8	6.2	10.0	1
	Enterovirus C Genome polyprotein	3	88.5	90.0	1
	Human RSV Attachment glycoprotein	3	41.5	39.8	1
	Influenza A virus Hemagglutinin	7	23.1	21.6	2
	Human herpesvirus 4 eEBV nuclear antigen 1	13	90.0	89.2	5

b

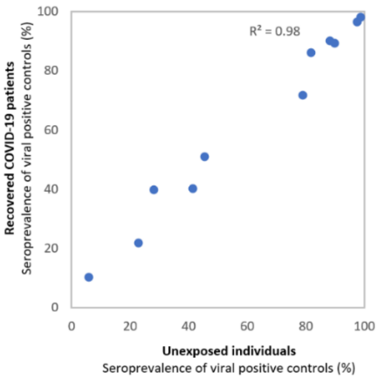

Fig. S2: Control antigens included in the library (a) demonstrate low background signal of unspecific binding against negative controls and reproducible antibody recognition of previously reported viral antigens (*21*) **with similar abundances between unexposed individuals and recovered COVID-19 patients (b).** Peptide negative controls within the hCoV antigen library included 42 random peptides, peptides of the human protein SAPK4 (which should not elicit autoreactive antibodies in healthy individuals), and an HIV protein (with HIV infection being an exclusion criterion for participation in this study). Detected antibody binding against peptides of these proteins is summarized for the cohort of 260 unexposed individuals and 269 recovered COVID-19 patients.

A few random peptides were bound in single individuals, but none peptide were bound in >5% of the cohort (the cutoff applied for selecting peptides for predictions, and for scoring differences in distributions between cohorts [Fig. 7, Data file S3]). Also, peptides of the SAPK4 and HIV protein negative control were bound at low levels (with one HIV peptide appearing in >5% of the cohort, but not being differentially bound between the two groups, possibly owing to over-sensitivity as discussed below). We had included these controls previously in a different library design (244,000 plus 1,807 variants) and had observed lower antibody binding to these controls (*72*). We hypothesized, that the slightly increased levels of bound controls of the library reported in the current paper, could be due to experimental aspects or analysis (scoring of significant peptides) caused by the different library sizes (the current one is with approximately 13,000 variants more than 10-times smaller than the previously used one).
Therefore, we measured 64 samples (32 of unexposed in individuals and 32 of recovered COVID-19 patients) both with 1.) the current library alone (13,192 variants) and 2.) the current library mixed with a larger library of 244,000 variants (equaling to 257,192 variants in total). The mixture with a larger library indeed showed lower binding of random peptides (only 2 peptides being significantly bound in one person, opposed to 7 peptides bound in up to 2 individuals when applying this library alone [data not shown]). While in the mixture with the larger library not a single peptide of the SAPK4 control was bound, 1 peptide was bound in two individuals when using the current library alone. Also, HIV control peptides were only bound in single individuals when working with the mixture with a larger library, while peptides were bound in up to 5 individuals when using the library on its own. Concomitantly, we detected binding against additional CoV peptides (many in regions of high conservation alike Fig. 3 and Fig. 4) only with the current library without mixing it with the larger library. These results suggest that using the smaller library on it is own increases sensitivity at the cost of specificity (with slightly increased binding against negative controls), which is also affected by the larger cohort available when using the full library.
We do not expect that this aspect of using a small library affects key conclusions of our work for the following reasons: Samples of both groups (unexposed controls and recovered COVID-19 patients)

are equally affected and also all peptides of the library (irrespectively of representing hCoVs, aCoVs, or controls) are equally affected. Hence separating the groups by machine learning (Fig. 7) is equally affected by the same biases. Furthermore, we have set cutoffs to exclude responses as low, or lower than random peptides (*i.e.* calculations of significantly enriched peptides [Data file S3] and predictions [Fig. 7] are performed only on peptides appearing in >5% of individuals). If antibody binding should be assessed as peptides summed per protein (as shown for the controls above), applying a cutoff of at least two or more peptides appearing per protein can increase specificity (although also true binding events may be lost in a tradeoff for sensitivity, as not all antigen may contain ≥2 epitopes detectable by PhIP-Seq). From a technical perspective, it may be possible to improve the accuracy of rather small PhIP-Seq libraries by including larger numbers of random "dummy" peptides to improve accuracy and reduce oversensitivity. Yet, some remaining cross-reactivity of serum antibodies (consisting a mixture of immunoglobulins possibly raised upon lifelong exposure to potentially tens of thousands or hundreds of thousands of antigens) with negative controls appears possible, as experiments with mAbs showed that 1 out of 19 bound peptides showed rather low identity to the consensus motif (despite being significantly bound [peptide #7309 in Fig. **S 8**c]).

Serum prevalence for non-CoV viral antigens were similar between unexposed individuals and recovered COVID-19 patients (panel b). The slight differences of seroprevalence in unexposed individuals and recovered may be due to minor variations in cohort sizes or age/gender distributions.

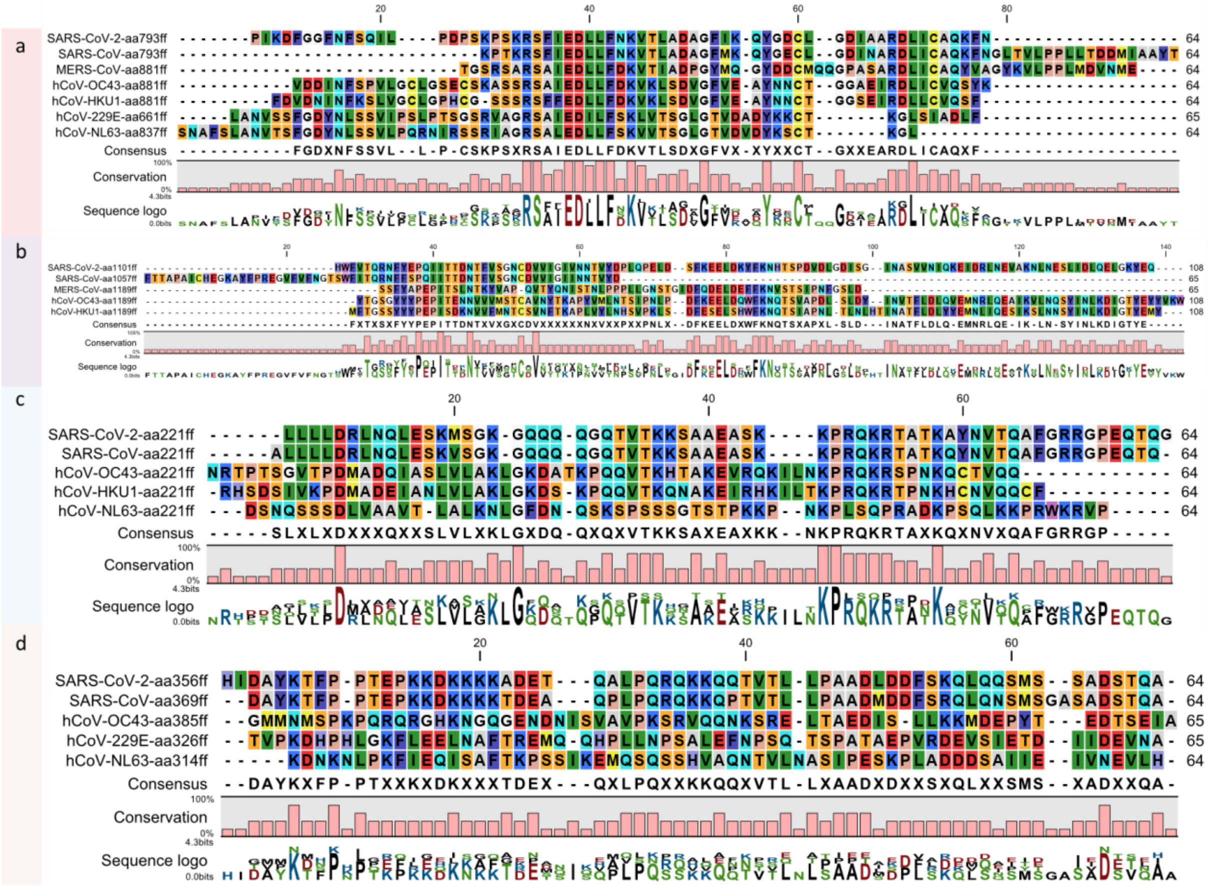

Fig. S3: Full alignments of hCoV peptides shown in Fig. 3 following the same color coding with 2 regions each in the spike (a,b) and nucleocapsid (c,d) proteins highlighted. Alignments were generated with MegaX (ClustalW algorithm in standard settings) and visualized with CLC Main Workbench 6. In panel b adjacent peptides bound in the same hCoV strain were merged. The starting amino acid (aa) of each peptide in its originating hCoV is indicated in the label on the left.

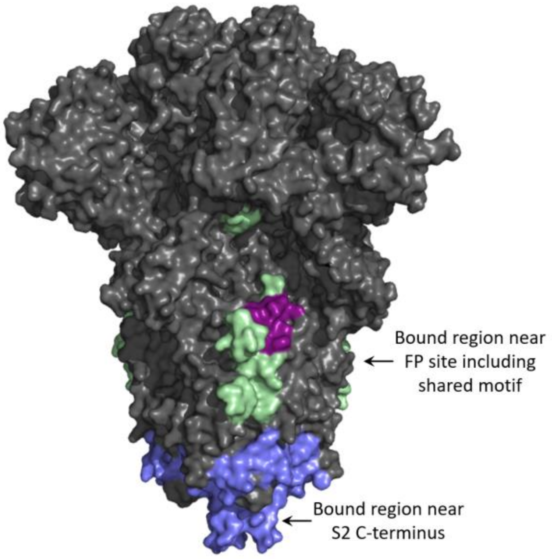

Fig. S4: Peptides bound by antibodies in recovered COVID-19 patients and featuring conserved motifs between hCoVs and aCoVs lie on the surface of the SARS-CoV-2 spike protein. Peptides of SARS-CoV-2 bound differentially in unexposed individual and COVID-19 patients (Fig. 3a) as well as featuring conserved motifs of other hCoVs (Fig. 3c,d) and aCoVs (Fig. 4a,e,f) are projected on a crystal structure of the SARS-CoV-2 spike protein (PDB accession number 6VXX -spike in closed conformation (2)). A peptide spanning the FP site is marked in green, with the conserved motif (RSXIEDLLFXK) mentioned in the main text highlighted in purple (peptide #8148 starting at aa 792 of the SARS-CoV-2 spike protein [full details in Data file S2]:
PIKDFGGFNFSQILPDPSKPSK**RSFIEDLLFNK**VTLADAGFIKQYGDCLGDIAARDLICAQKFN
the underlined amino acids were projected onto the figure, the above-mentioned motif [shown in purple] is highlighted in bold, for unmarked AAs densities are missing).
Another region near the S2 C-terminus is marked in blue (peptide #4017 starting at aa 1100 of the SARS-CoV-2 spike protein [full details in Data file S2]:
HWFVTQRNFYEPQIITTDNTFVSGNCDVVIGIVNNTVYDPLQPELDS**FKEELDKYFKN**HTSPDV, the shared motif in this region (bold) highlighted in Fig. 4f falls in an area of missing density).

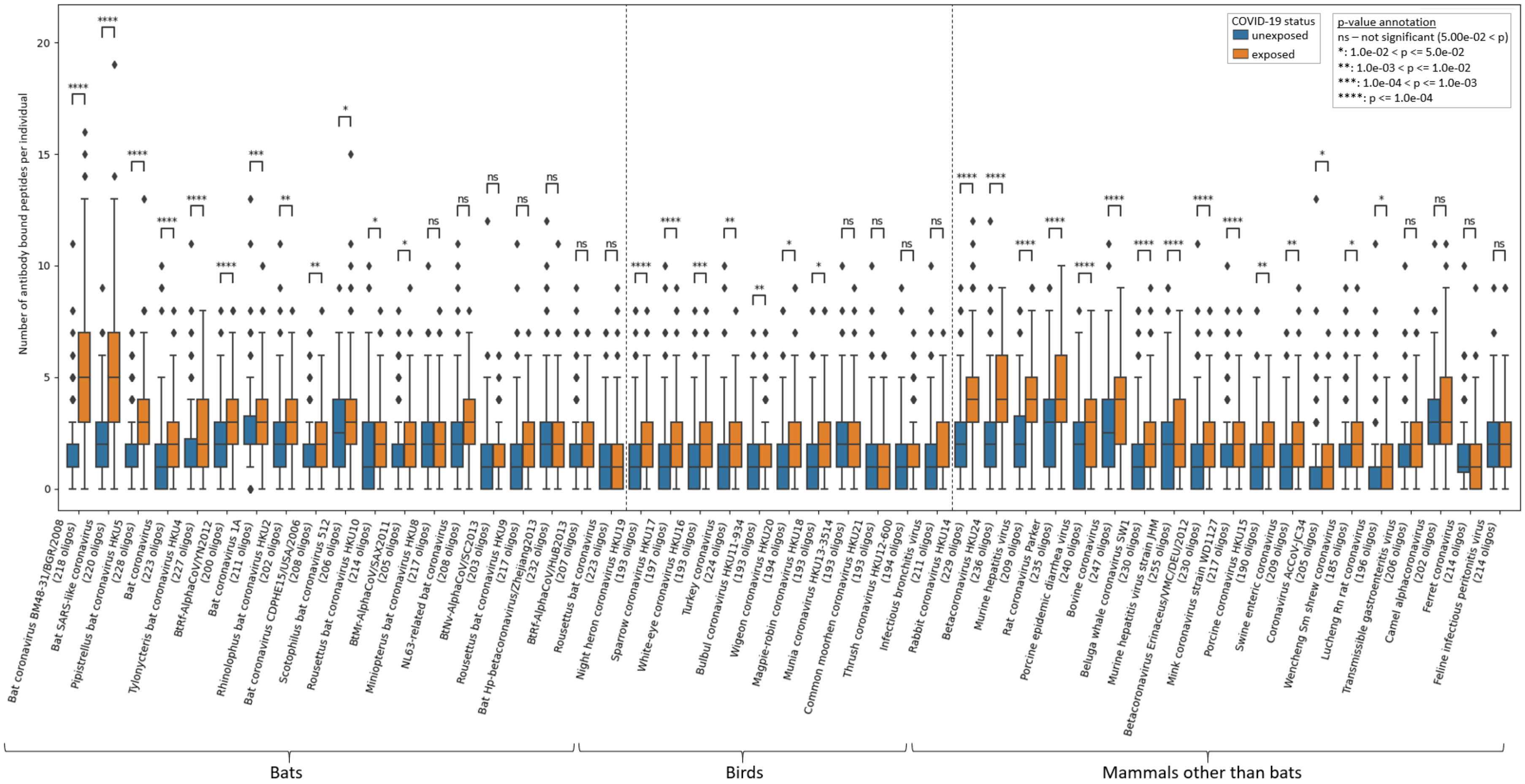

Fig. S5: Antibody responses against antigens of the 49 animal coronaviruses included within the library (Fig. 1b) compared between recovered COVID-19 patients (n=269) and unexposed individuals (n=260). These results are summarized for the three groups indicated in Fig. 4a. The number of oligoes included for each strain in parentheses refers to peptides included in the input antigen library before testing for antibody binding. The number of bound peptides per individual is plotted for each strain on the y-axis. The center line shows the median; box limits indicate the 25th and 75th percentiles as determined by Seaborn software; whiskers extend 1.5 times the interquartile range from the 25th and 75th percentiles, outliers are represented by dots. Significance between the groups was calculated with the Mann-Whitney test.

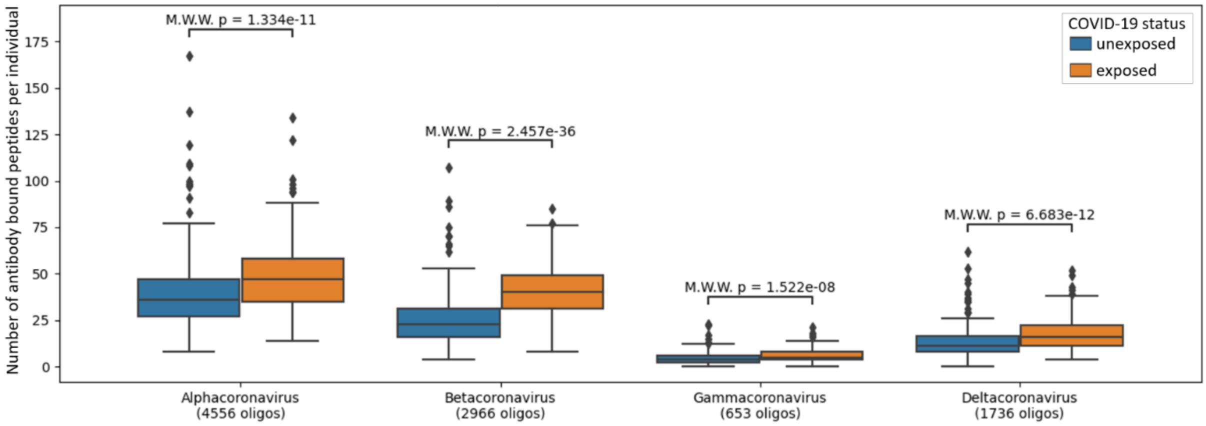

Fig. S6: Antibody responses against aCoVs by genetic distance and grouping of α, β, γ, and δ aCoVs. These results are summarized by the classification of aCoVs as α, β, γ, and δ aCoVs indicated in Data file S1. Antibody binding data of the full set of 269 recovered COVID-19 patients and 260 unexposed individuals is shown. The number of oligoes included for each group in parentheses refers to peptides included in the input antigen library before testing for antibody binding. The number of antibody bound peptides per individual is plotted for each group on the y-axis. The center line shows the median; box limits indicate the 25th and 75th percentiles as determined by Seaborn software; whiskers extend 1.5 times the interquartile range from the 25th and 75th percentiles, outliers are represented by dots. Significance between the groups was calculated with the Mann-Whitney test.

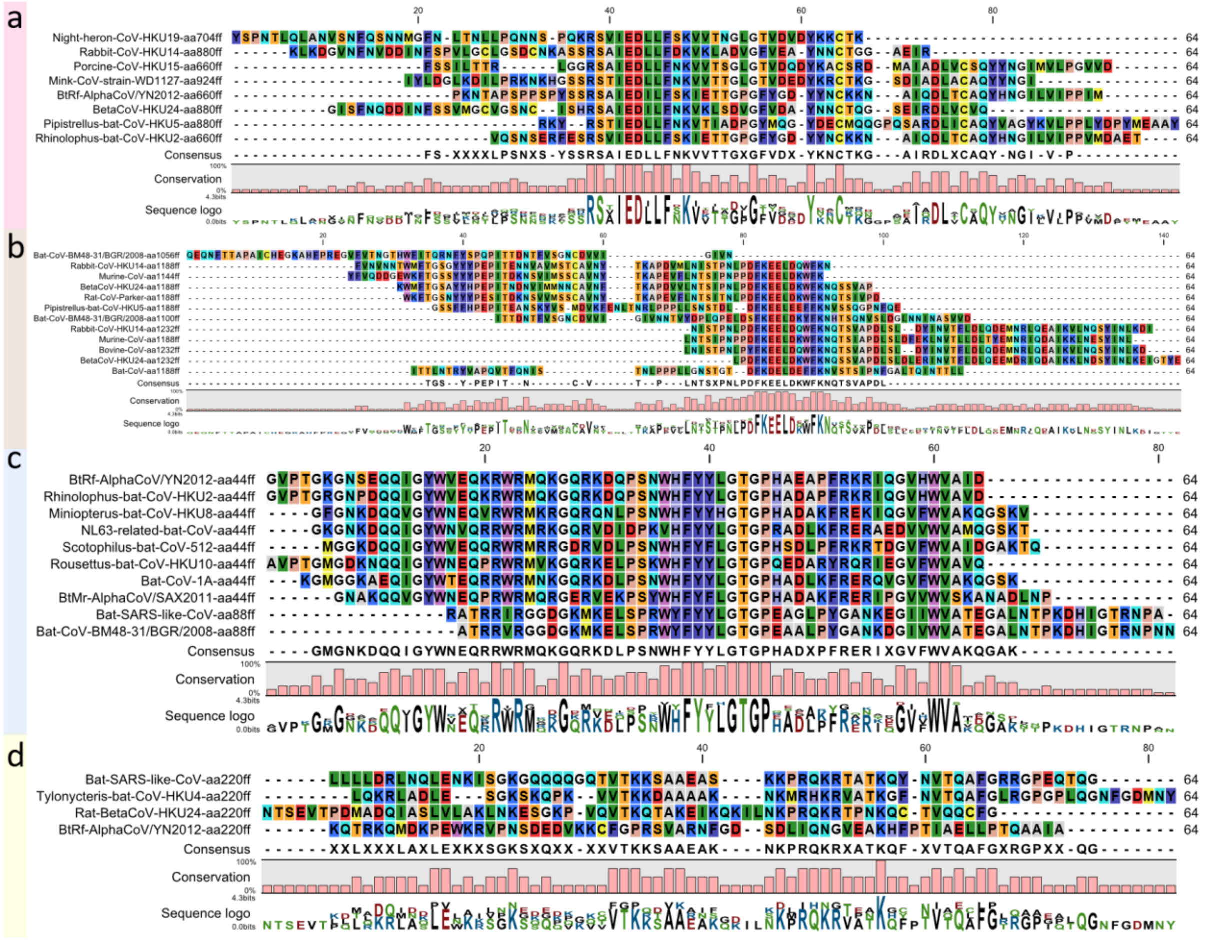

Fig. S7: Full alignments of aCoV peptides shown in Fig. 4 following the same color coding with 2 regions each in the spike (a,b) and nucleocapsid (c,d) proteins highlighted. Alignments were generated with MegaX (ClustalW algorithm in standard settings for panels a,c,d and MUSCLE algorithm in standard settings for panel b) and visualized with CLC Main Workbench 6. The starting amino acid (aa) of each peptide in its originating hCoV is indicated in the label on the left.

a

Peptide number in library	Antigen information							-log10 values of peptide in PhIP-Seq			
	Virus name	Host	Protein	Protein accession number	Protein length	Starting position of peptide in the protein	Host type	mAb WIS-C1		mAb WIS-C3	
								R1	R2	R1	R2
121	Thrush coronavirus HKU12-600	grey-backed thrush	spike	YP_002308497.1	1192	880	bird	200.0	197.3	35.2	38.9
8,751	Rabbit coronavirus HKU14	rabbit	spike	YP_005454245.1	1362	1056	Other mammals	198.8	200.0	200.0	198.3
324	Human coronavirus HKU1	human	spike	YP_173238.1	1356	1056	human	197.9	185.3	198.4	197.7
3,099	Rat coronavirus Parker	rat	spike	YP_003029848.1	1360	1056	Other mammals	197.8	197.6	197.5	197.5
2,601	Betacoronavirus HKU24	rat	spike	YP_009113025.1	1358	1056	Other mammals	197.6	197.7	197.6	197.8
4,473	Human coronavirus OC43	human	spike	YP_009555241.1	1353	1056	human	197.6	197.7	197.6	197.8
7,349	SARS-CoV-2	human	spike	multiple, see S 3	1273	968	human	197.6	165.4	197.7	197.6
2,602	SARS-CoV-2	human	spike	QHS34546.1&QIX12148.1	1272	968	human	197.3	200.0	200.0	197.1
6,090	Bovine coronavirus	cow	spike	NP_150077.1	1363	1056	Other mammals	197.3	200.0	200.0	197.3
7,350	SARS-CoV-2	human	spike	pdb|6VSB|C	1280	968	human	197.3	200.0	98.4	103.4
7,227	Murine hepatitis virus strain JHM	mouse	spike	YP_209233.1	1376	1056	Other mammals	197.2	200.0	200.0	197.9
3,805	Rousettus bat coronavirus	bat	spike	YP_009273005.1	1290	968	bat	89.9	46.1	23.6	19.0
7,705	Bat SARS-like coronavirus	bat	spike	AVP78042.1	1245	924	bat	55.6	22.8	27.1	29.5
1,875	SARS-CoV-2	human	spike	pdb|6VXX|C&pdb|6VYB|C	1273	968	human	44.8	35.0	2.4	2.8
10,239	SARS-CoV	human	spike	NP_828851.1	1255	924	human	34.8	14.8	23.6	26.9
10,078	Bat coronavirus BM48-31/BGR/2008	bat	spike	YP_003858584.1	1259	924	bat	34.7	19.5	17.1	21.3
7,309	SARS-CoV-2	human	spike	pdb|6LXT|F	132	68	human	33.8	32.5	0.4	1.0
8,712	Wencheng Sm shrew coronavirus	shrew	polyprotein 1ab	YP_009389424.1	6324	6028	Other mammals	18.0	19.2	0.2	1.4
9,597	Scotophilus bat coronavirus 512	bat	spike	YP_001351684.1	1371	1056	bat	15.5	12.4	0.4	0.4

Alignment in b

Alignment in c

b

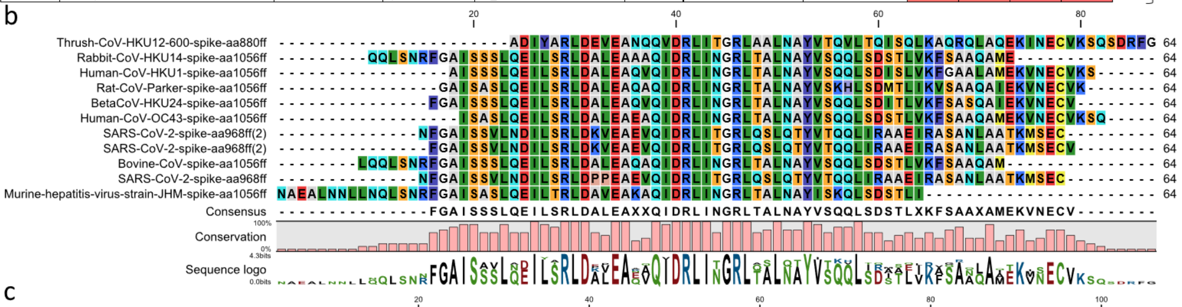

c

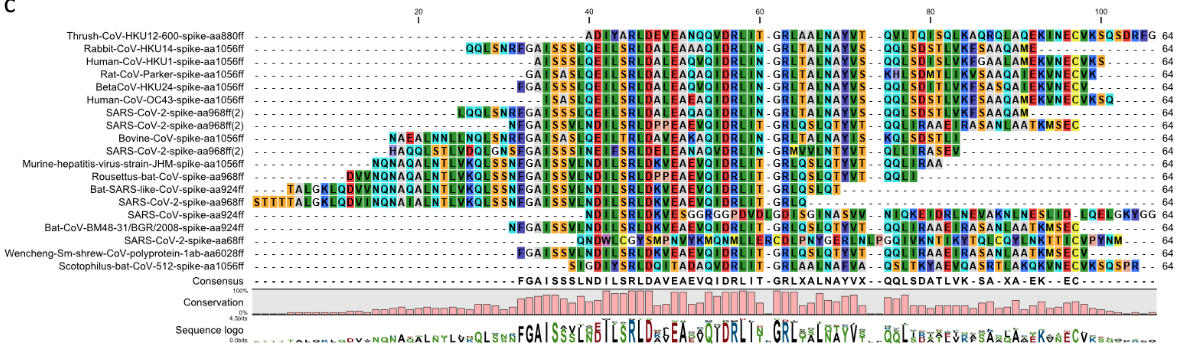

Fig. S8: List of peptides significantly bound by the mAbs WIS-C1 and WIS-C3 (plotted in Fig. 6c) and alignments (subsection shown in Fig. 6e). a The two mAbs were processed with the PhIP-Seq library as outlined in the caption of Fig. 6. The two technical duplicates (R1,R2) for each mAb /Fig. 6c) are in excellent agreement. The p-values are computed by scoring the reads of each sample after PhIP-Seq against the input sequencing distribution of the library (as outlined in the materials and methods section). The maximum p-values computed are cut off at -log10 200. **b,c** Alignments were generated with MegaX (ClustalW algorithm in standard settings for panels b and MUSCLE algorithm in standard settings for panel c) and visualized with CLC Main Workbench 6. The starting amino acid (aa) of each peptide in its originating hCoV is indicated in the label on the left.

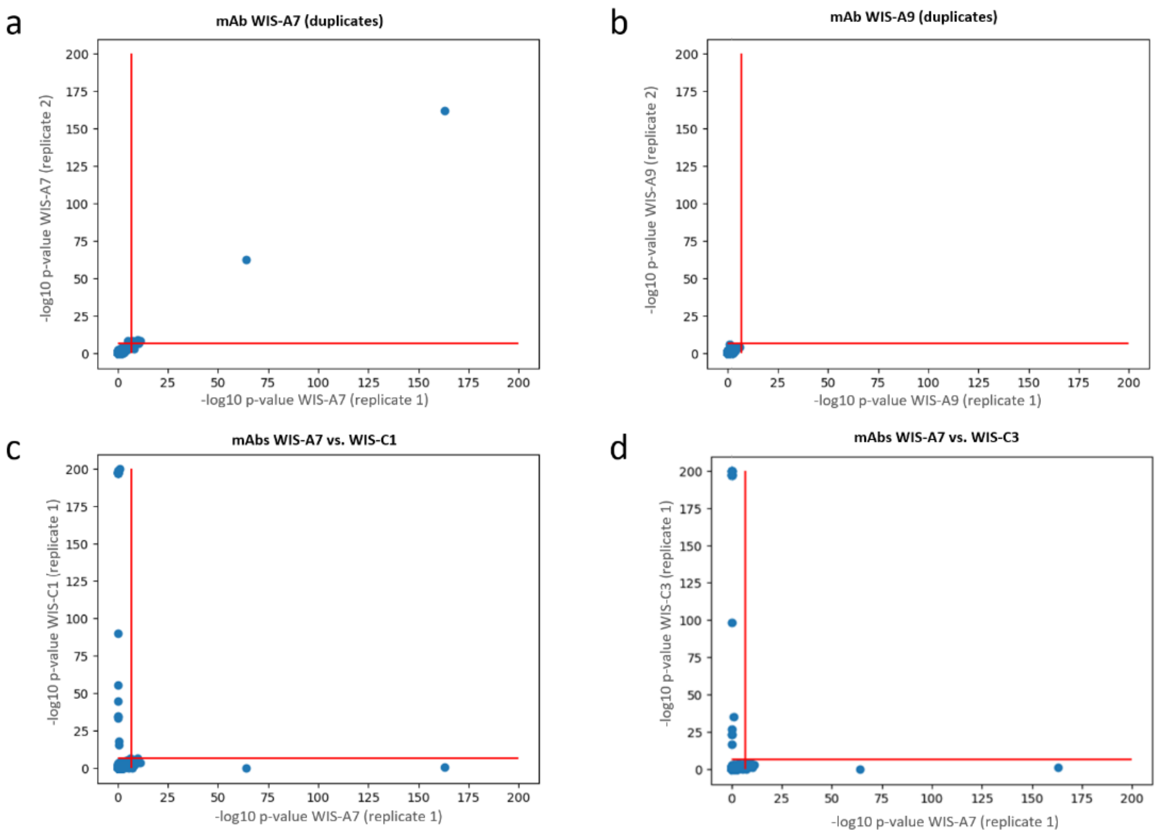

e Alignment of peptides bound by WIS-A7

```
SARS-CoV-2-spike-aa748            SMTKTSVDCTMYICGDSTECSNLLLQYGSFCTQLNRALTGIAVEQDKNTQEVFAQVKQIYKTPP---
Bat-SARS-like-CoV-spike-aa704     ---KTSVDCTMYICGDSIECSNLLLQYGSFCTQLNRALSGIAIEQDKNTQEVFAQVKQIYKTPPIKD
                                     ************** ********************:***:*********************
```

Fig. S9: PhIP-Seq based identification of targeted peptides within the CoV-antigen library of two additional human mAbs WIS-A7 (a) and WIS-A9 (b), comparison of the peptides bound by WIS-A7 to WIS-C1 (c) and WIS-C3 (d) as well as an alignment of the two peptides bound by WIS-A7 (e). The two mAbs were mixed with the phage displayed antigen library and processed in the same way as serum samples. Reaction mixtures were set up in duplicates and significantly bound peptides are marked (threshold indicated by red line). The maximum p-values computed are cut off at -log10 200. In panel e the two peptides bound by WIS-A7 are aligned (CLUSTAL O(1.2.4) multiple sequence alignment).

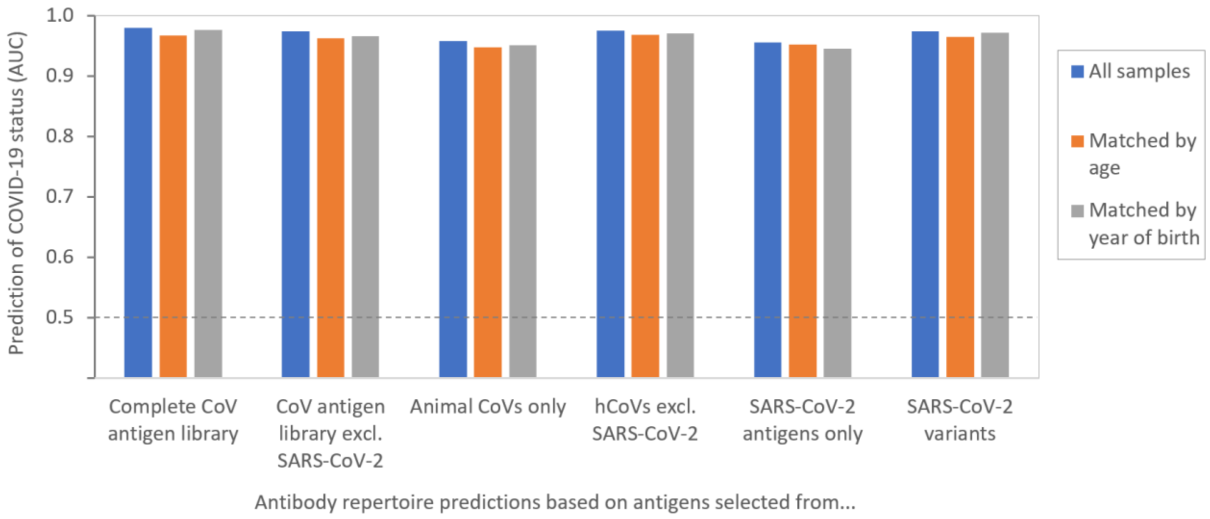

Fig. S10: Matching recovered COVID-19 patients and unexposed controls by age or year of birth does not affect the accurate separation of the two groups by a machine learning classifier from antibody repertoire data. See Data file S3 for details on the respective matched cohorts and Fig. 4 plus accompanying text for the reasoning behind the predictions from library subgroups. See Fig. 1c for the age gender distribution of the full cohort ('All samples'). Age matched: 366 individuals in total (183 each recovered COVID-9 patients and unexposed controls), COVID-19 recovered: age range 17.0-71.0 years, average 36.8+-11.6 years, gender 59 male / 124 female; Unexposed controls: Age range: 17.0-70.0 years, average 36.7+-11.7 years, gender: 59 male /124 female. Matched by year of birth: 382 individuals in total (191 each recovered COVID-9 patients and unexposed controls), COVID-19 recovered: age range 23.0-73.0 years, average 41.8+-11.2 years, gender 54 male / 137 female; Unexposed controls: Age range: 17.0-70.0 years, average 36.2+-11.0 years, gender: 54 male /137 female.

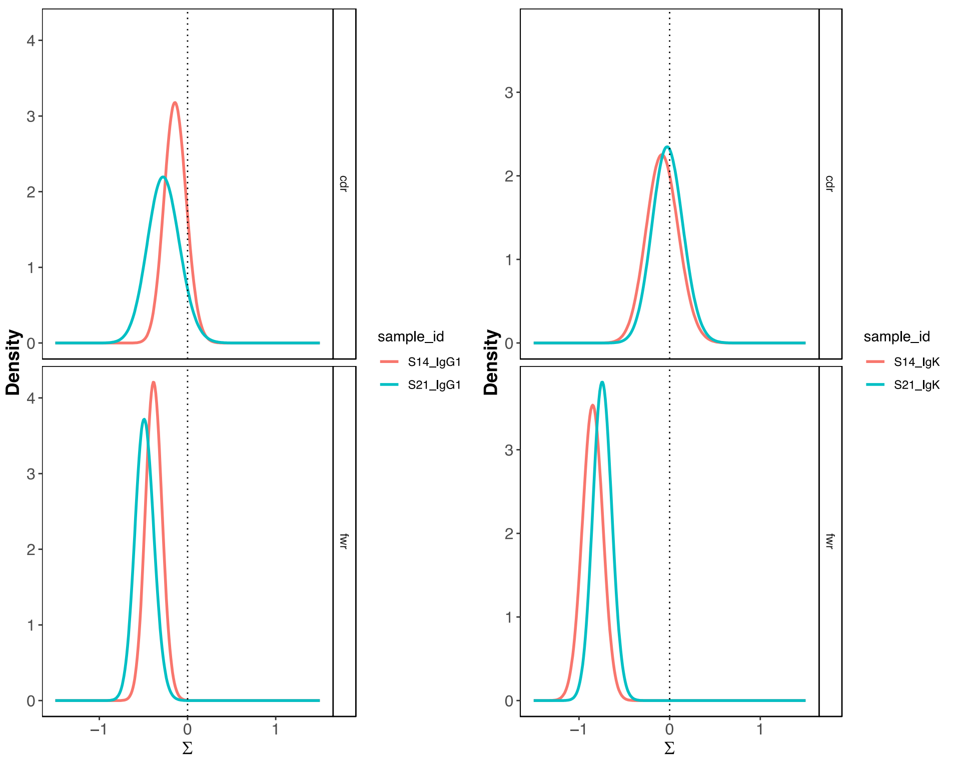

Fig. S11: Affinity driven selection quantification. Posterior probability density function quantifying affinity driven selection is shown for two patients: S14 and S21 (red and blue curves respectively). Left panels show the selection estimations in the heavy chain, and right panels show the same for the Kappa chain. Upper panels show the estimation for CDRs and lower panels for FWRs. All results were obtained by applying the local test of BASELINe (*39*) using the S5F mutability model (*40*).

Study	Dominant VH	Dominant VL	% expanded clones	Average germline identity VH	Average germline identity VL
Robbiani *et al.* (32)	VH3-30, VH3-53	LV3-21, LV1-40, LV6-57	25%-60%	98%	99%
Wec *et al.* (34)	VH1-69	VK2-30	50%	92%	-
Kreer *et al.* (35)	IGHV3-49, IGHV3-30	IGKV4-1	25%-45%	98%	92%
Rogers *et al.* (73)	IGHV1-2*02	IGLV2-14*01	30%	98%%	1.70%
The current study	VH3-30	VK3-20	84% and 12.6%	95%	97%

Table S1: Comparison of the current findings to previous studies.

Data file S1: Supporting .xlsx file with a list of the human and animal coronaviruses included in the antigen phage library. GenBank accession numbers are provided and also the dates, when the genomes have been deposited are include. The majority of the CoVs were obtained from NCBI RefSeq genomes (Coronaviridae, taxid 10239).

Data file S2: Supporting .xlsx file with a full list of peptides included within the library as well as the corresponding amino acid sequences (including controls). For each displayed peptide the viral strain and host of origin and protein name (including the accession number in the relevant databases NCBI/UniProt/GISAID) is provided. Starting positions of the peptides in the protein's sequence are provided.

Data file S3: Supporting .xlsx file with most frequent hCoV/aCoV antigens bound in unexposed individuals and recovered COVID-19 patients. Abundance of antibody responses against peptides of hCoV proteins detected in more than 5% of individuals are listed. Multiple peptides originating from the same protein are written adjacently. In a few cases identical peptides arose from different proteins (such as an identical region of SARS-CoV-2 and a bat CoV), which is marked with double names (separated by '&'). The originating proteins may have exhibited slightly different total lengths, hence also the respective shift of the starting position of the peptides is indicated. For SARS-CoV-2, several variants of strains in addition to the reference strain (listed in Data file S1) were included. Also, in these cases, due to deletions and truncations, the starting position of identical peptides could be shifted, which is indicated by providing both numbers (separated by '&').

Peptides bound at significantly different percentages between unexposed individuals ('U') or recovered COVID-19 patients ('C') are indicated (Chi-squared test on peptide appearance, as well as Kolmogorov–Smirnov test on the difference between the two distributions of the log fold changes (number of reads of bound peptides vs. baseline sequencing of phages not undergoing IPs) of the two groups. Peptides passing FDR (false discovery rate) are marked in a separate column. Testing for significance was performed on the full cohort (U=260, C= 269) as well as cohorts matched by age (U=191, C=191) or year of birth (U=183, C=183). In Fig. 3a,b and Fig. 4c,d only peptides passing FDR correction for both tests (Chi-squared test as well as Kolmogorov–Smirnov) in all three cohorts (full cohort, matched by age, matched by year of birth [yob]). Matching by age and year of birth was performed separately, as both biological age and exposure to CoVs in certain periods could bias results (e.g. individuals born in the 1980s may have been exposed to different seasonal CoVs than individuals born in the 1990s). Neither matching by age or year of birth did affect the conclusions (shown in detail in Fig. S10); 'n.a.' in the age/yob columns indicates peptides that did not appear in >5% of these cohorts compared to the full cohort (peptides appearing only in >5% of age/yob matched cohorts but not in the full cohort are excluded).

On the rightmost side of the table, alignment details (using the BLAST algorithm) of the respective peptides to the SARS-CoV-2 proteome are provided. This information was used for generating Fig. 4c,d and also illustrates differences of SARS-CoV-2 variants compared to the reference genome. 'n.a.' in the alignment columns indicates if no alignment could be created.
